# REBCO superconductors by pulsed laser deposition: Key innovations and large-scale applications

**DOI:** 10.1016/j.isci.2025.113260

**Published:** 2025-08-05

**Authors:** Albina Jetybayeva, Aliya Mukanova, Arailym Nurpeissova, Zhumabay Bakenov, Valery Petrykin, Sergey Lee

**Affiliations:** 1 National Laboratory Astana, 53 Kabanbay Batyr Avenue, Astana 010000, Kazakhstan; 2 Institute of Batteries LLC, 53 Kabanbay Batyr Avenue, Astana 010000, Kazakhstan; 3 Department of Chemical and Materials Engineering, Nazarbayev University, 53 Kabanbay Batyr Avenue, Astana 010000, Kazakhstan; 4 Faraday Factory Japan LLC, 1F, FINE Bldg. 2956-6 Ishikawamachi, Hachioji, Tokyo 192-0032, Japan

**Keywords:** Physics, Condensed matter physics, Superconductivity

## Abstract

The rapid advancement of second-generation high-temperature superconductors, especially REBCO materials, underpins innovations in energy, medicine, and transport. Pulsed laser deposition (PLD) stands out as a premier method for producing high-quality REBCO thin films with controlled composition and microstructure—crucial for achieving high critical current density (Jc) and temperature (Tc). This review synthesizes recent progress in PLD-grown REBCO films, detailing how parameters such as laser energy, deposition temperature, background pressure, and substrate design influence superconducting performance. Special attention is given to nanoengineering, doping, and multilayer techniques that enhance flux pinning, as well as innovations in PLD setups—including multi-beam and high-rate processes—to enable industrial-scale production. The review also discusses the integration of artificial intelligence and machine learning for optimizing film properties and accelerating discovery. By comparing strategies and results from academia and industry, this work provides a comprehensive roadmap for advancing PLD-based REBCO superconductor technology.

## Graphical abstract

**Figure:**
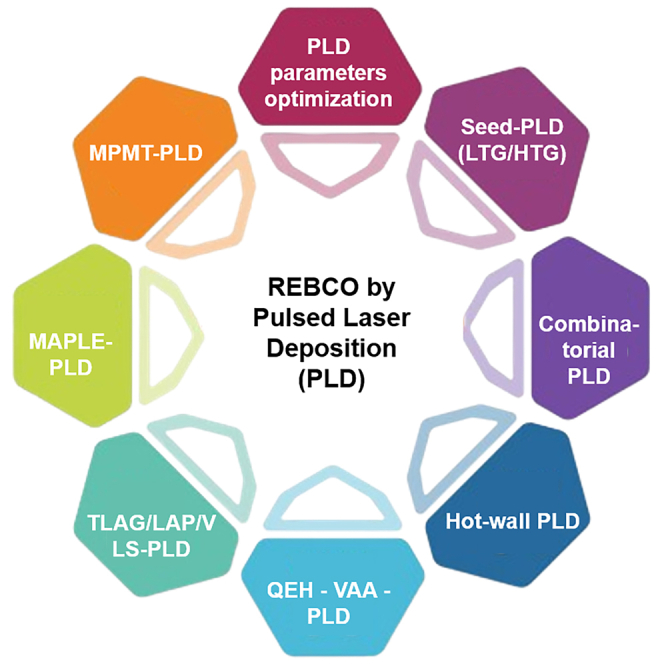

## Introduction

The pursuit of high-temperature superconductors (HTS) has long stood at the forefront of materials science, driven by their transformative potential across diverse technological sectors.^1^^,^^2^^,^^3^^,^^4^^,^^5^^,^^6^^,^^7^ Second-generation HTS (2G-HTS) represent a major leap forward, offering enhanced performance, greater efficiency, and compact design possibilities for critical applications ranging from energy transmission and medical imaging to cutting-edge scientific instrumentation.^1^^,^^2^^,^^3^^,^^5^^,^^6^^,^^7^

**Figure 1:**
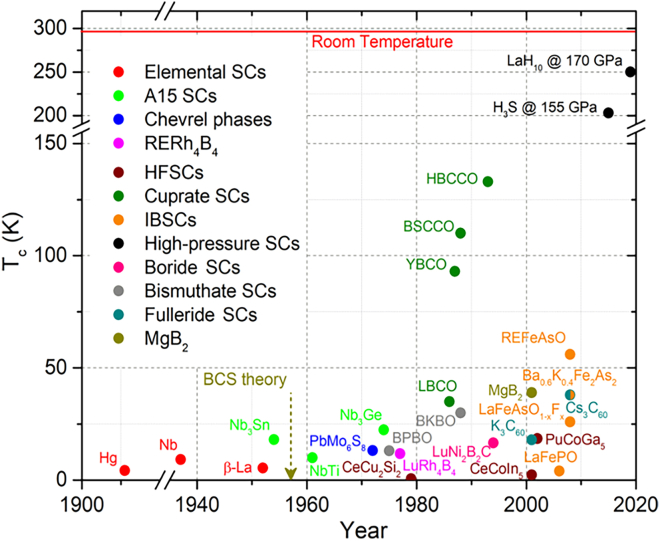
Timeline of several superconductor discoveries marked as before and after BCS theory SCs: superconductors. A15 SCs: superconductors deriving from Cr_3_Si structure type. Chevrel phases: MMo_6_S_8_, where M = Pb and rare-earth (RE). RERh_4_B_4_: RE represents rare-earth elements. HFSCs: heavy fermion superconductors. LBCO: Ba-doped La_2_CuO_4_ (LBCO). YBCO: YBa_2_Cu_3_O_7−δ_. BSCCO: Bi_2_Sr_2_Ca_n−1_Cu_n_O_2n+4_ where n is positive integer. HBCCO: Hg_m_Ba_2_Ca_n−1_Cu_n_O_2n+m+2_, where m = 1 and *n* = 1–7. IBSCs: iron-based superconductors. Reprinted from^6^ with permission from American Chemical Society.

The field was revolutionized in 1986 by the seminal discovery of superconductivity in lanthanum-barium copper oxide by J. G. Bednorz and K. A. Müller,^8^ marking the first observation of superconductivity at a critical temperature (T_n_ > 30 K) well above that of conventional superconductors. This breakthrough challenged existing theoretical paradigms and spurred the discovery of an entire class of superconducting cuprate oxides. Remarkably, these materials exhibited critical temperatures exceeding 77 K—the boiling point of liquid nitrogen—rendering cryogenic cooling significantly more practical for real-world applications (Figure 1).^3^^,^^6^ Subsequent developments led to the emergence of high-performance superconducting systems such as YBa_2_Cu_3_O_7-x_ (YBCO) and Bi-Sr-Ca-Cu-O (BSCCO), which further elevated critical temperatures and solidified the practicality of HTS technologies.^9^^,^^10^ These advancements have not only deepened our understanding of quantum materials but also enabled the translation of HTS from laboratory phenomena to robust components in modern industrial and technological systems.

Among the diverse families of HTS, rare earth barium copper oxide (REBCO or RE-123, where RE (rare-earth) = Y, Sm, Gd, Eu, etc.) stands out due to its exceptional superconducting properties. These materials demonstrate the ability to operate at elevated temperatures and under high magnetic fields, making them the subject of intensive research and widespread technological interest.^2^^,^^3^^,^^5^ The advancement of REBCO-based superconductors has already significantly propelled the field forward, enabling practical applications in technologies, such as magnetic resonance imaging (MRI), power transmission cables, fault current limiters, and more.^6^^,^^7^^,^^11^^,^^12^^,^^13^^,^^14^^,^^15^^,^^16^^,^^17^ Notably, the work of MacManus-Driscoll and Wimbush has clearly delineated the application-specific use of superconducting properties and strategies for enhancing the critical current density (J_a_).^2^

The unique advantages of REBCO materials—particularly their high critical current density—have positioned them as promising candidates for next-generation superconducting technologies. However, challenges remain, including intrinsic brittleness and a pronounced sensitivity to defects.^5^ As such, the optimization and development of REBCO superconductors continue to be active areas of research and industrial innovation.

A critical factor in realizing the full potential of REBCO lies in the synthesis approach, as fabrication methods directly impact microstructural quality and superconducting performance. Moreover, engineering applications demand scalable, high-quality, and cost-effective production techniques. Over the years, various synthesis methods have been developed to meet these needs. Among the most prominent are physical vapor deposition (PVD) techniques such as pulse lased deposition (PLD), sputtering, evaporation, cathodic arc deposition, and molecular beam epitaxy (MBE). Chemical vapor deposition (CVD), including its variants like plasma-enhanced CVD (PECVD) and metal-organic CVD (MOCVD), also plays a key role. Additionally, atomic layer deposition (ALD) is employed for its precise control over film thickness, while sol-gel processes have proven effective, particularly for the preparation of complex oxide superconductors.^18^^,^^19^^,^^20^^,^^21^^,^^22^^,^^23^^,^^24^^,^^25^^,^^26^

Superconductors synthesized via PLD represent a vital frontier in materials science, especially in the context of HTS. PLD involves a highly controlled process in which intense laser pulses ablate material from a solid target, resulting in the deposition of a thin film onto a substrate.^27^^,^^28^^,^^29^ This technique has garnered substantial attention due to its ability to fabricate high-quality thin films with exceptional precision in both composition and thickness.^2^^,^^25^^,^^30^

**Figure 2:**
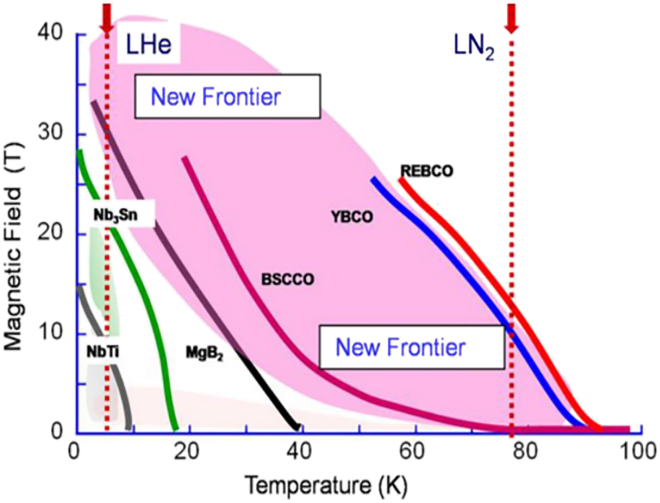
Variation of irreversibility field with temperature for different superconductors, including HTS, MgB2, metallic compounds, and alloys Reproduced from^31^ with permission from Frontiers.

The inherent versatility of PLD makes it particularly well-suited for the growth of complex oxide superconductors—materials that are essential for next-generation electronic, energy, and magnetic technologies. Figure 2 illustrates the irreversibility lines of various superconductors, highlighting the superior performance characteristics achievable through advanced fabrication methods.^31^

Recent research efforts are increasingly focused on scaling PLD for large-area applications, with particular emphasis on the production of REBCO-based coated conductors. A promising direction involves the integration of artificial pinning centers (APCs) within the superconducting matrix, which significantly enhances flux pinning and critical current performance. This approach is poised to enable superconducting applications over a broad temperature spectrum, spanning from 5 K to 77 K, thereby expanding the utility of HTS materials across a wide range of operational environments.

This review aims to provide a comprehensive exploration of the PLD technique, with a particular focus on its application in the fabrication of 2G-HTS, specifically REBCO compounds. The discussion will delve into the synthesis mechanisms, critical processing parameters that govern film quality, and the technological enhancements that have propelled PLD as a leading method for producing high-performance superconducting films. Special emphasis will be placed on the optimization strategies and structural modifications of PLD systems that enable precise control over film architecture and functionality. In-depth analyses of the specific atomic-level effects on superconducting properties, including detailed physical models, have already been reviewed elsewhere and therefore fall outside the scope of the present work.^5^^,^^6^^,^^7^^,^^17^^,^^30^^,^^31^^,^^32^^,^^33^^,^^34^^,^^35^^,^^36^^,^^37^

The review will also address the industrial-scale production of REBCO films via PLD, underscoring the importance of scalability for their implementation in real-world superconducting devices and power applications. Furthermore, the integration of artificial intelligence and machine learning (ML) into the research and development pipeline will be examined. These emerging technologies hold great promise in accelerating the discovery, design, and optimization of superconducting materials by enabling predictive modeling and intelligent process control under varying experimental conditions. Through the examination of these key areas, this review seeks to illuminate the pivotal role of PLD in advancing HTS research and to highlight its transformative potential in enabling future breakthroughs in superconductivity.

## PLD method: Key principles, benefits, and challenges

**Figure 3:**
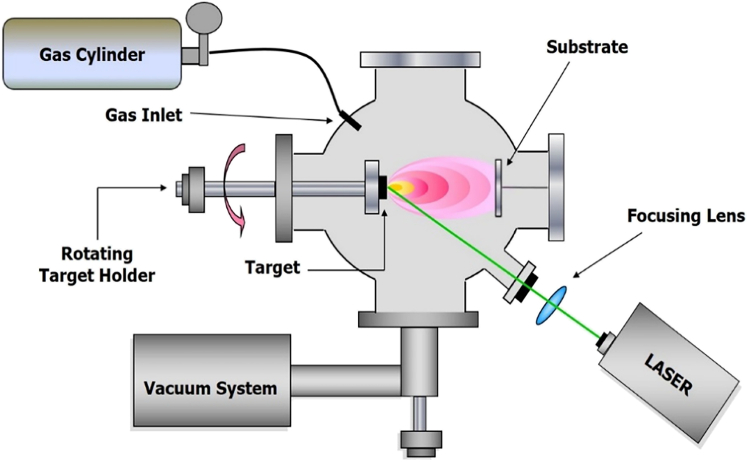
Typical PLD setup scheme Reproduced from^40^ with the permission from MDPI.

One of the principal PVD techniques employed in the fabrication of HTS is PLD. This versatile method enables the precise deposition of superconducting thin films directly from bulk targets.^29^^,^^38^^,^^39^ PLD operates by using high-energy laser pulses to ablate material from a target, which is then deposited onto a substrate to form thin films (Figure 3).^27^^,^^28^^,^^29^ Due to its high deposition rates, excellent stoichiometric transfer, and precise control over film growth, PLD has become the predominant technique in the industrial-scale production of HTS.^29^^,^^40^ The process allows for excellent reproducibility and control over microstructure, offering the critical advantage of achieving high supersaturation conditions. This, in turn, promotes rapid nucleation and grain formation. Moreover, PLD is highly adaptable, capable of fabricating metastable phases and a broad spectrum of architectures—from simple thin films to more complex multilayered or nanostructured designs. It also facilitates the incorporation of various APCs, including zero-dimensional (0D) point defects and one-dimensional (1D) nanorods, which are essential for enhancing the critical current density across different operating regimes.^2^^,^^31^^,^^38^ As such, PLD is often regarded as the most straightforward method for defect engineering, with deposition parameters such as temperature and rate easily adjustable to reproducibly introduce a diverse array of pinning structures.^31^ Notably, REBCO conductors fabricated via PLD typically exhibit superior performance across a wide range of temperatures and magnetic fields when compared to those produced by alternative deposition techniques, making PLD highly favorable for industrial applications.^2^^,^^30^

However, PLD is not without limitations. Challenges such as the handling of materials with volatile components, maintaining uniform film thickness over large areas, and precise defect control remain significant concerns.^29^^,^^41^^,^^42^ Consequently, ongoing research is focused on addressing these issues to optimize the deposition process and develop next-generation HTS conductors with improved performance. Table 1 (^2^^,^^18^^,^^19^^,^^20^^,^^21^^,^^22^^,^^23^^,^^24^^,^^25^^,^^26^^,^^43^^,^^44^^,^^45^^,^^46^^,^^47^^,^^48^^,^^49^^,^^50^^,^^51^^,^^52^^,^^53^^,^^54^^,^^55^^,^^56^^,^^57^^,^^58^^,^^59^^,^^60^^,^^61^^,^^62^^,^^63^^,^^64^^,^^65^^,^^66^^,^^67^^,^^68^) summarizes and compares PLD with some other main deposition techniques in terms of cost, scalability, and performance to contextualize PLD’s advantages and limitations.

**Table 1:** Comparative analysis of REBCO deposition techniques

Technique	Cost	Scalability	Film Performance	Key Advantages	Key Limitations
PLD	Medium to High	Medium to High	High	Stoichiometric transfer, relative simplicity, versatility, reproducibility, and high film quality at reasonable rates.	Complex setup and modifications are required for scalability and throughput increase, and high equipment cost.
	High capital and operating cost (vacuum + high-power laser). Recent multi-beam systems and other modifications improve throughput, but capital cost remains substantial.	Moderate to high industrial scale. Small-area deposition by default; can scale to long tapes with moving substrate, multi-beam setups, and other modifications. Throughput is moderate to high (standard rates ≈0.1–0.2 μm/min and higher), industrial rates could reach 0.3–0.6 μm/min and higher, still some challenges to increase further production rate (throughput and yield) of kilometer-length films.	Excellent epitaxial film quality (dense, highly crystalline). High Tc and Jc are achievable with good growth control. Performance can be enhanced via doping and the creation of nanoscale pinning defects that boost Jc. Films have high phase purity, smooth morphology, good conformality, and uniformity.		
CVD/MOCVD	Medium to High	Medium to High	Medium to High	Good scalability and throughput, uniform, large-area coverage.	High precursor and process cost, complex chemistry control, and high-temperature requirements.
	High-cost equipment (reactor, gas delivery) and precursors. Operating cost is high but can coat a large area, amortizing cost. High throughput reduces per-meter cost, making it economically attractive at scale.	Highly scalable for continuous processing. Reel-to-reel MOCVD proven for long tapes. Can coat both sides of a substrate and wide tapes with uniform thickness. Moderate to high deposition rates (0.1–0.2 μm/min or more) enable industrial throughput.	Good- to High-quality epitaxial films, competitive with PLD, though Jc is often lower due to less sharp grain boundaries. Slightly more secondary inclusions can appear if process control falters. Moderate growth control. Can incorporate pinning nanostructures (e.g., BaZrO_3_) for higher performance.		
Sputtering	Medium	Medium	Medium	Relatively straightforward and reliable process, compatible with standard semiconductor fabrication infrastructure, which means established equipment and processes can be leveraged (useful for making multilayer devices with YBCO).	Stoichiometry control issues, which can be improved with several measures that often reduce the effective deposition rate or add complexity, are relatively more prone to defect formation, and achieving high Jc films requires significant process complexity (careful tuning of plasma conditions, very high substrate temperatures, additional annealing).
	Vacuum-based but no expensive laser; uses magnetron guns (widely available). Targets (e.g., YBCO ceramic) are pricey and require periodic replacement. Overall, relatively affordable for labs and semi-industrial use, but the cost is not low due to relatively long deposition times.	Employed for short samples and buffers. Some reel-to-reel trials, but deposition rate is modest (typically ∼0.01–0.05 μm/min), so coating long lengths is time-consuming. Uniform and conformal coating of wide tapes is difficult (edge effects). No current mass production of REBCO by sputtering, only niche or experimental scaling (≪100 m).	Moderate quality with relatively lower Jc on average. Moderate growth control. Achieving uniform phase and oxygenation is challenging over long lengths. Pinning structures must be introduced by complex co-sputtering or post-processing; thus, in-field Jc improvement lags behind PLD/MOCVD. While epitaxial quality can be excellent locally, maintaining that over a long, moving substrate is tougher.		
MBE	Very High	Low	High	Atomic-level control - thickness, interfaces, composition, high precision, *in situ* monitoring, and flexibility is possible with RHEED (reflection high-energy electron diffraction).	Extreme cost and low throughput, technical difficulties (source oxidation, narrow growth windows, and oxygen delivery challenges).
	Very high cost due to Ultra High-Vacuum system, multiple effusion cells. Expensive to maintain and very slow throughput (high cost per film). Typically, only in specialized labs.	Poor scalability. Small substrate sizes (1–2 inches) and very slow growth (rates typically below ∼0.1 nm/min). Not practical for long lengths or volume production. Precision-oriented, not throughput-oriented.	Superb film crystallinity and interface control (atomic layer precision). Tc and Jc of films can equal or even exceed others in ideal cases (due to purity and perfect epitaxy).		
ALD	High	Low	High	Precise thickness and composition control, unmatched uniformity and conformality even for 3D geometries.	Slow deposition rate and scale challenges, careful sequence control required for complex stoichiometry like REBCO, post-processing needs.
	Equipment and precursors are expensive, and deposition is slow, increasing the cost per thickness. Still cheaper than MBE for comparable control.	Good scalability in area and surface complexity. Can uniformly coat large or complex 3D surfaces (conformal coverage unmatched by other methods). Throughput (rates typically below ∼nm/min) is low for thick films, but newer spatial ALD techniques can improve speed.	High atomic level control for conformality and thickness, but use for complex REBCO films is still in development. Film crystallinity can be improved by post-deposition annealing. Overall film quality is high (smooth, uniform, conformal).		
Solution Deposition	Low	Medium	Low to Medium	Low-cost, non-vacuum process, good scalability potential, high material utilization, and easy composition tuning.	Multi-step processing complexity, films are more prone to defects, and lower density.
	Very low-cost equipment (no vacuum; uses solution processing and furnaces). Precursors (metal salts) are relatively inexpensive. The process is simple and cheap per unit area, making it attractive for low-cost HTS wire.	Highly scalable in length – e.g., continuous dip-coating or slot-die coating on 100 m+ tapes. Limited by furnace size and time; batch processing of multiple tapes in one furnace helps throughput. Typically requires multiple passes for thick films.	Relatively poor growth control, conformality, and uniformity of films. Films may have more porosity or larger second-phase particles. Lower supersaturation growth leads to larger, less dense pinning defects, which can reduce Jc under high fields. Requires careful and extensive heat treatment to avoid misoriented grains.		

## YBCO

Historically, PLD has been most prominently utilized in the fabrication of HTS thin films. The technique has been particularly effective in producing high-quality films of cuprate-based superconductors, notably the yttrium barium copper oxide (Y-Ba-Cu-O, or YBCO) family. These films exhibit superconducting onset temperatures around 95 K and reach zero electrical resistance at approximately 85 K when deposited on SrTiO_3_ (STO) substrates.^28^^,^^41^^,^^69^^,^^70^

YBCO remains a benchmark material in HTS research and applications due to its favorable properties, including high critical current densities in the range of 1–4 MA/cm^2^, relatively elevated critical temperatures, and robust flux pinning capabilities.^31^^,^^71^ These attributes make YBCO thin films fabricated via PLD particularly well-suited for various high-performance superconducting technologies.

**Figure 4:**
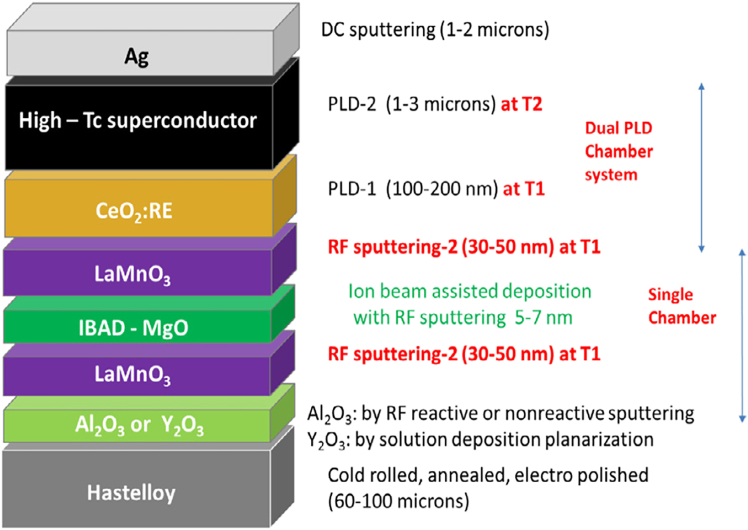
Example of the 2G HTS tape architecture Reproduced from^72^ with the permission from IOP.

Figure 4 presents an example of a second-generation (2G) coated conductor architecture, composed of a multilayered stack engineered for high superconducting performance, mechanical robustness, and chemical compatibility.^72^ The base is a Hastelloy substrate, chosen for its mechanical strength, corrosion resistance, and flexibility. On top of it, usually an Al_2_O_3_ or Y_2_O_3_ planarization layer is applied to smooth the substrate surface and prevent diffusion of unwanted elements, like Ni or Cr. An IBAD-MgO layer imparts the necessary biaxial texture to the stack—essential for aligning the crystalline axes of the subsequent layers. A LaMnO_3_ buffer layer is deposited to promote epitaxial growth and act as a chemical barrier. Above the buffer stack, a CeO_2_ layer is typically deposited to minimize lattice mismatch, improve wetting, and nucleation for such superconducting layers as YBCO. Then, after the high-Tc superconducting layer, follows a silver (Ag) cap layer to protect the superconductor from environmental degradation and facilitate current transfer during operation. To summarize, the buffer layers serve as crucial intermediaries that transfer the biaxial texture from the substrate to the superconducting layer, ensuring a barrier from unwanted diffusion, epitaxial growth, and minimizing lattice mismatch, while the superconducting layer provides the high critical current and magnetic field performance essential for advanced superconducting applications.

PLD has been extensively utilized to fabricate high-quality YBCO films, which are widely implemented in superconducting wires and coated conductors. The optimization of PLD parameters—such as laser fluence, substrate temperature, ambient oxygen pressure, and other growth conditions—has proven essential for tailoring the superconducting properties of these films.^30^^,^^73^ Notably, fine-tuning the deposition rate and energy density can significantly reduce processing time—by up to a factor of five—while maintaining film quality within acceptable limits, contributing to more cost-effective production methods.^74^

### Atmospheric influence in PLD-grown YBCO films

Investigations into the PLD growth of YBCO films under different atmospheric conditions—including oxygen, nitrous oxide (N_2_O), and nitrogen—have demonstrated that YBCO phase formation at high temperatures can occur even in the absence of oxygen, with superconducting behavior preserved across all environments tested.^75^ This oxygen-independent synthesis is particularly advantageous for applications involving oxidation-sensitive substrates.

Further studies on PLD growth in mixed O_2_/Ar atmospheres revealed a strong influence of argon content on the surface morphology of YBCO films.^76^ An increased argon fraction led to reduced surface particle density and larger particle sizes, while the lattice parameters (1.168 nm) and critical temperature (Tc ≈ 90 K) remained consistent. These results were attributed to a uniform post-deposition annealing treatment in pure oxygen applied to all samples. Additionally, advanced heterostructures such as YBCO films enhanced with LaAlO_3_ (LAO) APCs have been successfully synthesized using a combination of PLD and magnetron sputtering at a 25:75 O_2_/Ar ratio. These composite films exhibited improved superconducting performance, notably higher critical current densities (Jc) under magnetic fields exceeding 1 T, highlighting the potential of this approach for high-field applications.^76^

### Impact of deposition temperature on superconducting properties and microstructure

**Figure 5:**
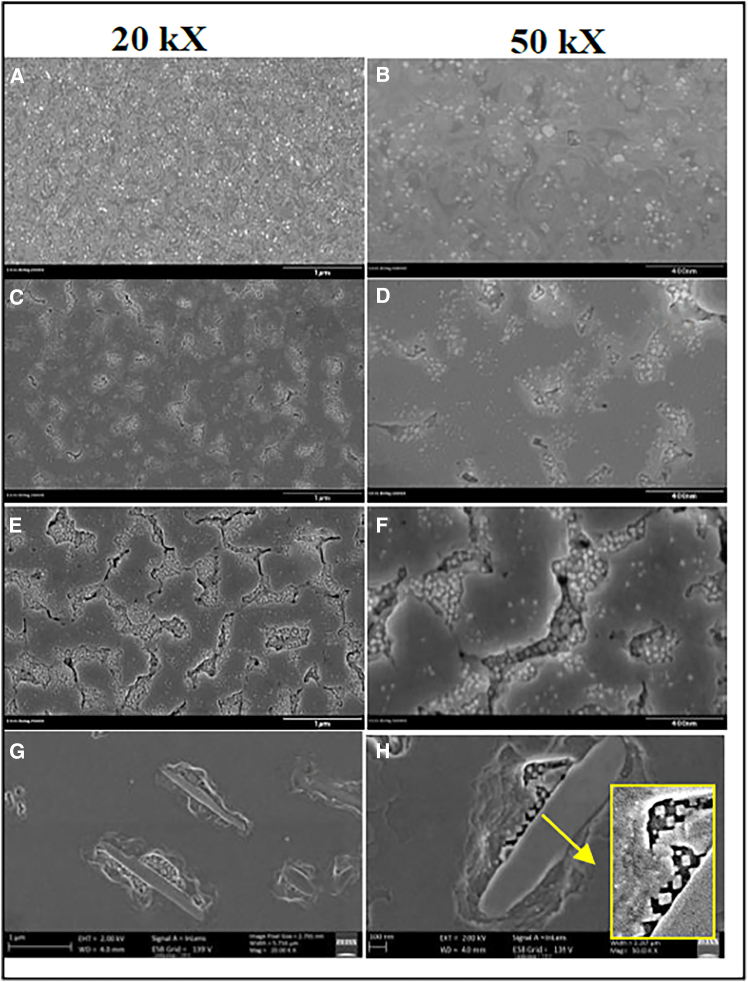
SEM images of pristine and doped YBCO (A and B) pristine YBCO, YBCO doped with (C–F) BZO+Y2O3 and (G and H) BZO at different deposition temperatures (A–D) 810°C and (E–H) 825°C.

The performance of superconducting materials is significantly influenced by their deposition temperature, which plays a crucial role in determining their characteristics. For instance, Sebastian et al.^77^ investigated the critical current density and flux pinning landscape of BaHfO_3_ (BHO) and Y_2_O_3_-doped YBCO thin films across a temperature range of 790°C–825°C. Their findings indicated that the optimal growth temperature for achieving robust, isotropic pinning and the highest critical current density was 810°C. Initially, it was hypothesized that the temperature effect was due to increased ad-atom mobility and diffusion during the film growth process. However, transmission electron microscopy (TEM) analysis did not support this interpretation. Moreover, varying the deposition temperature (810°C and 825°C) produced distinct effects depending on the specific doping combinations of BaZrO_3_ (BZO) and Y2O3. Notably, the YBCO thin film doped with 2 vol. % BZO and 3 vol. % Y_2_O_3_, deposited at the optimal temperature of 825°C, demonstrated the highest critical current density at both low (5 K) and high (65 K) temperatures across a broad magnetic field range (0–9 T).^78^ These results were attributed to the synergistic effects of the optimized doping levels and the favorable PLD deposition conditions, which facilitated the creation of a microstructure with an increased density of defects and pores, promoting more isotropic behavior. For example, SEM images on Figure 5 clearly show the distinguishing influence of the doping on the film morphology, where doped surfaces had significantly more defects, pores, and grains.

In a related study, researchers explored the effects of varying CeO_2_ doping concentrations in YBCO thin films and identified an optimal deposition temperature for maximizing the critical current (Ic).^79^ This temperature was found to be dependent on the doping level: films doped with 1 and 2 mol.% CeO_2_ exhibited optimal performance at a higher deposition temperature (830°C), whereas those with 3 mol.% CeO_2_ achieved peak performance at a slightly lower temperature (820°C). For films with lower doping levels (1–2 mol.%), the improvement in Ic with increasing temperature was attributed to the suppression of large grain formation and a reduction in grain misorientation. In contrast, the lower optimal temperature for the 3 mol.% CeO_2_-doped films was ascribed to the substitution of Y atoms by Ce, which introduced lattice distortions.

To further elucidate the influence of deposition temperature on the microstructure and superconducting properties, researchers investigated the relationship between stacking fault (SF) density and critical current density (Jc).^80^ An increase in deposition temperature was found to correlate with a higher SF density, which in turn led to a significant enhancement in Jc under applied magnetic fields. TEM analyses in one of the aforementioned studies [45], confirmed a linear correlation between SF density and Jc, supporting the hypothesis that stacking faults act as effective flux pinning centers and thus contribute to the improved superconducting performance of YBCO films.

Several research groups have further focused on precise substrate temperature control during the PLD process. For instance, Liu et al. introduced a seed layer technique, wherein a thin layer of 5 mol.% BHO-doped YGBCO was initially deposited at substrate temperatures ranging from 710°C to 820°C, followed by a thicker top layer deposited at 820°C.^81^ The optimal substrate temperature for the seed layer was identified as 790°C, at which the resulting YGBCO+BHO films exhibited a maximum Jc of 4.0 MA/cm^2^ at 77 K in self-field conditions. The crystallinity and texture of the films improved with increasing seed layer temperature up to 790°C, beyond which film quality and superconducting properties began to deteriorate. This emphasizes the critical importance of precise temperature control during the seed layer deposition.

**Figure 6:**
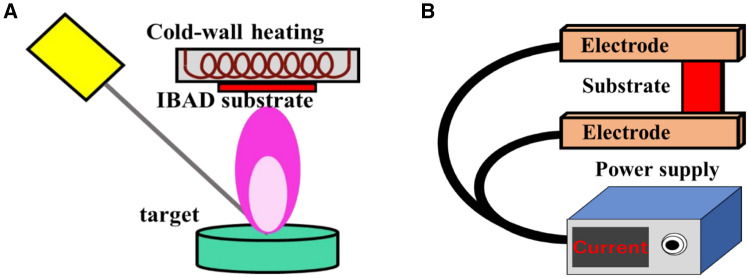
Schematic drawings of substrate heating (A) Cold-wall technique: the substrate is heated. (B) Self-heating technique: the substrate is heated by Joule heat generated by the resistance of the substrate.

Further insights into the impact of substrate temperature variation were provided by Sato et al.,^82^ who addressed the formation of undesired a-axis grains during the deposition of thick films—a phenomenon exacerbated by reduced substrate surface temperatures, which leads to a decline in critical current. By employing a self-heating method, wherein the metal substrate is heated via Joule heating (Figure 6), rapid thermal adjustments could be made during deposition. This approach effectively suppressed the formation of a-axis oriented grains, even in thicker films, thereby preserving high Jc values. The ability to dynamically control substrate temperature during growth proved essential for optimizing the structural and superconducting properties of the films.

### Influence of target density, composition, and geometry

The condition of the PLD target plays a critical role in the development of new materials and their modifications, and must always be taken into careful consideration. For example, during the investigation of the influence of target density on the deposition of Y_0_._5_Gd_0_._5_Ba_2_Cu_3_O_7_−δ (commonly denoted as YGBCO, representing Y_x_Gd_1-x_Ba_2_Cu_3_O_7_−δ), it was observed that the superconducting film properties were significantly affected by the density of the ceramic target.^83^ A target with lower density (4 g/cm^3^) produced a greater number of lower-energy ablated species, resulting in the formation of a more uniform and compact film morphology. In contrast, a higher-density target (5.5 g/cm^3^) emitted fewer ablated species, promoting less uniform nucleation and leading to a more porous film structure. Consequently, the film deposited using the lower-density target exhibited superior superconducting characteristics, with a critical temperature of 91.3 K and a critical current density of 5.4 MA per square centimeter at 77 K. In a similar study involving YBa_2_Cu_3_O_7_−δ (YBCO),^84^ nanostructured targets with varying grain sizes (25–45 nm) and densities were synthesized via a sol-gel route. It was found that increasing the target density led to a reduction in surface roughness of the deposited films, although a minor decrease in critical current density was also recorded. Overall, the influence of target density on the superconducting performance of YBCO was determined to be marginal.

**Figure 7:**
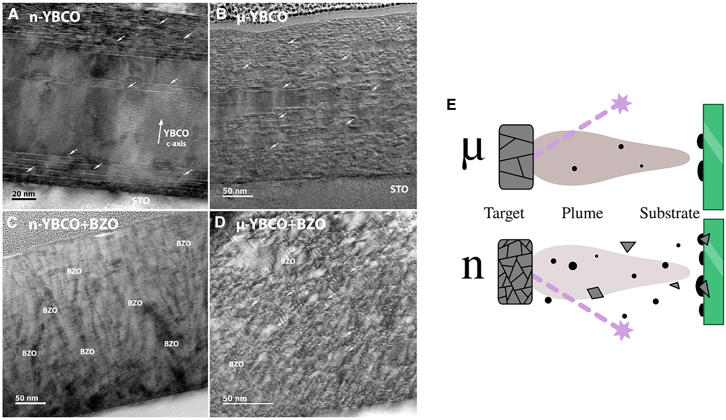
TEM images of undoped and BZO-doped YBCO (A–D) YBCO with different target grain sizes, showing stacking faults and BZO nanostructures; (E) schematic of the target grain size effect on film growth.

In fact, the exact impact of target density has not been extensively researched yet to come up to specific conclusions; nevertheless, the available studies at the moment indicate that this impact could also vary based on the material target. For example, in more recent research, a group of researchers demonstrated that the microstructure of PLD targets—specifically the combination of grain size, packing density, and 4 wt % BaZrO_3_ (BZO) content—dictated the plume chemistry, defect topology, and hence the vortex-pinning anisotropy of REBCO films.^85^ Densely compacted nanocrystalline YBCO (nm-grains, 75%–86% relative density) ejects multi-atom clusters that seed many small growth islands, forming a tight grid of threading dislocations and long stacking faults; in this matrix, BZO self-organizes into vertically continuous nanorods, delivering a strong c-axis critical current peak sustained to 8 T (Figure 7). By contrast, coarser, less-dense microcrystalline targets (μm-grains, 76%–80% density) ablate mainly atomic species, yield sparse defects, and break the same BZO load into shorter, more splayed rods that flatten the critical current profile and favor low-field, angle-independent performance (Figure 7).

A subsequent study by the same group extended this “target-engineering” concept to industrial IBAD-MgO templates and showed that merely shrinking the YBCO-BZO target grains to the nanometer scale—without altering BZO loading or laser settings—suppressed the substrate-film grain-boundary transfer altogether.^86^ Moreover, it accelerated growth by 25% by ejecting multi-atom clusters instead of single ions, and thus delivering more material to the substrate per pulse. It also drove the self-assembly of 7–8 nm-diameter BZO nanorods that were three times longer and twice as splayed as those obtained from conventional microcrystalline targets. The resulting films exhibited a 40% boost in c-axis critical current at 77 K and maintained over an order-of-magnitude advantage above 2 T, while leaving ab-plane transport unchanged, confirming that vertically coherent nanorods—not grain boundaries—now dominate the pinning landscape. A simple kinetics model linked this behavior to cluster-rich plumes: once emitted particle radii exceeded ∼12 nm, the lateral force exerted by substrate grain boundaries became negligible, allowing defect-free overgrowth and a denser dislocation-fault grid that further guided rod alignment. Collectively, these findings reinforce that target grain refinement, alongside compaction and BZO dosing, could be a decisive lever for scaling high-field REBCO coated conductors.

Further investigation into the effects of target composition and its transfer to the film was carried out by the research group led by Mozhaev et al.^87^ Their study focused on minimizing compositional deviations between the PLD target and the resulting film. This was achieved by implementing laser beam scanning across the target surface and substituting oxygen with argon in the deposition environment. The laser scanning technique allowed for a more uniform distribution of the ablation plume and mitigated spatial variations in laser spot energy density. These adjustments reduced both angular and inelastic scattering of the ablated species, leading to the formation of dense YBCO films with highly stable superconducting properties. Additionally, the targets were subjected to periodic resurfacing and repolishing to ensure consistent material transfer and compositional fidelity between the target and the deposited film. The study also revealed that an increased concentration of yttrium in the target resulted in the formation of porous, rough-surfaced films, whereas the formation of a quasi-liquid layer based on barium-copper oxides during deposition facilitated improved growth of high-density YBCO films. Techniques involving similar transient liquid-phase mechanisms, such as transient liquid-assisted growth (TLAG), will be discussed in more detail later.

In another investigation involving barium zirconate (BZO)-doped YBCO films,^88^ both single-target systems (with the general composition 123_1-x_BZO_x_) and multilayer targets (denoted as (BZO_n_/123_m_)_N_) were studied to assess their influence on superconducting properties. For multilayer deposition, the PLD system was modified to include automated target rotation and pulse sequencing, with precisely defined deposition rates for each layer. The multilayer films demonstrated minimal degradation in critical temperature and self-field critical current density, even at high BZO doping levels (up to 10%). In contrast, films grown from single-target systems showed a linear decline in these properties as BZO concentration increased. The superior performance of the multilayer films at elevated BZO concentrations was attributed to reduced zirconium diffusion and minimal chemical incompatibility effects, underscoring the effectiveness of the multilayer strategy in preserving and enhancing superconducting performance.

Furthermore, the significance of the geometric configuration between the target and substrate was examined by Backen et al.,^89^ who reported that an off-axis PLD setup—where the target and substrate are positioned perpendicularly—can achieve comparable reproducibility and uniformity in film deposition. This configuration is particularly important for ensuring consistent superconducting properties across large-area films. Additionally, a quasi-multilayer deposition approach involving alternating layers of YBCO and metallic inclusions (such as hafnium) was shown to enhance flux pinning efficiency, further contributing to the improvement of superconducting characteristics.

### Optimizing multiple PLD parameters

In the majority of studies, a more comprehensive strategy has been adopted, involving the simultaneous evaluation of multiple PLD parameters. For instance, Xiong and colleagues systematically optimized deposition conditions by enhancing target quality and modifying the oxygen ambient pressure to improve the film’s performance in microwave applications.^90^ Specifically, the use of a high-density target contributed to superior surface morphology, while increasing the oxygen pressure and laser pulse energy proved essential for reducing microwave surface resistance and enhancing the critical current density. These modifications facilitated microstructural improvements under elevated energy and pressure conditions, which were pivotal for achieving the desired microwave properties. Although the study did not delve into an in-depth structural analysis, this optimized approach enabled the successful deposition of large-area, double-sided, and uniform films. Notably, microwave filters fabricated using these improved films were directly compared with those made from commercial counterparts, and the results affirmed that the produced films fully satisfied the stringent performance requirements of planar microwave devices, underscoring their potential for practical implementation.

In another study, Xu et al.^91^ thoroughly examined the effects of various deposition parameters, such as substrate inclination angle and film thickness, on the crystallinity of the deposited films. The results indicated that a film thickness of 500 nm combined with a 15° substrate tilt yielded optimal crystallinity, characterized by a dense microstructure and minimal porosity. While the study did not extend to a full superconducting performance evaluation, the improved morphology is expected to correlate with enhanced superconducting properties, warranting further exploration. Favre and co-authors conducted a detailed study focused on substrate temperature, deposition time, and target-substrate distance, aiming to modulate the residual strain within the films.^92^ Their results demonstrated a nearly linear suppression of the critical temperature with increasing c-axis lattice expansion, attributed to in-plane compressive strain. This phenomenon was explained through a theoretical model involving quantum phase fluctuations and reduced Coulomb screening along the c-axis. According to the model, elongation of the c-axis weakened electrostatic coupling between adjacent CuO_2_ planes, thereby decreasing the critical temperature. The optimal deposition conditions that minimized residual strain and maximized the critical temperature (89 K) were identified as a substrate temperature of 690°C, a deposition duration of 30 min, and a target-substrate separation of 2 cm.

Further investigations into the influence of deposition temperature and growth rate on the formation of columnar and nanoparticle defects revealed that the optimal temperature range for achieving an effective defect landscape in doped films was between 785°C and 800°C.^93^ This temperature window supported a balanced distribution of columnar defects aligned along the c-axis and randomly oriented nanoparticles, both essential for strong and isotropic flux pinning. Temperatures below this range favored the formation of isolated nanoparticles, while higher temperatures led to predominantly columnar structures, each less effective for comprehensive pinning. Additionally, a laser repetition rate of 2 Hz was found to be optimal, as it provided sufficient temporal spacing between pulses, allowing dopant species increased surface mobility and facilitating the growth of well-aligned, extended columnar defects. This combination of deposition parameters led to significantly enhanced critical current densities, reaching up to 3 MA/cm^2^ at 77 K in self-field, by simultaneously promoting strong vortex pinning and minimizing flux creep. As a result, films exhibited high in-field critical currents with nearly isotropic angular dependence, making them suitable for demanding superconducting applications.

### Elemental doping and nanoengineering strategies for enhancing flux pinning

Initial efforts to enhance the performance of cuprate films often centered on conventional elemental doping strategies aimed at improving processing outcomes and flux pinning capabilities. One early study investigated varying concentrations of terbium and cerium dopants.^94^ It was observed that terbium incorporation preserved a high critical temperature while enhancing the critical current density, with Y_0_._99_Tb_0_._01_ compositions demonstrating superior performance across a broader range of magnetic field and temperature conditions. In contrast, cerium doping resulted in a marked suppression of both critical temperature and current density as its concentration increased, suggesting a deleterious impact on superconducting behavior. These findings underscored that while small amounts of terbium can positively influence flux pinning, cerium exhibits limited utility due to its adverse effect on superconductive properties.

Contemporary research has shifted toward advanced nanoengineering approaches, particularly through the introduction of APCs within the film matrix to significantly boost current-carrying capacity—an essential criterion for technological applications.^1^^,^^43^^,^^95^ Various studies have examined the incorporation and optimization of such pinning centers in films fabricated via PLD. For example, Kuroki et al. emphasized the importance of microscale heterogeneities arising from Ba_2_YbNbO_6_-based doping, highlighting their critical role in the development of high-performance coated superconductors.^96^ In parallel, Chen and colleagues explored the comparative effects of nanorod- and nanoparticle-based APCs, specifically BZO nanorods and Y_2_O_3_ nanoparticles.^97^ Their findings, in agreement with earlier work by Maiorov and others,^93^^,^^97^ revealed that the inclusion of Y_2_O_3_ nanoparticles led to an increased volume fraction of isotropically distributed BZO pinning centers. This significantly enhanced pinning force density and contributed to an elevated critical temperature relative to films doped exclusively with nanorods.

Additional investigations introduced alternating nanostructures of BZO and Y_2_O_3_ into multilayered films, fabricated via PLD.^98^ These engineered heterostructures yielded a marked improvement in flux pinning performance, achieving a critical current density 1.5 times higher than that of undoped reference films (2.66 MA/cm^2^, self-field). Furthermore, these enhancements persisted under increasing magnetic fields, with a less pronounced decline in performance. Sueyoshi et al. conducted a comparative study on films doped with either BZO or BaSnO_3_ nanorods, focusing on their influence under varying magnetic field strengths.^99^ The results demonstrated that films doped with 1.5 wt % BZO nanorods exhibited the highest critical current density and most effective flux pinning, outperforming BaSnO_3_-doped counterparts due to superior nanorod characteristics and pinning efficacy.

Comprehensive reviews by Jha and Matsumoto further catalog recent advancements in APCs integration across a range of rare-earth cuprate superconductors^31^ These studies collectively highlight the significant progress achieved through precision nanoengineering in elevating the current density and overall performance of superconducting films.

### Optimizing substrate, buffer layer, and interface engineering

Numerous studies have focused on optimizing substrates, buffer layers, and their interfaces with superconducting films to enhance the performance of HTS. A recent investigation demonstrated that YBCO films synthesized via PLD on MgO substrates could retain superconducting properties at thicknesses as low as 3 nm, thereby extending the frontier of superconductivity in ultrathin materials.^100^ In addition to ultrathin film fabrication, the study also reported the successful realization of YBCO nanowires through a combination of PLD growth and subsequent lithographic patterning and etching processes.^101^^,^^102^ These nanoscale structures are of particular relevance for the development of superconducting nanoelectronics. Notably, the observation of a voltage switching effect in 10-nm-thick nanowires without Au capping was a critical finding.^100^ The bistability, attributed to localized normal-state domains, indicates a promising mechanism for controlling superconducting states in applications such as single-photon detectors.

Trabaldo et al. further elaborated on the use of YBCO nanowires in superconducting quantum interference devices (SQUIDs) and magnetometers, emphasizing their potential in quantum sensing applications.^103^ In a related study, the structural and electrical transport characteristics of 50 nm-thick PLD-grown YBCO films with various doping levels were investigated.^104^ It was shown that films with lower doping levels were predominantly untwinned—exhibiting a reduced density of twin boundaries, which are known to influence superconducting behavior. Interestingly, these untwinned films experienced a similar strain state to slightly overdoped counterparts. The study also examined the effect of strain on superconducting properties and established the doping dependence of the upper critical field. These findings underscore the capability of PLD to produce nm-scale films with tunable strain, enabling detailed studies of strain-induced effects on superconductivity.

In contrast, Zhou et al. addressed performance optimization in thicker YBCO films.^105^ Their work revisited earlier studies involving BZO and Y_2_O_3_-doped YBCO,^93^ investigating the dependence of Jc on film thickness. The incorporation of APCs, specifically BZO and Y_2_O_3_, was found to mitigate the typical decline in Jc observed with increasing thickness. Impressively, the films maintained a high Jc of 2.3 MA/cm^2^ even at a thickness of 6.4 μm. This improvement was attributed to the effective pinning landscape introduced by the dopants, which preserved the film microstructure and enhanced overall superconducting performance across a broad thickness range.

The study by Stepantsov et al. demonstrated the successful PLD of YBCO films on SrTiO_3_ substrates, where the CuO_2_ planes were tilted relative to the surface by angles ranging from 0° (parallel orientation) to 70°.^106^ This tilt was introduced by rotating the crystalline lattice of the STO substrate around its [100] axis, which lies parallel to the film surface. The results showed that up to a tilt angle of 41°, the YBCO films maintained a high-quality single-crystal structure. However, beyond this critical angle, the films developed a two-domain texture accompanied by a significant increase in surface roughness. Such structural transformations are important to consider, as they can adversely affect superconducting performance, particularly in device applications where crystallographic uniformity is essential.

In related work, the importance of interface coherency between YBCO films and substrates or embedded pinning centers has been emphasized.^107^^,^^108^ One study successfully addressed the large lattice mismatch between YBCO films and BaZrO_3_ (BZO) nanorods—strong one-dimensional APCs (1D-APCs)—by introducing Ca/Cu substitution in the single CuO_2_ planes. This approach effectively expanded the YBCO c-axis at the interface, improving structural compatibility. As a result, the films exhibited a 5-fold increase in critical current density (Jc), highlighting the crucial role of interface engineering in enhancing superconducting performance.

Another notable study reported the PLD growth of YBCO films with b-axis orientation on the surface of (100)-oriented SrLaGaO_4_ crystals, using a 60 nm PrBa_2_Cu_3_O_7_−_x_ buffer layer^109^ These films transitioned into a superconducting state at a temperature of 89 K. X-ray diffraction analysis confirmed the single-crystalline nature of the b-oriented films and the absence of domains with alternative crystallographic orientations. The lack of structural twins and the high uniformity of the film underscore its potential for device applications where directional anisotropy and structural precision are critical.

Furthermore, the orientation and surface morphology of yttria-stabilized zirconia (YSZ) buffer layers were found to be highly sensitive to PLD deposition parameters, particularly oxygen pressure and laser repetition rate.^110^ These parameters significantly influenced the growth conditions and, consequently, the quality of the overlying YBCO superconducting films. The study identified optimal deposition conditions—an oxygen pressure of 1 mTorr and a low laser repetition rate of 80 Hz—that yielded the smoothest and most defect-free YSZ surfaces. Under these conditions, the YBCO films grown atop the YSZ buffer layers exhibited enhanced superconducting properties, achieving a Jc exceeding 4 MA/cm^2^ at 77 K.

### Influence of substrate and layer thickness on YBCO film performance in PLD

Substrate thickness has been identified as a critical parameter influencing both the PLD process and the performance of YBCO superconducting tapes.^111^ It was shown that decreasing the substrate thickness can lead to a significant increase in critical current density (Jc), which is highly desirable for high-performance applications. However, this improvement requires careful optimization of the fabrication process, particularly the combination of alternating beam-assisted deposition and PLD. Among the tested samples, the tape with the thinnest substrate (50 μm) exhibited the best performance, achieving a Jc of 0.1 MA/cm^2^ under an ultra-high magnetic field of 18 T at 4.2 K. Furthermore, the authors proposed additional strategies to enhance current density, including independent regulation of the nucleation temperature through the initiation of lateral flows affecting PLD nucleation, and mitigation of rapid temperature fluctuations caused by ion bombardment from the laser plume.

To further address the limitations posed by thickness effects in YBCO films, one study demonstrated the effectiveness of a superconductor/interlayer/superconductor tri-layer structure.^112^ By introducing indium into a CeO_2_ interlayer, the critical current density (Jc) nearly doubled compared to a single-layer YGBCO film. The optimal interlayer thickness was found to be 40 nm, indicating that such interlayers can play a key role in facilitating current transport and reducing the detrimental effects of increased film thickness.

Similarly, the thickness of buffer layers was also found to be a determining factor in the structural and superconducting quality of YBCO films.^113^ An optimal thickness of 221 nm for the CeO_2_ buffer layer provided the best in-plane and out-of-plane texture, along with an exceptionally smooth surface exhibiting a root-mean-square (RMS) roughness of just 0.6 nm. YBCO films deposited on such optimized buffer layers achieved a high Jc of 4.6 MA/cm^2^ at 77 K in self-field conditions.

Porokhov et al. explored the use of a novel substrate—single crystalline quartz—for YBCO growth, motivated by the potential of amorphous substrates to reduce alternating current losses in superconducting tapes.^114^ Despite the significant lattice mismatch of approximately 22%, YBCO films grown via PLD exhibited moderate superconductivity with a critical temperature of 85 K and a Jc of 0.01 MA/cm^2^ at 65 K. The relatively low critical current was attributed to internal film stress, which was not visually evident in scanning electron microscopy (SEM) and atomic force microscopy (AFM) analyses.

In another study, various substrates and deposition conditions were evaluated to optimize YBCO films for microwave device applications.^115^ A key finding was the importance of thermal contact between the substrate and the sample holder during deposition. A comparison of attachment methods—silver paste versus mechanical mask—revealed that silver paste at an optimized deposition temperature of 750°C resulted in the highest Tc (90.5 K) for YBCO films on STO substrates. Several substrate materials were tested, including STO, MgO, (LaAlO_3_)_0_._3_(Sr_2_AlTaO_6_)_0_._7_ (LSAT), SrLaAlO_4_ (SLAO), and NdGaO_3_ (NGO), with a focus on minimizing substrate-induced microwave losses while maintaining high superconducting performance. Among these, MgO was selected for further microwave characterization due to its low dielectric loss, acceptable critical temperature (86.5 K), and superior current density in samples prepared using a mechanical mask, making it highly suitable for microwave applications.

### Advancements and modifications in PLD techniques

In addition to optimizing standard PLD parameters, more substantial modifications to the PLD setup have been introduced to further enhance the superconducting properties of YBCO films. For instance, Ichino et al. explored the use of a combinatorial PLD technique, optimizing key parameters such as oxygen pressure and target-to-substrate distance.^116^ Unlike the conventional KrF laser, this approach employed a Nd:Y_3_Al_5_O_12_ (Nd:YAG) laser, which is considered more environmentally friendly due to the absence of hazardous gases and offers cost advantages. The combinatorial method involved a patterned plate with a rectangular aperture that moved over the substrate in synchronization with both laser pulses and target switching. This configuration enabled the deposition of layers with a linear thickness gradient—from zero to several monolayers—of pure and doped YBCO. These layers were subsequently fused through solid-phase diffusion at elevated growth temperatures. This technique allowed for the fabrication of BSO-doped (3.6 wt %) YBCO films exhibiting excellent superconducting properties, including a critical temperature of 90 K and a critical current density of 2.5 MA/cm^2^ at 77 K in self-field—values comparable to those achieved using conventional PLD. Additionally, it was found that optimizing oxygen pressure and the target-to-substrate distance could improve material yield by up to 19.0%; however, the highest yield did not correspond to the highest critical current density.

Similarly, De Silva et al. applied Nd:YAG-PLD to synthesize (Y_1-x_Ho_x_)Ba_2_Cu_3_O_7-_z films, exploring the partial substitution of Y with Ho to introduce effective pinning centers without distorting the lattice, owing to the similar ionic radii of Ho^3+^ and Y^3+^.^117^ The study examined multiple compositions, optimizing parameters such as oxygen pressure, substrate temperature, target-substrate distance, energy density, and deposition time for each. The composition with x = 0.3 exhibited the best superconducting performance, achieving a Tc of 87 K and a Jc of up to 1.45 MA/cm^2^. The results confirmed the potential of Nd:YAG-based PLD for producing high-quality YBCO films in a cost-effective manner.

Building on these earlier Nd:YAG-based combinatorial studies, Chaluvadi et al. have recently demonstrated that the first-harmonic (1064 nm) Nd:YAG laser alone can reproducibly deposit high-quality, c-axis-oriented YBCO and related oxide heterostructures without recourse to UV harmonics or excimer sources.^118^ By operating at a larger target-to-substrate distance (≈8–10 cm) and masking the beam to moderate fluence, the authors achieved smooth, droplet-free epitaxial YBCO films on LaAlO_3_ that reached Tc (onset) = 93 K after oxygenation and retained coherent superconductivity (zero resistance at 80 K) even in tri-layer YBCO/CeO_2_/LaNiO_3_ stacks. Electron microscopy also confirmed atomically sharp interfaces and stoichiometric transfer. Thus, the compact, gas-free Nd:YAG platform offered a cost-effective, environmentally benign alternative for PLD of REBCO tapes and oxide electronics while preserving superconducting performance.

**Figure 8:**
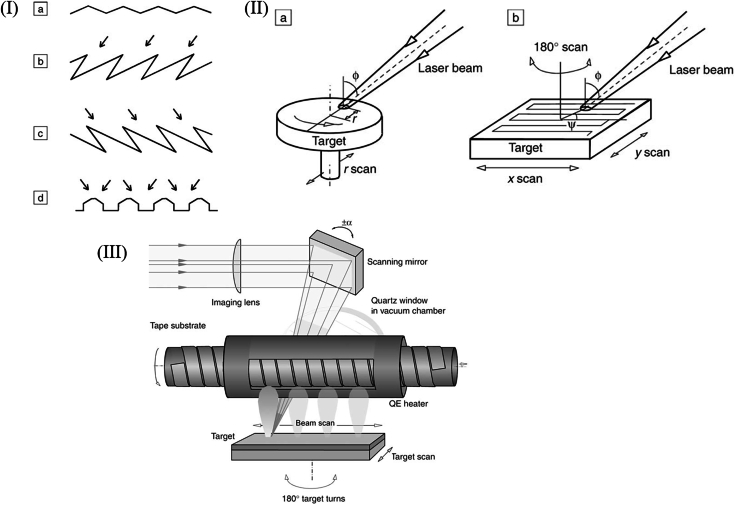
Schematics of target surface morphology and ablation (I) initial roughness and relief after laser ablation at different azimuth angles (a–d); (II) variable azimuth ablation modes: spiral (a) and meander (b); (III) high-rate PLD installation.

Further advancements to PLD have included the development of variable azimuth ablation (VAA) and quasi-equilibrium heating (QEH) techniques, which improved deposition efficiency, stability, and rate.^119^^,^^120^ VAA involved periodically altering the azimuthal angle between the laser beam and the target, thereby reducing surface roughening and enhancing deposition stability. A smoother target surface minimizes energy dissipation and maintains a higher deposition rate (Figure 8, top). Meanwhile, the QEH method maintained a nearly constant substrate temperature—with variations kept below 2°C—by using a tubular heater, a rotating IR-reflective screen (chopper), and a semi-enclosed chamber to reduce IR losses (Figure 8, bottom). This setup ensured consistent heating conditions, independent of the thermal contact between the substrate tape and the rotating drum. As a result, film microstructure and superconducting properties were significantly improved. The combined implementation of these enhancements in the high rate-PLD approach, along with optimized deposition conditions, led to YBCO films with high critical current densities: 2–3 MA/cm^2^ for 1 μm-thick films and over 1.4 MA/cm^2^ for 3 μm-thick films.^119^^,^^120^ This method presents a promising route for the cost-effective production of long-length, high-performance superconducting tapes.

Later in 2015, Abraimov et al. also implemented the QEH approach in their study on enhancing the superconducting properties of REBCO tapes.^121^ Their work introduced a double-disorder strategy that combined both intrinsic and extrinsic defect engineering to improve flux pinning. Intrinsic disorder referred to local stoichiometric variations within the REBCO matrix, resulting in nanoscale defects that function as APCs. These could be induced by modulating the oxygen pressure during the PLD process, which alters the angular distribution of atoms in the plasma plume. This variation leads to localized non-stoichiometric regions in the HTS layer without affecting the overall film composition. In parallel, extrinsic disorder was introduced via the incorporation of secondary phase nanoparticles such as BHO or BZO into the superconductor matrix. In addition to QEH, the authors optimized other PLD parameters. One such modification was akin to VAA, involving ablation from various spots on the target to maintain high deposition rates. They also optimized oxygen pressure—not only to promote intrinsic defects but also to enhance oxidation at the interfacial atomic layers, which plays a crucial role in film quality. The combined effect of these improvements resulted in a significant enhancement of the critical current density, particularly under high magnetic fields. The 4 mm-wide YBCO tapes demonstrated excellent performance, with reported critical currents reaching 309 A at 31 T and 1200 A at 5 T (4.2 K).

**Figure 9:**
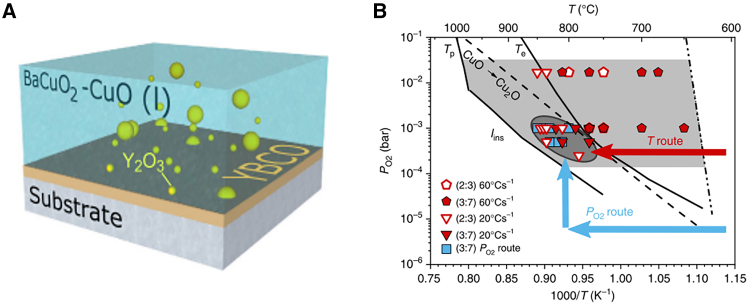
TLAG process (A) Schematic drawing of the process, and (B) T–PO_2_ phase diagram for YBCO TLAG growth.

Recent developments in PLD-based fabrication of cuprate superconductors also include the introduction of TLAG, a novel technique based on the temporary formation of a liquid phase to enable high-quality film growth.^122^ TLAG enables extremely rapid film growth rates—up to 1000 nm/s—while maintaining excellent superconducting properties.^123^^,^^124^ In this method, amorphous precursor films (e.g., BaCuO_2_, CuO, and Y_2_O_3_) are first deposited via PLD. These films are then heated above 800°C to induce a eutectic reaction, forming a transient Ba–Cu–O liquid phase with dispersed Y_2_O_3_ nanoparticles. A sudden increase in oxygen pressure subsequently initiates crystallization, forming the YBCO phase (Figure 9). Key process parameters—such as heating ramps, maximum temperature, and oxygen partial pressure—were carefully tuned to achieve YBCO films with critical temperatures up to 90 K. One notable advantage of TLAG-PLD is the prevention of BaCO_3_ formation, which is detrimental to YBCO crystallization. TLAG has demonstrated the potential for high-throughput synthesis of high-quality superconducting films with fewer secondary phases and reduced low-angle grain boundaries, both of which are essential for maximizing the critical current density.^122^

Another liquid-assisted processing (LAP) technique has recently been developed in conjunction with PLD, offering not only significantly faster growth rates—reaching up to 250 nm/min, comparable to TLAG—but also enhanced artificial flux pinning.^125^ This LAP approach utilized a non-stoichiometric target composition, which resulted in the formation of a minor liquid phase during deposition. The presence of this transient liquid phase facilitated enhanced diffusion of the deposited material within the growing film. Despite the high deposition rate, LAP-PLD successfully enabled the formation of mixed pinning landscapes, including both point-like and nanocolumnar defects. YBCO films synthesized with this method demonstrated critical current densities at 30–70 K comparable to those produced by conventional PLD. Remarkably, at lower temperatures, the performance was even superior—showing 5–10 times higher current densities. These advantages make LAP-PLD an appealing technique due to its potential for time and cost efficiency and relatively straightforward implementation.

A further PLD-based method incorporating liquid-phase dynamics is the vapor-liquid-solid (VLS) approach, which borrows aspects from liquid phase epitaxy (LPE).^126^ The VLS method involves three distinct stages, each defined by specific partial oxygen pressures, substrate temperatures, and laser frequencies (summarized in Table 2). Initially, a solid 200 nm YBCO layer is grown using a pure YBCO target. In the second step, a 50 nm Ba_3_Cu_7_O_10_ (BCO) liquid layer is formed on top by PLD using a 10 wt % Ag_2_O-doped BCO target. Finally, a third deposition stage is carried out using a 3 vol % BHO-doped YBCO sintered target, allowing growth through the liquid interface. All steps were performed continuously under vacuum, without interrupting the thermal cycle. This method enabled high-rate growth of BHO-doped YBCO films without sacrificing crystallinity. The improved performance was attributed to the three-dimensional molecular diffusion enabled by the transient liquid layer, which resulted in lower supersaturation at the growth front compared to conventional PLD. Moreover, APC-induced enhancements in superconducting properties were more pronounced in films fabricated using the VLS technique than in those grown by standard PLD.

**Table 2:** Summary on YBCO performance and PLD techniques and parameters used for deposition

Superconductor	Method	Conditions	Critical Temperature	Critical Current Density	Substrate	References
YBa_2_Cu_3_O_7−δ_ (YBCO)	PLD	Heater temperature: 760°C; Oxygen pressure: 0.7 mbar; Energy density 2 J/cm^2^	Thin films: 3 nm–15 K 5 nm–70 K 10 nm–78 K 20 nm–82 K 50 nm–85 K Nanowires: 10 nm–70 K 20 nm–75 K 30 nm–81 K	Nanowires 10, 20, 30 nm–50 MA/cm^2^ (4.2 K)	MgO	Arpaia et al.^100^
YBa_2_Cu_3_O_7−δ_ (YBCO)	PLD	UV KrF excimer laser; Frequency: 10 Hz Wavelength: λ = 248 nm; Substrate temperature: 600−750°С; Oxygen pressure: Р = 0.4 mbar; After deposition, the chamber was filled with pure oxygen to Р = 600 mbar, the substrate with the film was annealed at 550°C for 1 h.	89 K	NA	(100) SrLaGaO_4_ with 60 nm buffer layer of PrBa_2_Cu_3_O_7−x_	Stepantsov et al.^109^
YBa_2_Cu_3_O_7−δ_ (YBCO)	PLD	UV KrF excimer laser; Frequency: 10 Hz Wavelength: λ = 248 nm; Substrate temperature: 780°С; Oxygen pressure: Р = 0.7 mbar;	NA	NA	SrTiO_3_ (STO)	Stepantsov et al.^106^
YBa_2_Cu_3_O_7−δ_ (YBCO)	PLD	Oxygen pressure: 0.6 mbar; After deposition, samples were annealed at 800 mbar oxygen atmosphere for 60 min at 550°C.	∼85 K	10^4^ A/cm^2^ at 65 K	Buffer layers of 100 nm YSZ and 60 nm CeO_2_ on [001] SiO_2_ single crystal substrates	Porokhov et al.^114^
YBa_2_Cu_3_O_7−δ_ (YBCO)	PLD	Laser adjusted for ∼10^3^ pulses/cm^2^, 0.05 J/cm^2^ per pulse; Followed by oxygen post-loading performed at about 500°C^121^	NA	0.1 MA/cm^2^ (18 T, 4.2 K)	YSZ) buffer layer (2–3 μm); CrNi stainless steel (50 μm)	Usoskin et al.^111^
YBa_2_Cu_3_O_7−δ_ (YBCO)	PLD	Heater temperature: 760°C; Oxygen pressure: 0.7 mbar; Energy density: 1.5 J/cm^2^^127^^,^^128^	Overdoped (*p* = 0.17), a-axis: 14 T = 72 K 10 T = 75 K 6 T = 77 K 3 T = 82 K 1 T = 83 K 0 T = 85 K Underdoped (*p* = 0.12), a-axis: 14 T = 10 K 10 T = 20 K 6 T = 30 K 1 T = 47 K 0 T = 55 K	NA	(110) MgO	Wahlberg et al.^104^
YBa_2_Cu_3_O_7−δ_ (YBCO)	PLD	Pulse laser energy: 250 mJ; Laser frequency: 2 Hz; Target-substrate distance: 4.5 cm; The deposition temperature: 780°C; The deposition atmosphere has either oxygen, nitrous oxide or nitrogen; The deposition pressure: 20, 30, 40, 50 Pa; The laser sputtering time: 90 min; Some samples were cooled to the oxygen-annealing temperature and brought to vacuum below 10^−4^ Pa again, and then injected with oxygen (flowing atmosphere).	Oxygen: 20 Pa = 81 K 30 Pa = 92 K 40 Pa = 80 K 50 Pa = 75 K Nitrous oxide: 10 Pa = 70 K 20 Pa = 82 K 30 Pa = 87 K 40 Pa = 87 K 50 Pa = 88 K Nitrogen: 20 Pa = 82 K 30 Pa = 84 K 40 Pa = 89 K 50 Pa = 89 K Nitrous oxide: 350°C ox.-annealing T = 87 K 400°C ox.-annealing T = 89 K 450°C ox.-annealing T = 90 K Nitrogen: 350°C ox.-annealing T = 85 K 400°C ox.-annealing T = 88 K 450°C ox.-annealing T = 91 K	50 K and self-field Oxygen: 10 Pa–0.8 MA/cm^2^ 20 Pa–3.75 MA/cm^2^ 30 Pa–6.75 MA/cm^2^ 40 Pa–4.5 MA/cm^2^ 50 Pa–3.8 MA/cm^2^ Nitrous oxide: 10 Pa–0.75 MA/cm^2^ 20 Pa–1.5 MA/cm^2^ 30 Pa–3.75 MA/cm^2^ 40 Pa–4.3 MA/cm^2^ 50 Pa–5.3 MA/cm^2^ Nitrogen: 10 Pa–0 MA/cm^2^ 20 Pa–1.5 MA/cm^2^ 30 Pa–3 MA/cm^2^ 40 Pa–3.1 MA/cm^2^ 50 Pa–3.3 MA/cm^2^	(00L)-oriented SrTiO_3_ single crystal substrate	Guo et al.^75^
YBa_2_Cu_3_O_7−δ_ (YBCO) doped with 4 vol. % BHO and 3 vol. % Y_2_O_3_	PLD	KrF excimer laser (248 nm); Fluence: 1.6 J/cm^2^; Repetition frequency: 8.0 Hz; Growth temperature of 790°C–825°C; Oxygen partial pressure: 300 mTorr; All samples were further annealed at 500°C for 30 min approximately at one atmosphere oxygen pressure.	At 790°C = 86.66 K; At 810°C = 88.82 K; At 825°C = 89.31 K;	Self-field 65 K: At 810°C–3 MA/cm^2^; At 825°C–2 MA/cm^2^; At 790°C–0.6 MA/cm^2^; Self-field 50 K: At 810°C–5 MA/cm^2^; At 825°C–2.8 MA/cm^2^; At 790°C–2 MA/cm^2^; Self-field 5 K: At 810°C–20 MA/cm^2^; At 825°C–6.5 MA/cm^2^; At 790°C–4.5 MA/cm^2^;	(100) LaAlO_3_ (LAO) single crystal substrate	Sebastian et al.^77^
YBa_2_Cu_3_O_7−δ_ (YBCO) doped with CeO_2_	PLD	Oxygen atmosphere: 800°C–840°C; Then the samples were annealed at 500°C in flowing O2 gas for 5 h.	NA	77 K, self-field: 1 mol % CeO_2_ @ 830°C = 100 A; 2 mol % CeO_2_ @ 830°C = 75 A; 3 mol % CeO_2_ @ 820°C = 65 A;	IBAD-MgO	Wang et al.^79^
YBa_2_Cu_3_O_7−δ_ (YBCO)	PLD	KrF excimer laser (λ = 248 nm); Laser energy: 250 mJ; Repetition rate: 60 Hz; Target–substrate distance: 4 cm; Deposition temperature: 800°C; Pure oxygen pressure: 200 mTorr^110^	NA	221 nm CeO_2_, 77 K, selffield = 4.6 MA/cm^2^	IBAD-MgO with CeO_2_ buffer layer	Wu et al.^113^
YBa_2_Cu_3_O_7−δ_ (YBCO)	PLD velocity filtration	A rotating disk with a window was used as a velocity filter. The window diameter: 8, 6, and 4 mm; Distance from the axis of rotation: 75 mm; Distance from the target: 30–35 mm; Rotational speed of the disc: 100 Hz. KrF excimer laser (λ = 248 nm); Pulse energy up to 200 mJ; Pulse duration 15 ns; Pulse repetition rate up to 20 Hz; Substrate temperature: 700°C–760°C Pressure: 0.4–0.8 Torr of the oxidizer gas (nitrous oxide N_2_O or oxygen O_2_); At the end of the deposition, the temperature of the substrate heater was lowered to 500°C, after which the deposition chamber was filled by air to a pressure of 1 atm.	81.5 K	40 MA	SrTiO_3_	Il’in et al.^129^
YBa_2_Cu_3_O_7−δ_ (YBCO)	PLD	KrF UV excimer laser (λ = 248 nm); Pressure: 0.1 mPa; Target-substrate distance: 45 mm; Power density: 2.0 J/cm^2^; Laser frequency: 2 Hz; The O_2_/Ar with the mass flow rate ratio of 100:0, 75:25, 25:75; The deposition pressure (O_2_/Ar): 15 Pa; The substrate temperature: 795°C; After the deposition, O_2_ was input into the chamber to 0.1 MPa before the films were cooled to 520°C at the rate of 10°C/min; For the full oxidation of the YBCO, the temperature was maintained at 520°C for 30 min; The samples were then cooled down to room temperature at the rate of 5°C/min.	∼90 K	NA	SrTiO_3_	Dai et al.^76^
YBa_2_Cu_3_O_7−δ_ (YBCO)	PLD	Excimer XeCl laser (λ = 308 nm); Pulse duration: 25 ns; Repetition rate: 60 Hz; Laser pulse energy:100–280 mJ; Oxygen pressure: 0.1–0.6 Torr; Substrate temperature: 750°C; After growth, the samples were annealed in pure oxygen at one atmosphere for 20 min at 650°C.	92.1 K	1.6 MA/cm^2^ (77 K)	LaAlO_3_ (001)	Xiong et al.^90^
YBa_2_Cu_3_O_7−δ_ (YBCO)	PLD	KrF excimer laser (λ = 248 nm); The laser energy: 350 mJ; Substrate temperature 800°C; The oxygen pressure: 200 mTorr; The laser repetition rate: 60 Hz; YBCO films thickness: 400 nm; YBCO films were quickly cooled to room temperature.	NA	4 MA/cm^2^ (77 K, self-field)	(RABiTS) Ni-5%W tapes, then CeO_2_ seed layer, YSZ barrier layer, and CeO_2_ cap layer	Xiao et al.^110^
YBa_2_Cu_3_O_7−δ_ (YBCO)	PLD	KrF excimer laser (LPX220); Laser beam incident angle: 45°; After depositing the silver layer as a protection layer, the YGBCO films were annealed in flowing O_2_ gas at 500°C for 4 h	NA	40 nm (CeO_2_+In) = 140 A (77 K, self-field)	CeO_2_/IBAD-MgO/Y_2_O_3_/Al_2_O_3_/Hastelloy C276 substrates	Liu et al.^112^
YBa_2_Cu_3_O_7−δ_ (YBCO) doped with BZO and Y_2_O_3_	PLD	KrF excimer laser; Energy: 450 mJ Repetition rate: 8 Hz; Oxygen pressure: 40 Pa; Followed by annealing at 500°C in an O_2_ atmosphere for 30 min.	Undoped YBCO (ave.) ∼89 K; 825°C deposition (ave.): 2% BZO +3%Y_2_O_3_ ∼83 K; 4% BZO +3% Y_2_O_3_ ∼85 K; 6% BZO +3% Y_2_O_3_ ∼87 K; 810°C deposition (ave.): 2% BZO +3% Y_2_O_3_ ∼85 K; 4% BZO +3% Y_2_O_3_ ∼88 K; 6% BZO +3% Y_2_O_3_ ∼87 K;	YBCO: 30 MA/cm^2^ (0 T, 5 K) 7 MA/cm^2^ (9 T, 5 K) 3 MA/cm^2^ (0 T, 65 K) 5 kA/cm^2^ (9 T, 65 K) 2% BZO YBCO: 50 MA/cm^2^ (0 T, 5 K) 8 MA/cm^2^ (9 T, 5 K) 8 MA/cm^2^ (0 T, 65 K) 20 kA/cm^2^ (9 T, 65 K) 2%BZO+3% Y_2_O_3_ YBCO: 40 MA/cm^2^ (0 T, 5 K) 10 MA/cm^2^ (9 T, 5 K) 5 MA/cm^2^ (0 T, 65 K) 0.2 MA/cm^2^ (9 T, 65 K)	SrTiO_3_ (STO) single crystal	Sebastian et al.^78^
YBa_2_Cu_3_O_7−δ_ (YBCO)	PLD	Nd-YAG laser (λ = 266 nm); The deposition process was off-axis, i.e., with the substrate surface perpendicular to the target; Deposition time: 5, 10, 30 min; Substrate temperature: 670°C–710°C; Oxygen pressure: 300 mTorr; Target-substrate distance: 2, 3, 4 cm; All the samples underwent the same annealing process, at 500°C, 1 atm of O_2_ during 30 min. Subsequently, they were slowly cooled to nearly room temperature in the same O_2_ atmosphere.	30 min deposition time: 690°C (Td), 4 cm (d) ∼83 K; 670°C (Td), 3 cm (d) ∼85 K; 670°C (Td), 2 cm (d) ∼87 K; 710°C (Td), 2 cm (d) ∼88 K; 690°C (Td), 2 cm (d) ∼89 K; 690°C (Td), 2 cm (d): 30 min (td) ∼88 K; 10 min (td) ∼81 K; 5 min (td) ∼79 K;	NA	SrTiO_3_ (STO) single crystal	Favre et al.^92^
YBa_2_Cu_3_O_7−δ_ (YBCO)	PLD	Excimer XeCl (λ = 308 nm) laser; Pulse duration: 25 ns; Repetition rate: 5 Hz; Laser fluence: 1.3 J/cm^2^; Deposition temperature (undoped films): 730°C; Target-substrate distance: 32 mm; Oxygen pressure: 23 Pa; After the deposition, an oxygenation step of 10 min at 700°C in 1 atm of pure O_2_ was made for all the films.^130^	Silver paste: STO ∼90 K; MgO, LSAT, SLAO, NGO ∼87 K; Mask (2): STO, SLAO, MgO ∼87 K LSAT ∼90 K;	Silver paste (0 T, 10 K): STO ∼80 MA/cm^2^; LSAT, SLAO ∼70 MA/cm^2^; MgO ∼20 MA/cm^2^; NGO ∼1.5 MA/cm^2^ Mask (2) (0 T, 10 K): STO ∼50 MA/cm^2^; MgO, SLAO ∼15 MA/cm^2^; LSAT ∼7 MA/cm^2^; NGO ∼0.5 MA/cm^2^	SrTiO_3_ (STO), MgO, (LaAlO_3_)_0.3_(Sr_2_AlTaO_6_)_0.7_ (LSAT), SrLaAlO_4_ (SLAO), and NdGaO_3_ (NGO), all in the (001) orientation.	Huhtinen et al.^115^
YBa_2_Cu_3_O_7−δ_ (YBCO)	PLD	A KrF excimer laser (λ = 248 nm, τ = 30 ns); To improve deposition reproducibility, the laser beam was cropped with an aperture so that only the part with the most uniform energy distribution passed to the target; Target-substrate distance: ∼60 mm; Energy densities on target: 1.5–1.8 J/cm^2^; Ambient gas pressure: 0.5–1 mbar; Scanning of the 2.5 × 2.5 mm^2^ laser spot across the target surface was in a meander pattern inside a square area; To avoid atmospheric contaminations, the target surface was cleaned and repolished; Substrate temperature: 770°C; Pressure: 0.8 mbar; Ar/O_2_ flow ratio 8/2 sccm; Repetition rate: 2 Hz; Deposition rate: ∼0.165 nm/s; p Post-deposition oxygenation in 500 mbar O_2_ at 450°C for 1 h.	Best performing ∼87 K	NA	(001) SrTiO_3_	Mozhaev et al.^87^
YBa_2_Cu_3_O_7−δ_ (YBCO)	Ultrahigh-Speed PLD	XeCl excimer laser (λ = 308 nm); Maximum pulse energy: 550–650 mJ; Pulse duration: 22 nm ± 5 nm; Maximal repetition rate: 200 Hz; Energy density: 3–10 J/cm^2^	NA	Ic: 500–700 A/cm-width, Jc: <1.5 MA/cm^2^ (77 K, self-field)	YSZ/Cr-Ni stainless steel	Rutt et al.^74^
YBa_2_Cu_3_O_7−δ_ (YBCO) with BaZrO_3_ (BZO)	PLD	KrF excimer laser (λ = 248 nm); Pulse length: 25 nm; Laser repetition rate: 2–4 Hz; Target-substrate distance 5.5 cm; Heater block temperature: 775°C–790°C; Dense targets: 83–90%; Oxygen partial pressure: 300 mTorr; Post-deposition anneal at 500°C and 1 atm of oxygen	Multiple: ∼89 K Single: ∼88–90 K	77 K, self-field: Multiple: 4 MA/cm^2^; Single: 5 MA/cm^2^ (0 vol % BZO); 2 MA/cm^2^ (2 vol % BZO)	LaAlO_3_, SrTiO_3_	Haugan et al.^88^
YBa_2_Cu_3_O_7-x_ (YBCO) with BaZrO_3_ (BZO) and Y_2_O_3_	PLD	KrF excimer laser (λ = 248 nm); Fluence:1.6 J/cm^2^; Laser energy: 450 mJ; Repetition rate: 8 Hz; Oxygen pressure: 300 mTorr; Heater block temperatures: 825°C for 7 min; After deposition, the films were annealed for a 30-min dwell time at 500°C and an oxygen atmosphere.	81.19 K (2% BZO), 87.69 K (4% BZO), 87.80 K (6% BZO)	NA	SrTiO_3_	Chen et al.^97^
YBa_2_Cu_3_O_7-x_ (YBCO) + BaZrO_3_ (BZO)/YBa_2_Cu_3_O_7-x_ + Y_2_O_3_ (multilayered)	PLD	KrF excimer laser; Alternating targets; Substrate temperature: 800°C; Oxygen partial pressure: 28 Pa; After completion of the YBCO thin-film deposition, the films were cooled down to 500°C in 5 10^4^ Pa oxygen and held at this temperature for 1 h before cooling down to room temperature.	NA	2.66 MA/cm^2^ (77 K, self-field) 0.3 MA/cm^2^ (77 K, 2 T)	LaAlO_3_ (100)	Liu and Du^98^
YBa_2_Cu_3_O_7-x_ (YBCO) + BaZrO_3_ (BZO) (5 mol %)	PLD	Deposition temperature: 745°C–840°C; Oxygen pressure: 200 mtorr; Laser repetition rates: 2–15 Hz	NA	Up to 3 MA/cm^2^ at 75.5 K, self-field; strong Jc retention at high fields	SrTiO_3_	Maiorov et al.^93^
YBa_2_Cu_3_O_6+x_ (YBCO)	PLD	Deposition temperature: 790°C; Oxygen pressure: 0.3 Torr; The same number of pulses and exactly the same laser fluency for all samples.	4.1 g/cm^3^: ∼88.7 K 4.3 g/cm^3^: ∼88.3 K 5.0 g/cm^3^: ∼89.3 K	10 K, 0 T: 4.1 g/cm^3^: 32 MA/cm^2^ 4.3 g/cm^3^: 22.2 MA/cm^2^ 5.0 g/cm^3^: 29.9 MA/cm^2^	SrTiO_3_ (100)	Paturi et al.^84^
YBa_2_Cu_3_O_7-x_ (YBCO) pure or + BaZrO_3_ (BZO) (1.5, 3.5, wt %); ErBa_2_Cu_3_O_7-d_ (ErBCO) + BaSnO_3_ (BSO) (2, 4 wt %)	PLD	ArF excimer laser (λ = 198 nm) Repetition rate: 1 Hz; Laser energy: 400 mJ; Oxygen pressure: 400 mTorr; Substrate temperature: 715°C (0, 1.5, 3.5wt %); 730°C (2, 4 wt %)	0 wt %: 90.3 K 1.5 wt %: 91.3 K 3.5 wt %: 86.9 K 2 wt %: 87.1 K 4 wt %: 86.4 K	0 wt %: ∼1 MA/cm^2^ (77. 3 K; 0T) ∼7 kA/cm^2^ (77. 3 K; 5T) 1.5 wt %: ∼3 MA/cm^2^ (77. 3 K; 0T) ∼0.2 MA/cm^2^ (77. 3 K; 5T) 3.5 wt %: ∼2 MA/cm^2^ (77. 3 K; 0T) ∼60 kA/cm^2^ (77. 3 K; 5T) 2 wt %: ∼3 MA/cm^2^ (77. 3 K; 0T) ∼60 kA/cm^2^ (77. 3 K; 5T) 4 wt %: ∼0.8 MA/cm^2^ (77. 3 K; 0T) ∼60 kA/cm^2^ (77. 3 K; 5T)	SrTiO_3_ (100)	Sueyoshi et al.^99^
YBa_2_Cu_3_O_7-δ_ (YBCO) + Tb (0.1, 1, 10%) or + Ce (0.1, 1, 10%)	PLD	KrF excimer laser (λ = 248 nm) Laser pulse rate: 4 Hz; Laser fluence: 3.2 J/cm^2^ Target-substrate distance: 6 cm; Oxygen pressure: 300 mTorr O2; Deposition temperature 780°C	0%: 89.2 K Tb 0.1%: 88 K Tb 1%: 89 K Tb 10%: 89.5 K Ce 0.1%: 88.9 K Ce 1%: 88.5 K Ce 10%: 84.8 K	77 K, 0.01 T: 0%: 2.23 MA/cm^2^; Tb 0.1%: 3.13 MA/cm^2^; Tb 1%: 2.68 MA/cm^2^; Tb 10%: 3.43 MA/cm^2^; Ce 0.1%: 1.65 MA/cm^2^; Ce 1%: 2.1 MA/cm^2^; Ce 10%: 0.52 MA/cm^2^; 77 K, 2 T: 0%: 0.14 MA/cm^2^; Tb 0.1%: 0.107 MA/cm^2^; Tb 1%: 0.162 MA/cm^2^; Tb 10%: 0.1 MA/cm^2^; Ce 0.1%: 0.099 MA/cm^2^; Ce 1%: 0.056 MA/cm^2^; Ce 10%: 0.001 MA/cm^2^;	SrTiO_3_ (100), LAO (100)	Kell et al.^94^
YBa_2_Cu_3_O_7-x_ (YBCO) pure or + BaZrO_3_ (BZO) + Y_2_O_3_	PLD	KrF excimer laser (λ = 248 nm) Laser frequency: 5 Hz; Target–substrate distance: 5 cm; Deposition temperature of 780°C–800°C; Oxygen pressure: 200 mTorr; After deposition, the films were cooled to room temperature with an oxygen pressure of 200–300 Torr. All films were then annealed in an oxygen atmosphere at 550°C for 30 min.	NA	65–75.5 K, self-field: 5.8 MA/cm^2^ at 0.2 μm thickness 2.3 MA/cm^2^ at 6.4 μm thickness	SrTiO_3_	Zhou et al.^105^
YBa_2_Cu_3_O_7-x_ (YBCO)	PLD	KrF excimer laser (λ = 248 nm, 4 Hz); Deposition temperature: 775°C–825°C; Oxygen pressure: 300 mTorr	NA	77 K, self-field: 775°C: 2.22 MA/cm^2^; 790°C: 2.19 MA/cm^2^; 800°C: 2.20 MA/cm^2^; 825°C: 2.63 MA/cm^2^; 65 K, 3T: 775°C: 0.94 MA/cm^2^; 790°C: 1.02 MA/cm^2^; 800°C: 1.12 MA/cm^2^; 825°C: 1.3 MA/cm^2^;	SrTiO_3_, LaAlO_3_	Wang et al.^80^
YBa_2_Cu_3_O_7−δ_ (YBCO)	PLD	ND:YAG laser (λ = 266 nm); Repetition frequency: 10 Hz; YBCO thicknesses: 0.4–4.0 μm; Substrate temperature: (Ts0 = 850°C, Ts∞ = 1045°C); Oxygen partial pressure: 400 mTorr; Laser energy density: 1.25 J/cm^2^; In order to reduce oxygen deficiency, post-annealing in an oxygen atmosphere of about 1 atm was performed.	NA	2.0 MA/cm^2^ (77 K, 0 T)	IBAD-MgO	Sato et al.^82^
YBa_2_Cu_3_O_7−δ_ (YBCO) with BaHfO_3_ (BHO) doping	Off-axis PLD	KrF excimer laser (λ = 248 nm); Off-axis: Energy density: 2.7 J/cm^2^; Laser repetition rate: 9 Hz; Substrate temperature: 850°C; Substrate rotation: yes; Oxygen pressure: 0.7 mbar; Deposition rate: 0.4 Å/pulse; After the deposition, oxygen loading took place *in situ* during cool-down at 15 K/min in 400 mbar O_2_.	89-93 K	∼1 MA/cm^2^ (77 K, 0 T); ∼0.1 MA/cm2 (77 K, 3 T);	SrTiO_3_ (100)	Backen et al.^89^
YBa_2_Cu_3_O_7−δ_ (YBCO) doped with BaSnO_3_ (BSO)	PLD combinatorial	ND:YAG laser (λ = 266 nm); Target-substance distance: 20–40 mm; Laser energy density: 2.0 J/cm^2^; Repetition frequency:1 Hz The laser irradiated area on the target in ellipsoid shape with a major diameter of 2.1 mm and a minor diameter of 1.7 mm; Heater temperature: 900°C–920°C; Oxygen pressure: 40–300 Pa; After the deposition, the chamber was filled with oxygen to atmospheric pressure, and the substrate temperature was rapidly dropped to 400°C and kept for 30 min.	SrTiO_3_ substrate ∼90 K	SrTiO_3_ substrate ∼2.5 MA/cm^2^ (77 K, self-field)	MgO (100), SrTiO_3_ (100)	Ichino et al.^116^
YBa_2_Cu_3_O_7−δ_ (YBCO)	HR-PLD (VAA, QEH)	NA – some details could be found in the earlier publications of the research group^119^^,^^131^	NA	2–3 MA/cm^2^ (1 μm-thick); >1.4 MA/cm^2^ (3 μm-thick)	Yttria-stabilized zirconia (YSZ) buffer layer on stainless steel	Usoskin and Freyhardt ^120^
YBa_2_Cu_3_O_7−δ_ (YBCO)	PLD-QEH	Substrate drum with speed >20 rad s−1; Oxygen post-loading at about 500°C	84-89 K	309 A at 31 T, 500 A at 18 T, 1200 A at 5 T (4.2 K)	Yttria-stabilized zirconia (YSZ) buffer layer on Cr-Ni Stainless Steel	Abraimov et al.^121^
YBa_2_Cu_3_O_7−δ_ (YBCO)	PLD-TLAG	UV KrF excimer laser; Wavelength: λ = 248 nm; Pressure:10^−6^–10^−5^ mbar, Laser fluence: 2.5 J/cm^2^; Repetition rate: 5 Hz; Substrate temperature: 200−825°С; TLAG: slow (∼15°C/min) or fast heating ramps (∼115°C/min) up to temperatures 825°C–850°C at a PO_2_ of 0.01 mbar. Then a sudden increase in PO_2_ to 0.3–1 mbar inside the PLD chamber, and 1–2.1 mbar, in a customized furnace.	450 nm YBCO on LaMnO_3_ (LMO)/SrTiO_3_ (STO) - 77 K	450 nm YBCO on LMO/STO - 1.7 MA/cm^2^ (77 K, self field)	(001)STO, (020) or (101)LMO-buffered/(001)STO, and buffered metallic substrates of SuNAM Co. Ltd LMO/MgO^epi^/MgO^IBAD^/Y_2_O_3_/Al_2_O_3_/Hastelloy	Queraltó et al.^122^
YBa_2_Cu_3_O_7−δ_ (YBCO)	PLD-LAP	A Lamba Physik KrF excimer laser; Wavelength: λ = 248 nm; Fluence ∼2 J/cm^2^; 0.15 J/pulse; Laser pulse repetition rate: 50 Hz; Growth rate of ∼250 nm/min; Oxygen pressure 200 mTorr; The deposition temperatures: 750°C–850°C; After growth the films were oxygenated at 500°C in 760 Torr pO_2_ for 1 h	Best performing LAP RE + BYNO APC YBCO: (Y,Yb,Sm)123 + liquid + BYNO – 86.6 K	Best performing LAP RE + BYNO APC YBCO: (Y,Yb,Sm)123 + liquid + BYNO – 2.0 MA/cm^2^ (50 K, 5T); 7.7 MA/cm^2^ (10 K, 10 T)	SrTiO_3_ (STO)	Feighan et al.^125^
YBa_2_Cu_3_O_7−δ_ (YBCO)	PLD-VLS	KrF excimer laser (λ = 248 nm). VLS 3 steps: 1) PLD deposition of YBCO solid layer (200 nm) at pO_2_: 53 Pa, Ts: 850°C, and fL of 100 Hz using a pure YBCO sintered bulk. 2) Synthesis of Ba_3_Cu_7_O_10_ (BCO) liquid layer (50 nm) on the solid layer using a 10wt % Ag_2_O doped BCO sintered bulk. The conditions were pO_2_: 200 Pa, Ts: 850°C, and fL: 10 Hz. 3) Supply BHO-doped YBCO via liquid layer at pO_2_: 200 Pa, Ts: 910°C, and fL: 100 Hz using a 3vol % BHO-doped YBCO sintered bulk.	NA	PLD-VLS (B = 0 T): 65 K–2 MA/cm^2^; 70 K–1.9 MA/cm^2^; 77 K–1 MA/cm^2^;	IBAD-MgO with buffer layer CeO_2_	Ito et al.^126^
YBa_2_Cu_3_O_7−δ_ (YBCO)	MAPLE-PLD	2 mL of YBCO/isopropanol dispersion (1 wt %) into the target holder, then frozen by the liquid nitrogen. A pulsed ND:YAG laser (λ = 532 nm); Frequency: 10 Hz; τfwhm ≅ 200 μs; The laser fluence: 120 mJ/cm^2^; Laser spot size: 0.56 cm^2^; Substrate-target distance: 4.5 cm (vertical configuration); Substrate and target rotational speed: 25 rpm and 10 rpm, respectively; Pressure 1 μTorr with different irradiation times at 0.5 h, 1.0 h, 1.5 h, 2.0 h.	Expected <80 K	NA	Graphene nanosheets	Yang and Zhang^132^

**Figure 10:**
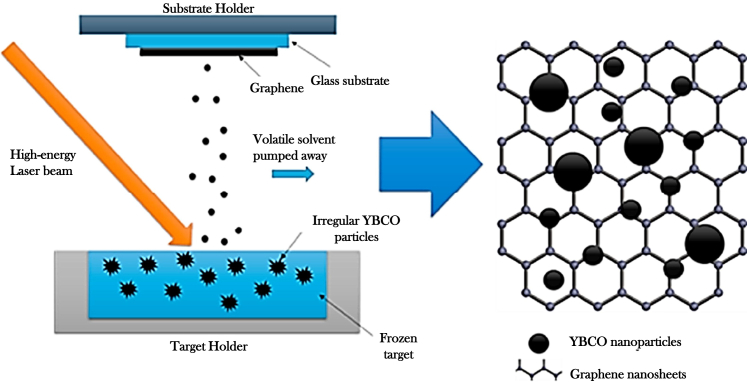
Schematic of the deposition of YBCO on graphene nanosheets by using MAPLE technique Reproduced from^132^ with permission from Elsevier.

One more innovative adaptation of PLD is matrix-assisted pulsed laser evaporation (MAPLE), designed to deposit delicate or composite materials such as YBCO nanoparticles onto graphene nanosheets.^132^ While both MAPLE and PLD are laser-based deposition methods, MAPLE introduces a solvent matrix that absorbs laser energy and gently releases the target material. In this study, YBCO particles suspended in isopropanol were frozen and used as the MAPLE target (Figure 10). The researchers demonstrated that YBCO microstructure could be tuned by adjusting the laser irradiation time: longer exposure led to increased YBCO loading on the graphene surface and a decrease in average nanoparticle size. Graphene served as an ideal substrate due to its excellent electrical and mechanical properties. MAPLE enabled a soft deposition process that preserved material integrity without the need for harsh chemical treatments. Although the superconducting properties of the resulting hybrid structures were not evaluated, the study showcased the potential of such YBCO-graphene nanosheets for various superconductor-based device applications.

A recent modification of the PLD technique introduced a velocity filtration system to selectively filter particles within the ablation plume based on their size and velocity during deposition.^129^ This approach employed rotating disks with apertures of 4, 6, and 8 mm in diameter to separate fast-moving, smaller particles from slower, larger ones. In the absence of filtration, the size of large particulates deposited in YBCO films ranged from 60 nm to over 1000 nm, contributing to significant surface roughness. Implementation of the velocity filtration mechanism effectively reduced the maximum particle size to below 500 nm. This reduction in both size and density of large particulates led to notable improvements in surface quality. The resulting smoother films were particularly advantageous for subsequent nanostructuring processes, such as electron beam lithography and argon ion etching, enabling the fabrication of well-defined micro- and nanostructures on HTS films. These findings emphasize the critical role of surface quality in precision nanofabrication for superconducting applications.

## Other REBCO materials

In addition to YBCO, a representative of the REBCO family, many other superconducting materials have been successfully synthesized using PLD^5^^,^^31^ This technique allows for the precise deposition of REBCO layers, offering exceptional control over the material’s microstructure, which is crucial for optimizing superconducting performance.^2^^,^^133^ Research has primarily focused on refining deposition conditions to enhance the superconducting properties of REBCO films, particularly through the introduction of APCs that improve current-carrying capacity in high magnetic fields.^133^

One of the pioneering studies on optimizing PLD parameters to enhance the superconductivity of Sm_1+x_Ba_2-x_Cu_3_O_6+σ_ (SmBCO) films was conducted by Sudoh et al.^134^ The crystal orientation and critical temperature (Tc) of SmBCO films were found to be highly sensitive to deposition conditions, such as substrate temperature, oxygen partial pressure, and target-substrate distance. Optimal deposition conditions (substrate temperature of 850°C and oxygen partial pressure of 0.4 Torr) led to a Tc of 91.5 K, highlighting the significant impact of precise control over PLD parameters. Under these conditions, SmBCO films exhibited improved crystal orientation and superconducting properties. Additionally, varying the composition with different x values (0.04, 0.08, and 0.12) not only influenced the crystal orientation but also enhanced the Tc, with higher x values facilitating better oxygen incorporation during film growth.

A study on TmBa_2_Cu_3_O_7-x_ (TmBCO) films^135^ demonstrated that the optimal deposition conditions for this material included substrate temperatures between 745°C and 805°C and oxygen partial pressures between 100 and 600 mTorr. Microstructural analysis revealed that TmBCO films deposited at 765°C exhibited a highly c-axis oriented structure, while higher oxygen pressure led to fewer pores, which potentially enhanced performance. Furthermore, TmBCO films deposited on SrTiO_3_ substrates exhibited a critical current density of 4.5 MA/cm^2^ at 77 K, while films on buffered metal substrates exhibited a Jc of 1 MA/cm^2^ at 77 K. The improved performance on STO substrates was attributed to the close lattice match between STO and TmBCO, which minimized lattice mismatch strain.

Wee et al. investigated the effects of substrate temperature on the performance of NdBa_2_Cu_3_O_7−δ_ (NdBCO) films, as well as the influence of film thickness.^136^ After PLD deposition at an optimal substrate temperature of 760°C, films underwent both *in situ* and *ex situ* annealing to further optimize their superconducting properties. The study revealed that NdBCO films with a thickness of 0.13 μm exhibited significantly higher Jc values compared to YBCO films (0.2 μm), particularly in high magnetic fields and when the field was applied parallel to the c-axis. This improvement was attributed to the higher density of pinning centers along the c-axis.

In another study, the growth temperatures of BaNb_2_O_6_ (BNO)-doped ErBa_2_Cu_3_O_7-σ_ (ErBCO) films were varied between 710°C and 760°C.^137^ The optimal growth temperature for achieving the highest Jc in magnetic fields was found to be 710°C, where the films exhibited the best superconducting properties. At this temperature, Ba (Er0.5Nb0.5)O3 (BENO) nanorods were thinner and more uniformly distributed, acting as effective c-axis correlated pinning centers, thus improving flux pinning. The Tc of BNO-doped ErBCO films remained high, ranging from 88 to 90 K, and their Jc in magnetic fields was significantly higher than that of pure ErBCO films. A subsequent study focused on the impact of growth temperature on ErBCO films doped with BENO instead of BNO.^138^ Two substrate temperatures, 710°C and 760°C, were tested, and as observed in the previous case, the lower temperature resulted in thinner, denser nanorods that were more effective as APCs, leading to improved superconductive characteristics.

Further investigation into the relationship between substrate temperature, nanorod size, and the effectiveness of APCs was conducted by Yuki et al.^139^ Their findings confirmed that lowering the substrate temperature led to a decrease in nanorod diameter, resulting in a higher number density of nanorods. This increase in pinning centers improved the superconducting properties of ErBCO films, confirming the significance of controlling substrate temperature during PLD for optimizing the superconducting performance of REBCO films.

These studies collectively demonstrate that controlling substrate temperature during PLD is a highly effective strategy for enhancing the superconducting properties of REBCO films.

Another study reported the enhancement of the critical current in BHO-doped EuBa_2_Cu_3_O_7_−α (EuBCO) films via a “multi-coating” PLD system.^140^ This setup employed a 60-mm-wide hot plate with five lanes to guide tape substrates beneath it, while four EuBCO + BHO plumes were generated by an excimer laser positioned at each corner of a square. This configuration enabled the deposition of multiple layers in a single pass, significantly improving productivity and layer homogeneity by ensuring uniform coverage over larger substrate areas. The method demonstrated potential for enhancing the homogeneity and density of pinning centers within the film, which could lead to improved critical current values. Furthermore, the a-axis oriented grains, known to impede superconducting current flow for YBCO,^82^ were found to be suppressed, resulting in superior in-field Ic performance. Even with increased thickness, Ic remained unaffected, unlike in conventionally fabricated tapes. Additionally, long EuBCO + BHO conductors, up to 200 m in length, were successfully fabricated, maintaining stable and uniform superconducting properties along their length.

In a further refinement of PLD, Ibi et al. utilized a four-plume system and optimized deposition conditions to fabricate EuBCO films with BHO nanorods at a high rate.^44^ Optimal parameters included a deposition temperature of 1145°C, a target-substrate distance of 97–100 mm, and an oxygen pressure of 600 mTorr with a 10 sccm oxygen flow. These conditions facilitated high Jc values and minimized the presence of a-axis oriented grains, while increasing the production rate to 10 m/h. The improved performance was attributed to the uniform distribution of BHO nanorods within the EuBCO matrix, enhancing flux pinning properties crucial for high-field applications. Building on these findings, the team later scaled up the EuBCO + BHO conductor fabrication process and successfully deposited a 93.7 m-long tape using a multi-plume PLD system.^141^ The tape exhibited an average Ic of 103 A/cm W (77 K, 3 T), with a low standard deviation of 4.1%, demonstrating the scalability and consistency of the fabrication process without compromising superconducting properties.

Subsequently, the group explored the multi-filamentary structure of EuBCO by controlling the spatial distribution of the plume and utilizing multiple laser beams to increase the deposition rate and achieve uniform conditions across a larger substrate area.^142^ This plane-plume PLD method allowed for the production of EuBCO-coated conductors with BHO doping and multi-filamentary structures, which proved effective in managing the coil’s shielding current and reducing losses due to alternating current. Notably, the filamentation approach led to a significant reduction in hysteresis loss (by a factor of 16 compared to non-filamentary conductors), resulting in enhanced performance with higher critical current density across operational conditions.

**Figure 11:**
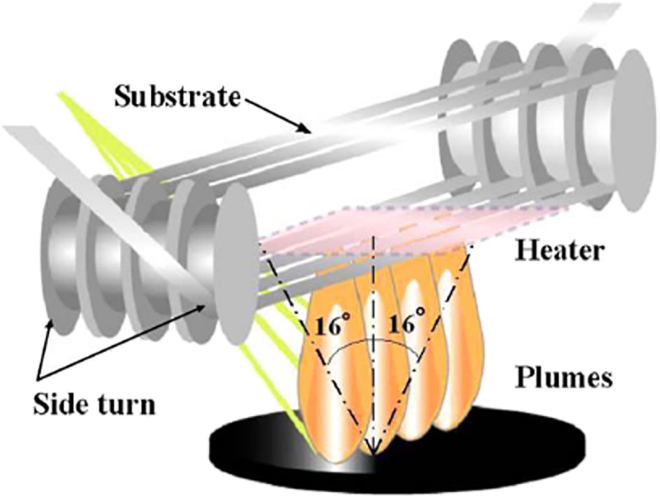
Schematic diagram of multi-plume and multi-turn (MPMT) PLD method Reproduced from^144^ with permission from Elsevier.

Other advancement in deposition techniques was the multi-plume and multi-turn PLD (MPMT-PLD) method, designed to increase both the deposition rate and area.^143^ This setup, illustrated in Figure 11, employs multiple laser plumes and multiple passes of the substrate over the plumes and target material, significantly expanding the deposition area while maintaining the quality of the film. The technique has been shown to improve critical current density and uniformity, making it ideal for the production of long-length coated conductors. For example, a 213-meter-long YBCO-coated conductor with an Ic of 245 A was successfully fabricated using this method, demonstrating its scalability for industrial applications.^144^ Similarly, MPMT-PLD was applied to synthesize BZO nano-rod-doped GdBa_2_Cu_3_O_7_−δ (GdBCO) films of 51.4 m, exhibiting an Ic of 204 A at 77 K in a self-field and improved current of 21–30 A at 77 K, 3 T.^145^ BZO nano-rods acted as effective c-axis correlated pinning centers, enhancing the performance of the tape in magnetic fields. The study also incorporated a multi-layer deposition strategy to maintain surface temperature consistency as the layer thickness increased, adjusting the temperature at each deposition round (850°C–900°C). Challenges associated with uniform growth over long tapes, such as inconsistent growth rates and deposition temperatures, led to further investigations into a turn-by-turn method, where test tapes were analyzed after each turn to monitor the evolution of the film’s microstructure and performance.^146^ This analysis identified defects such as voids and grain boundary disruptions, especially at certain deposition stages, and recommended the use of thermal shields and heating profile adjustments to improve deposition conditions.

In 2009, Lee et al.^147^ introduced the in-plume pulsed laser deposition (IP-PLD) technique, which focused on reducing the substrate-to-target distance to enhance deposition rates and improve film properties. This method provided superior control over the film composition and the formation of effective flux pinning centers, critical for maintaining high Jc values. Through the optimization of several parameters, including substrate temperature (780°C–850°C), oxygen partial pressure (300–500 mTorr), and laser energy density (2–4 J/cm^2^), the study revealed that a higher laser energy density facilitated the creation of linear defects that functioned as APCs. Additionally, the impact of stoichiometry and the APC effect was examined, with off-stoichiometric, Ba-poor targets combined with barium zirconate (BZO) proving particularly effective in suppressing Jc degradation and enhancing flux pinning by increasing c-axis grain density. Among the best results, GdBCO + BZO films (5 μm thick) achieved an Ic of 135 A/cm in a 3 T magnetic field at 77 K. Later, the same researchers explored the effects of increasing laser energy density (>0.03 J/mm^2^) and further reducing the target-substrate distance.^148^ This modification resulted in a 3-fold increase in the production rate while maintaining Ic values of 135 A/cm-width at 3 T and 77 K, and self-field Ic values ranging from 700 to 1000 A/cm-width for short 5 μm-thick films. The study highlighted the importance of precisely selecting and adjusting target composition to achieve the desired film characteristics.

Building on their earlier work, the same team also explored the in-plume multi-turn multi-plume PLD (MPMT-PLD) method, which reduced the target-substrate distance and utilized an off-stoichiometric REBCO target to effectively control the REBCO layer composition.^149^ The researchers also adjusted laser energy density and oxygen gas pressure to improve crystallinity and material quality during deposition. Optimal results were obtained with laser energy densities >3 J/cm^2^ and oxygen pressures >800 mTorr. While the Jc values achieved were lower than those obtained through conventional PLD methods, they were sufficient for practical applications. Notably, the GdBCO-coated conductors demonstrated impressive uniformity in Ic distribution, with a longitudinal standard deviation of only 0.6%. The optimized conditions in this in-plume PLD method enabled a production rate approximately double that of conventional techniques, thereby reducing the overall cost of REBCO layer production.

**Figure 12:**
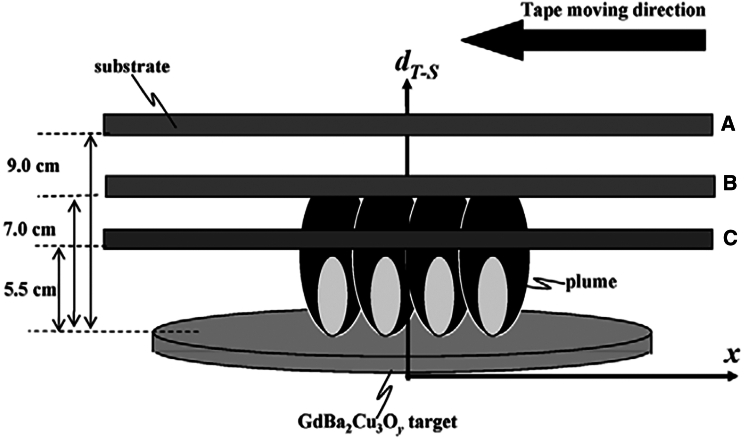
Schematic drawing of a reel-to-reel PLD system Reproduced from^150^ with permission from IOP.

In a subsequent study, Miura et al. investigated the effects of target composition, tape speed, and substrate distance on the stoichiometry of the GdBCO layer^150^ using the RTR-PLD system (Figure 12). By controlling these parameters, the researchers effectively managed the formation of undesirable a-axis-oriented grains, resulting in enhanced quality of the GdBCO-coated conductors. At higher tape speeds (20 m/h) and a smaller substrate distance (5.5 cm), the modified GdBCO conductors exhibited a critical current density of 2.6 MA/cm^2^ with a thickness of 1.2 μm over a meter length. This demonstrated significant improvement in both Jc and production speed, with increases of 1.8 and 3.0 times, respectively, compared to films fabricated under standard conditions.

The reel-to-reel multi-target PLD technique has also been utilized to investigate the influence of seed layers on the properties of YGBCO films.^151^ The role and effectiveness of seed layers were previously discussed in the context of conventional PLD methods.^81^^,^^82^ In this study, seed layers ranging from 2 to 30 nm in thickness were introduced to improve surface morphology and reduce internal residual stresses. The results demonstrated that the inclusion of a seed layer enhanced both the crystallinity and the critical current density (Jc) of the films. Optimal performance was achieved with a 15 nm thick seed layer, leading to a Jc of 4.17 MA/cm^2^ at 77 K in a self-field. Surface analysis indicated that while the seed layer contributed to a smoother surface, excessive thickness (30 nm) resulted in increased roughness. Another modification to the reel-to-reel PLD process, though indirect, involved the introduction of O_2_^+^ irradiation after the PLD deposition by Suzuki et al.^152^ This irradiation process added defects that served as additional pinning centers, thereby enhancing the flux pinning effect and improving Jc along the c-axis and across various magnetic field orientations. The optimal ion fluence for irradiation was determined to be 1×10^13^ ions/cm^2^. The irradiated samples exhibited a Jc increase of at least 20% in magnetic fields ranging from 0 to 9 T, compared to non-irradiated samples with the same composition.

In 2016, Ibi et al. aimed to enhance not only the deposition rate of EuBCO with BHO but also its critical current density, while maintaining high superconducting performance^153^ This improvement was achieved by transitioning from a vapor-solid (VS) growth mode to a vapor-liquid-solid (VLS) growth mode. The optimization involved adjusting deposition temperature (1145°C), oxygen pressure, and target composition (EuBa_2_Cu_3_._2_OX + BHO), which improved the crystallinity and connectivity of the EuBCO grains. This transition allowed for a significantly higher deposition rate (approximately 40 μm/h compared to 10 μm/h in VS mode) and an increased production rate (about 10 m/h), while still preserving high Jc values. Moreover, long EuBCO-coated conductors exhibited homogeneous longitudinal Ic distributions, reflecting consistent superconducting properties along the conductor’s length. These authors later further optimized the EuBCO superconductor by varying deposition and annealing temperatures in combination with the VLS growth technique and off-stoichiometric BHO-doped EuBa_2_Cu_3_._2_Ox.^154^ They found that high-temperature deposition coupled with low-temperature oxygen annealing was crucial in achieving high critical temperature (Tc = 93.9 K) and improved in-field performance. The optimal conditions for this process involved a deposition temperature of 1150°C and an oxygen annealing temperature of 280°C. These enhanced conditions were attributed to better alignment and distribution of BHO nano-rods, which significantly impacted material performance. Yokoe et al. later confirmed that the VLS growth mode resulted in superior superconducting properties, both in self-field and in external magnetic fields, compared to the VS mode.^155^ This improvement was linked to a lower volume fraction of misoriented grains within the c-axis-oriented EuBCO matrix in the VLS mode, which facilitated better grain alignment and fewer defects, thereby improving current flow.

**Figure 13:**
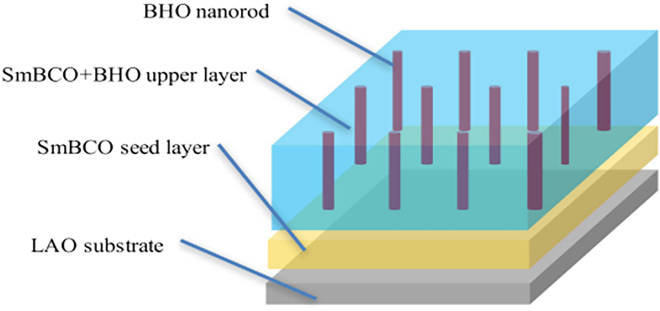
Schematic drawing model of the low-temperature growth (LTG) method Reproduced from^157^ with permission from IOP.

In efforts to further enhance deposition methods and achieve high-speed processes, the low-temperature growth (LTG)-PLD technique has been explored in relation to REBCO films.^156^^,^^157^^,^^158^ LTG involves initially growing a thin seed layer at high temperatures with excellent crystal orientation, followed by deposition of the main superconducting layer at lower temperatures (Figure 13). This approach helps reduce misfit between the superconductor and the substrate, enabling the growth of high-quality c-axis-oriented conductors at lower temperatures. Additionally, the LTG process increases the number and density of random APCs, improving flux pinning capabilities. Early studies utilizing the LTG-PLD technique focused on the structural and superconducting properties of pure SmBCO films.^158^ These films exhibited excellent Jc (0.28 MA/cm^2^ at 77 K, 5 T), comparable to NbTi at 4.2 K. Furthermore, the study revealed that an optimal Sm/Ba ratio (x = 0.04), along with the formation of Sm-rich phases acting as effective pinning centers, contributed to enhanced superconducting properties. Subsequent work on SmBCO films doped with BZO and BHO by LTG showed improved superconducting characteristics compared to non-LTG and undoped materials.^156^^,^^157^ In particular, the ability to optimize LTG growth temperatures and additive quality allowed for the modification of APC shapes to meet the desired superconducting properties. The LTG method also facilitated the fabrication of purely c-axis-oriented SmBCO films at lower substrate temperatures (750°C) compared to conventional high-temperature PLD methods, without significantly compromising Tc.^159^ This approach led to more densely packed and thinner BHO nanorods, further improving the pinning properties while maintaining a high Tc.

Matsumoto et al. further advanced the alternating-target PLD technique previously explored by Yoshida et al.,^157^ applying it to the GdBCO-BHO material system.^160^ In this approach, pure GdBCO and BHO targets were alternately ablated, enabling the *in situ* formation of BHO nanorods within the GdBCO matrix. The study revealed that the incorporation of BHO nanorods significantly improved flux pinning across a wide range of temperatures and magnetic fields. Notably, the samples with a laser pulse ratio of GdBCO/BHO of 20:1 (5% BHO) and 25:1 exhibited superior Jc compared to undoped GdBCO films. Structural analysis indicated that the combined effect of bent and inclined BHO nanorods, along with randomly distributed pinning centers, contributed to enhanced vortex pinning. These findings also suggested the potential advantage of introducing nanoparticles in tandem with nanorods to further optimize pinning—a strategy previously discussed in detail.^93^

**Figure 14:**
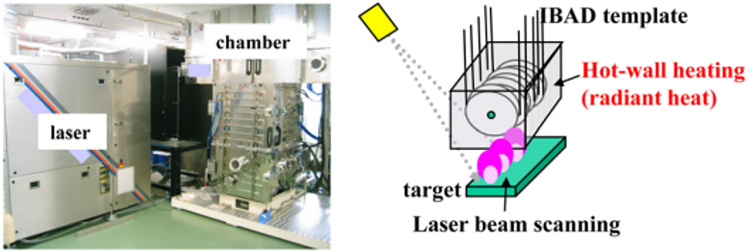
PLD system with laser beam scanning and hot-wall heating Reproduced from^162^ with permission from IOP.

In parallel, Japanese researchers employed the hot-wall PLD technique to achieve high-speed, stable, and homogeneous growth of REBCO films.^161^^,^^162^ This method simulates a furnace-like environment by ensuring uniform thermal radiation and maintaining a constant surface temperature during deposition, both critical for achieving high crystalline quality and doping uniformity (Figure 14). Using this approach, 20 cm-long and 6 μm-thick GdBCO films were fabricated with a high critical current of 997 A at 77 K in self-field, attributed to their high crystallinity and absence of impurity phases.^162^ Furthermore, accelerated deposition using a 360 W laser enabled the production of 1 μm-thick, 20 cm-long GdBCO films with an Ic of 352 A at a deposition rate of 60 m/h, without compromising superconducting performance. These results confirmed that the hot-wall heating system maintained high Jc values even at increased deposition speeds. Subsequently, this technique enabled the production of GdBCO tapes exceeding 100 m in length, each with consistently high current capacity (>200 A) and minimal Ic variation along their length.

More recently, hot-wall PLD was employed to fabricate long-length BHO-doped EuBCO tapes, with successful production of 45 m^163^ and later 300 m^161^ tapes demonstrating uniform critical current performance. The flux pinning properties of these BHO-doped EuBCO films were systematically investigated under different deposition rates.^164^ Remarkably, fast-grown films displayed comparable, and in some cases superior, superconducting performance relative to those deposited at slower rates. This demonstrated that hot-wall PLD is a viable method for scaling up production while preserving Jc uniformity and quality.

In addition to performance metrics, the same research group investigated the mechanical reliability of BMO-doped GdBCO and EuBCO films grown by hot-wall PLD under bending and axial tensile strain.^165^ While both doped and undoped films exhibited similar crystal orientations, BMO doping was found to increase strain sensitivity, resulting in more rapid Ic degradation under mechanical stress. Although the differences between GdBCO and EuBCO were minimal, the preliminary attribution of this behavior pointed to grain boundary distortions affecting current flow. These findings underscored the necessity of considering mechanical robustness alongside superconducting performance enhancements.

Further studies focused on the electromechanical properties of BHO-doped EuBCO films fabricated via hot-wall PLD.^166^ This work reaffirmed the method’s scalability and throughput potential for long-length tape production. The detailed investigation into the effects of bending and uniaxial strain revealed the fatigue behavior and critical current degradation under mechanical deformation. It was found that tapes fabricated on thinner substrates (e.g., 50 μm Hastelloy) exhibited minimal Ic loss, even under small bending radii. These results further highlighted the advantages of BHO doping combined with optimized substrate design for enhancing both the superconducting and mechanical resilience of REBCO-coated conductors.

Lao et al. conducted an in-depth investigation into the effects of novel dopants on the superconducting performance of PLD-grown GdBCO films, specifically focusing on the incorporation of 6 mol % BSO (BaSnO_3_) nanoparticles.^167^ Their findings revealed a significant enhancement in the critical current density, particularly near the matching field of 1 T, where the density of vortices corresponds to the density of APCs. This improvement was attributed to the efficient flux pinning provided by the well-dispersed BSO nanoparticles. Furthermore, it was observed that a slower deposition rate during PLD led to the formation of elongated, thinner BSO nanorods, which proved more effective in enhancing the critical current than their shorter, thicker counterparts formed at higher deposition rates. In a comparative study, Ovcharov et al. evaluated the performance of BSO and BZO (BaZrO_3_) nanoparticles as APCs embedded within the GdBCO matrix via PLD.^168^ While the inclusion of these nanoparticles slightly reduced the superconducting transition temperature to 91.9 K for BSO and 91.0 K for BZO, from a reference Tc of 93 K—the overall superconducting performance favored BSO due to its relatively higher Tc and Jc values.

In another study, ErBa_2_Cu_3_Oᵧ (ErBCO or Er123) films doped with varying concentrations of BZO nanorods (0, 1.5 wt %, and 3.5 wt %) were fabricated by PLD to assess their influence on microstructure, resistivity, and critical current density.^169^ The BZO nanorods effectively enhanced Jc under low magnetic fields through c-axis correlated pinning. However, at elevated fields, their contribution diminished, with edge dislocations emerging as the dominant pinning centers, indicating the need for further exploration of high-field pinning mechanisms. Maeda et al. investigated BHO (BaHfO_3_) nanoparticles as APCs within PLD-grown GdBCO films.^170^ Their detailed nanostructural analysis highlighted the uniform dispersion, morphology, and induced nanostrain of BHO nanoparticles, which collectively contributed to an increase in both Tc and the irreversibility field (Hirr), thereby enhancing the superconducting performance.

Zheng et al. explored the effects of partial substitution of Ho in HoBa_2_Cu_3_O_7_−δ (HoBCO) films with other rare-earth elements (Dy, Er, and Yb), resulting in the formation of Ho_0_._75_RE_0_._25_Ba_2_Cu_3_O_7_−δ (HoREBCO, where RE = Dy, Er, Yb) thin films synthesized via PLD.^171^ The introduction of these secondary rare-earth dopants induced lattice distortions that served as effective pinning centers, leading to a marked enhancement in Jc. Among the compositions studied, HoDyBCO exhibited the best performance, followed by HoErBCO and HoYbBCO. Interestingly, the optimal deposition temperatures for achieving maximum Ic varied with the atomic mass of the dopant: 780°C for Dy, 790°C for Er, and 800°C for Yb, suggesting that heavier rare-earth dopants require higher thermal energy to optimize film crystallinity and superconducting properties.

In a novel approach, Liu et al. designed a tri-layer architecture consisting of YGBCO/Gd-CeO_2_/YGBCO to mitigate the detrimental thickness effect typically observed in thicker superconducting films and to enhance interlayer electrical connectivity.^172^ The study systematically varied the Gd-CeO_2_ interlayer thickness, oxygen partial pressure, and laser energy during PLD. The optimal configuration—featuring a 10 nm thick interlayer deposited at an oxygen partial pressure of 20 mTorr and laser energy of 200 mJ—yielded superior superconducting performance. This was attributed to the balance between optimal oxygen availability for crystal growth and the preservation of interlayer conductivity. Notably, lower laser energies resulted in a sharp decline in superconducting properties, emphasizing the necessity of sufficient energy to maintain film integration and electrical continuity. The optimized tri-layer films demonstrated improved Ic values compared to single- and double-layer YGBCO films, as the interlayer effectively facilitated current tunneling, thereby enhancing overall superconducting performance.

## Large-scale REBCO production by PLD

2G-HTS Second-generation high-temperature superconducting tapes provide a range of distinct advantages, including high critical current densities, excellent performance across diverse magnetic field environments, and lower raw material costs. Currently, several industrial-scale manufacturers—such as Shanghai Superconductor Technology Corporation, Ltd. (SSTC), Faraday Factory Japan (FFJ, formerly SuperOx), SuperPower, American Superconductor Corporation (AMSC), Fujikura, SuNAM, Bruker, Theva, and others—are capable of routinely producing and supplying 2G-HTS tapes on a commercial scale.^173^^,^^174^ In parallel, numerous large-scale HTS cable projects have been initiated for power grid applications in countries including China, South Korea, the United States, Japan, and Germany.^174^ These initiatives involve the testing and validation of various cable architectures and power ratings across different sites worldwide to assess their operational reliability and performance. In the following section, we provide a detailed overview of research studies focused on tapes produced by these leading industrial entities.

### Shanghai Superconductor Technology Corporation, Ltd.

In 2013, SSTC launched a pilot production line for coated conductor fabrication, achieving a major milestone by increasing deposition speed to 100 m/h. This advancement played a pivotal role in enhancing the production of REBCO tapes, supporting their commercialization.^175^

A notable development was the shift from RABiTS (rolling-assisted biaxially textured substrates) to IBAD-MgO (ion beam assisted deposition of MgO) templates, a transition that markedly improved the in-plane alignment and overall film quality, both essential for high-performance superconducting tapes. The PLD process was systematically optimized to ensure superior surface morphology and texture, resulting in critical current values exceeding 500 A/cm over 100-meter-long conductors, with ongoing efforts targeting kilometer-length tapes.

SSTC also investigated a simplified architecture involving PLD-REBCO/Sputter-CeO_2_/IBAD-MgO multilayers on metallic substrates.^176^ This configuration was intended to streamline fabrication and enhance scalability. Utilizing a reel-to-reel PLD system operating at 100 m/h, REBCO films demonstrated impressive critical current densities, surpassing 3.0 MA/cm^2^ for 1 μm films and maintaining over 2.5 MA/cm^2^ for 2 μm films at 77 K in self-field. The underlying high-quality texture significantly contributed to these results.

In an effort to address the performance limitations of thick REBCO films—important for mechanical robustness and higher power throughput—researchers explored films exceeding 1 μm in thickness.^177^ It was observed that Jc decreased by approximately 1.5× due to the growth of a-axis-oriented grains. To mitigate this, YBCO films were doped with Y_2_O_3_ and partially substituted with Gd, successfully suppressing a-axis grain formation and enhancing superconducting performance. A 2.8 μm-thick doped YGBCO film achieved an Ic of 780 A/cm at 77 K in self-field, and continuous 100 m-long tapes with Ic > 500 A/cm were successfully fabricated.

Another effort focused on BHO-doped EuBCO for magnet applications.^178^ Multiple deposition passes were employed based on critical current requirements. Improvements to the PLD heating system enhanced substrate contact and minimized thermal gradients, which significantly improved film homogeneity. Resultant films reached thicknesses of 2.7 μm with an average Jc of 1.9 MA/cm^2^ (77 K, self-field). Tapes demonstrated reliable Ic uniformity, exceeding 400 A/cm-width over 1 km lengths. The facility’s production capacity is expected to exceed 200 km annually for 10 mm-wide coated conductors.

In collaboration with Shanghai Jiao Tong University, SSTC deposited and tested YGBCO with 5 mol % BHO, achieving Jc values of 3.2 MA/cm^2^ in short samples and enabling high-throughput PLD (>100 m/h) for long-length production^179^ Kilometer-scale tapes delivering >300 A/cm were demonstrated. Current efforts involve incorporating high-density nanoscale APCs such as BZO, Y_2_O_3_, Sr_2_Nb_2_O_7_, and CeO_2_ to further enhance performance and expand operational applicability.

To further improve the PLD process, a novel thermal management approach—radiation-assisted conductive heating (RACH)—was implemented for GdBCO deposition.^180^ By combining radiative and conductive heating and precisely controlling thermal shielding elements, substrate temperature uniformity was significantly improved (ΔT ±4°C vs. ±15°C in conventional systems). This reduced thermal relaxation time and resulted in a >50% improvement in in-field Ic at 30 K and 4.2 K. Tapes with >2 μm thicknesses displayed Jc values up to 3 MA/cm^2^ at 77 K in self-field with exceptional uniformity across lengths exceeding 300 m.

Further optimization of the RACH method enabled the fabrication of 300 m-long GdBCO tapes with average Ic > 520 A/cm-width and standard deviation below 2%.^181^ High-rate-grown EuBCO+BHO and YGBCO tapes (at 40 nm/s) demonstrated excellent performance, exceeding 280 A/4 mm-width at 30 K, 5 T, and nearly doubling at 4.2 K, 10 T. The enhanced properties were linked to liquid-assisted growth and higher dislocation densities from stacking faults, acting as efficient APCs. Additionally, SSTC has advanced jointing and lamination techniques critical for industrial implementation. Copper-stabilized tapes, in particular, showed high yield strength and maintained superconducting properties under tight bending.

Another notable contribution from Shanghai International Superconducting Technology Co., Ltd. was the development and deployment of a 35-kV HTS cable system for urban grid applications.^174^ The cable, 1.2 km in length, replaced four traditional XLPE cables with a three-core design incorporating a cryostat, significantly reducing spatial requirements. It employed 2G HTS tapes as conductor and shielding layers, rated for 2.2 kA at 77 K. Although detailed PLD parameters for this application were not disclosed, the project marked a significant milestone in real-world superconducting cable deployment.

### Faraday factory Japan ( before SuperOx)

**Figure 15:**
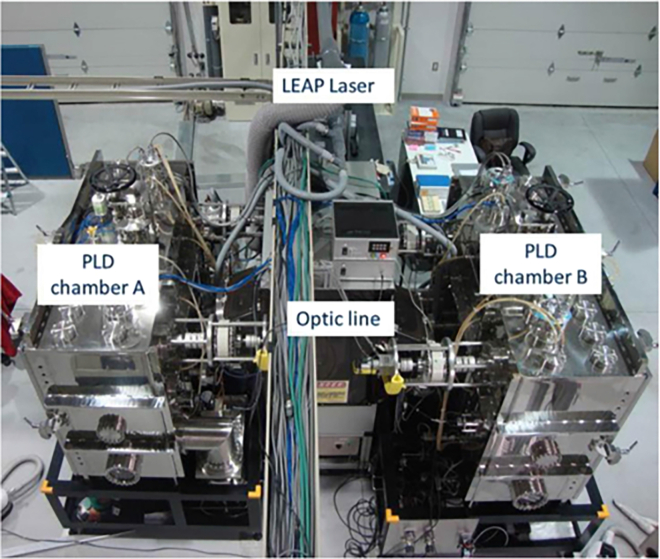
General view of the “one laser–two chambers” PLD system at SuperOx Japan, LLC Reproduced with permission from.^72^

Faraday Factory Japan, formerly SuperOx, has developed a cost-effective industrial-scale PLD system with a dual-chamber setup using a single laser source. The system, featuring two chambers from AOV Co., Ltd. and a LEAP industrial excimer laser, alternates the laser beam between chambers to optimize laser use and increase throughput (Figure 15).^72^

A key feature of the design is the shared use of critical components—power supply, optical path, and control units—which significantly reduces the overall capital investment required for the PLD system. The PLD process was carefully optimized to achieve high deposition rates: up to 100 m/h for buffer layers and 15–30 m/h for GdBCO superconducting layers. Fine-tuning of parameters such as substrate temperature, buffer layer thickness, and tape translation speed (typically 20–30 m/h) enabled the successful production of 100-meter-long, 12 mm-wide 2G HTS tapes with critical currents exceeding 300 A.

The longest tapes produced to date have reached lengths of 200–250 m, with minimum critical current values ranging from 200 to 250 A per 12 mm width. FFJ’s overarching objective remains the industrial-scale production of several hundred kilometers of HTS tape per year, with the goal of making PLD-based coated conductor manufacturing economically viable on a global scale.

APC-doped GdBCO films were deposited using the industrial PLD system at SuperOx under high-throughput production conditions, with the goal of enhancing superconducting performance without compromising manufacturing efficiency.^182^ BaSnO_3_ (BSO) and BaZrO_3_ (BZO) were employed as APCs in varying concentrations: BSO at 6%, 12%, and 18%, and BZO at 6%. Growth rates were controlled by tuning the laser pulse frequency, yielding deposition rates of 375, 560, and 750 nm/min.

Among the samples, GdBCO films doped with 6% BSO and fabricated at lower growth rates demonstrated higher critical current (Ic) values across a broad temperature and magnetic field range compared to undoped GdBCO. Similarly, GdBCO doped with 6% BZO exhibited superior Ic values over the undoped counterpart, though the improvement was only significant at 20 K and 4.2 K. In general, films grown at lower deposition rates exhibited enhanced critical current performance, likely due to improved crystalline quality and more favorable diffusion dynamics during film formation.

In a 2021-year study, researchers also demonstrated industrial-scale manufacture of second-generation YBCO coated conductors in which vortex pinning was provided exclusively by uniformly distributed intrinsic Y_2_O_3_ nanoparticles, thereby avoiding the process complexity associated with engineered columnar defects.^183^ Using pulsed-laser deposition, ∼2.4 μm-thick YBCO layers were grown on 40 μm Hastelloy C276 substrates, and more than 300 km of 4 mm-wide tape was produced within nine months. Representative samples attained engineering current densities above 1 kA mm^−2^ at 20 K and 20 T, surpassing the 700 A mm^−2^ requirement for the SPARC fusion magnet program and reaching >2 kA mm^−2^ at 4.2 K, 20 T. Across the production lot, the ratio of 20 K, 20 T to 77 K, self-field critical current (“lift-factor”) exhibited a standard deviation of ∼15%, confirming reproducibility. These results established that a simplified microstructure can meet or exceed high-field performance targets and provide a scalable route toward cost-effective, high-volume REBCO conductors for fusion and other demanding applications.

The performance of SuperOx conductors was also benchmarked against commercial tapes from SuNAM and SuperPower, which utilize different deposition techniques^184^ Among the compared samples, the GdBCO conductor from SuperOx showed the second-highest performance, with a critical current density of approximately 3 MA/cm^2^. The highest Jc, around 4.2 MA/cm^2^, was achieved by SuNAM’s conductor, fabricated using the reactive co-evaporation method.

### Other

One of the compact pilot line (CPL) PLD systems was also evaluated by Rutt et al.,^74^ enabling the fabrication of long-length REBCO coated conductors ranging from 40 to 100 m. Several key modifications were introduced to the CPL system, including the implementation of the quasi-ellipsoidal heating (QEH) principle and a redesigned target motion mechanism. The QEH approach was applied similarly to earlier studies by Usoskin et al.^116^^,^^119^ and Abraimov et al.^121^

The target motion was refined to incorporate two types of movement: a linear back-and-forth scan at constant speed, and periodic 180° rotations after each full laser scan. This dual-directional motion ensured uniform ablation across the target surface, allowing for consistent film deposition over extended durations. The system employed a high total laser repetition rate of up to 200 Hz, distributing the pulses over a large target area (320 cm^2^), which resulted in a low local pulse rate of approximately 1 Hz. Experiments were conducted to assess the influence of laser pulse energy on the thickness and properties of the YBCO films. A wide pulse energy range (3–10 J/cm^2^) was explored, and an optimal deposition rate of 3.8 Å/pulse was identified—approximately three times higher than typical rates. Under these conditions, the critical current reached 500–700 A/cm-width, despite a moderate reduction in critical current density (Jc < 1.5 MA/cm^2^).

In a related effort, Fujita et al. investigated the pre-industrial production of GdBCO conductors using PLD, fabricating tapes with lengths up to 500 m.^185^ The conductors were evaluated under various magnetic fields and low-temperature conditions, providing valuable data for scaling up and designing practical applications. The synthesis process was optimized to consistently produce 500 m-long tapes with Ic values of 500 A/cm-width at 77 K in self-field. Notably, higher Ic values were obtained for thicker films; for instance, a 5.5 μm-thick film exhibited Ic values of 637 A/cm-width at 5 T and 976 A/cm-width at 40 K.

## Limitations and future directions

### Cost and scalability challenges

Scaling up PLD from lab-scale samples to industrial-length REBCO tapes has been a central challenge.^43^^,^^186^ Traditional PLD was a batch process on small substrates, but coated conductor production demands continuous reel-to-reel deposition on flexible metal tapes. In recent years, significant progress has been made: reel-to-reel PLD systems now routinely fabricate tens to hundreds of meters of tape in a single run.^176^ Innovations, such as high-power, high-repetition lasers, enhanced heating systems and others have pushed tape throughput to over 100 m/h travel speeds while maintaining film quality.^176^^,^^180^ Despite its technical merits, the PLD approach for REBCO tapes faces economic challenges. The current fabrication cost of 2G HTS conductors is relatively high (≥$100 per kAm),^2^ owing to expensive equipment, low throughput in some process steps, and the need for high-quality materials. PLD in particular requires high-cost components (e.g., high-power excimer or solid-state lasers, high-vacuum chambers, and precision motion systems) and has room for improvement in increasing the deposition speed per unit area.

Reducing the cost per meter of REBCO tape is crucial for competitiveness, especially against conventional low-temperature superconductors and other HTS fabrication routes. Generally, a decrease in the cost could be achieved by upgrading the performance of films, increasing the production rates (throughput and yield), and improving process efficiency. One cost driver is the complex multilayer architecture (substrate, buffers, superconducting layer, and stabilizers)—each layer adds processing time and expense. Efforts are underway to optimize the tape architecture: for example, using fewer buffer layers or replacing vacuum-based steps with faster chemical processes where possible.^2^^,^^31^

Another cost factor is the throughput of the deposition tools. As noted, new high-speed PLD systems, for example, with higher laser repetition, hot-wall chambers capable of maintaining uniform thermal profiles, MPMT setups, and others, help lower costs by coating more tape in less time.^74^ However, these must also be balanced against operating costs like laser and target wear, higher power consumption. Additionally, in practice, some researchers have tested the hybrid processes, for example, liquid-assisted like TLAG/LAP-PLD to leverage both the higher supersaturation, which results in better film performance, and higher growth speed.^125^^,^^126^

Future directions to cut costs also include attempts to use other more cost-advantageous parts, for instance, Nd:YAG laser.^116^^,^^117^^,^^118^ Since the Nd:YAG crystal is pumped optically rather than by rare-gas-halide mixtures, it removes supply bottlenecks and toxic-gas handling costs. Recent improvements in cavity design, beam-shaping, and power scaling now deliver flat-top profiles and highly stable fluence. As a result, epitaxial REBCOs were possible to be deposited with smooth surfaces and lower droplet densities. Such advances signal the feasibility of compact, low-maintenance PLD lines whose capital and operating costs could be an order of magnitude lower than today’s excimer-based installations. Further cost reductions could also hinge on maximizing target utilization. Re-threading the tape through several consecutive plumes in the MPMT reel-to-reel layout already captures a majority of ablation flux, resulting in high target yield and targeting further improvement.^144^^,^^145^^,^^149^^,^^187^^,^^188^ Other approaches also focus on improving yield by reducing the fraction of tape that gets rejected or requires rework, as the approximate highest yield in industry is currently ∼80%.^2^ These related challenges will be discussed in more detail in the following part 6.2.

Scaling up manufacturing volume will also naturally drive down unit costs via economies of scale. Generally, various possible technology improvements are being examined that facilitate cost-saving opportunities, and these were extensively discussed in Parts 3–5 of this review. The long-term goal is to achieve coated conductor costs low enough for widespread commercial adoption, without compromising the exceptional performance enabled by the PLD process. To sum up, although PLD equipment and operating expenses remain above other solution processes like chemical methods, the reliability and reproducibility of PLD continue to justify its use in applications where performance margin is critical, and in the meantime, the efforts are directed to further cost reduction methods.

### Performance reproducibility challenges in long-length tapes

As the demand for technological applications of 2G-HTS in various industrial fields is constantly growing, the importance of high-quality long tape production is increasing as well. Achieving consistent performance (critical current, uniform field behavior, etc.) across kilometers of REBCO tape is also one of the challenges, though.^43^^,^^186^ Even when average properties are excellent, variability along the length and between batches can hamper reproducibility. It is well-documented that coated conductors show segment-to-segment critical current variation along a long tape, as well as batch-to-batch differences for tapes made under nominally the same conditions. These variations arise from slight process fluctuations or microscopic defects that occur during fabrication—for example, a small region with lower thickness or a secondary phase inclusion can reduce the local critical current.^189^^,^^190^^,^^191^^,^^192^ Inhomogeneous film thickness can lead to critical current density variations and local weak spots in the superconductor. Additionally, multi-element oxide films like REBCO can exhibit slight compositional non-uniformity across the plume footprint (e.g., variations in Ba/Cu ratio at the edges) and non-uniform defect density, which further contribute to performance variability.^85^^,^^189^^,^^190^^,^^191^^,^^192^^,^^193^^,^^194^ In present manufacturing, non-destructive scanning tools (e.g., continuous 77 K scanning of critical current along the tape) are used to map tape performance and identify weak sections.^189^^,^^195^^,^^196^ If a section falls below spec, producers may cut it out or splice tapes, but this is labor-intensive and undesirable for applications needing long, continuous lengths.

Thus, a major future direction is improving the uniformity and reproducibility so that every meter of tape meets the performance requirements without intervention. This entails tighter control of deposition conditions across time—for instance, maintaining substrate temperature, laser energy, and plume composition uniformly throughout a run—and rapid feedback control to correct any drifts^120^^,^^180^^,^^197^. Future improvements are also focusing on advanced plume engineering—using multiple beam spots or tailored ambient gas conditions to broaden the deposition area—and real-time thickness monitoring to ensure homogeneity.^144^^,^^145^^,^^146^^,^^147^^,^^148^^,^^149^^,^^150^ Improved buffer and substrate engineering also play a role, since better buffer uniformity can translate to more uniform REBCO growth (more details in sections 3.6 and 3.7).^2^^,^^31^ Additionally, researchers are investigating the fundamental limits of variability: recent studies suggest that even a small transverse variation in Jc (across the tape width) can influence magnet performance, underscoring the need for homogeneity in all dimensions.^198^ Addressing film uniformity is critical, as even minor gradients or clustered defects can limit the attainable current in large coils and induces uneven stress during operation.

Enhancing tape reliability is another aspect of reproducibility. This includes ensuring that the tape’s performance is maintained after handling, winding into magnets, or thermal cycling. Mechanical defects or delamination that varies along the tape length can cause localized weak spots and overall performance loss.^199^^,^^200^^,^^201^^,^^202^^,^^203^ Going forward, more advanced in-line monitoring techniques (inspection of texture, thickness, composition) will be developed and implemented during manufacturing to prevent or catch any deviations early. Together with process improvements that eliminate sources of defects, these measures aim to deliver long REBCO tapes with highly reproducible, high critical current performance end-to-end. Achieving this level of uniformity will increase yield and reduce the need for splices, thereby improving both the technical reliability and economic viability of REBCO-coated conductors. Thus, many of these various improvement attempts were covered in detail in the previous parts 3–5.

Artificial intelligence (AI) and ML will play an important role in the advancement of manufacturing, quality assurance, and generally in REBCO development. The next part will discuss this in more detail, providing the practical cases where, for example, machine vision and learning algorithms help to improve production consistency and detect anomalies that traditional inspection techniques might miss.

## Artificial intelligence and machine learning for superconductors development

**Figure 16:**
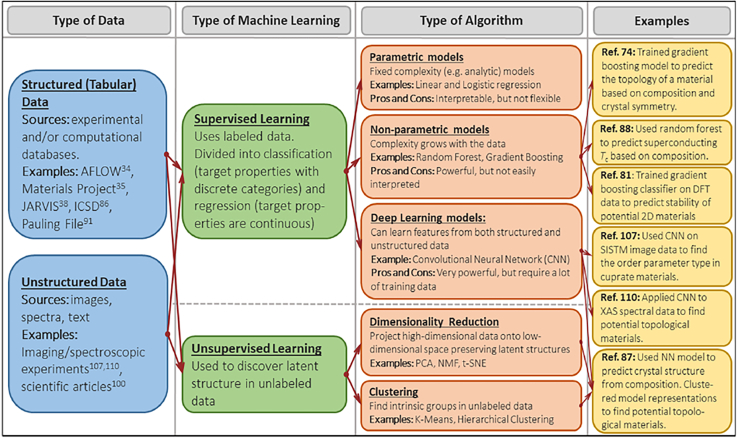
Commonly used machine-learning tasks and algorithms Reproduced from^205^ with permission from Nature.

AI and ML are increasingly being applied across scientific disciplines, and the field of superconductors is no exception. Recent studies demonstrate that AI and ML are actively used to accelerate the development and improve the properties of superconducting materials.^204^^,^^205^^,^^206^^,^^207^^,^^208^ By analyzing large datasets and uncovering complex patterns, these algorithms can facilitate the discovery of new superconductors, optimize existing compositions, and predict material behavior under various environmental and operational conditions (Figure 16). The integration of these advanced computational tools not only expedites experimental workflows but also paves the way for theoretical insights, thereby enhancing the efficiency, scalability, and overall performance of superconductors. Ultimately, AI and ML are poised to significantly contribute to the upscaling of superconductor fabrication and their broader deployment across diverse applications.

Considering specifically the integration of AI/ML into the development of REBCO superconductors via PLD represents a transformative advancement in the field of high-temperature superconductivity. PLD is inherently complex, with superconducting film quality being highly sensitive to a multitude of interdependent parameters such as laser fluence, substrate temperature, oxygen partial pressure, target composition, and others, as was described earlier. Traditional trial-and-error approaches are insufficient for navigating this multidimensional parameter space efficiently, particularly when optimizing critical performance metrics such as critical current density and critical temperature. ML algorithms could potentially offer powerful tools to model these non-linear relationships, enabling predictive control over film growth and accelerating the identification of optimal deposition conditions. Furthermore, AI-driven *in situ* monitoring and closed-loop control systems enhance process stability and reproducibility, which would also be essential for the large-scale manufacturing of REBCO-coated conductors. These data-driven approaches not only reduce the cost and time of development cycles but also facilitate materials discovery by identifying novel doping strategies and microstructural configurations that enhance flux pinning and superconducting performance. As such, AI and ML are poised to play a central role in the next generation of scalable, high-performance REBCO superconductor technologies; therefore, this section was introduced to discuss the current and potential use of AI/ML in the superconductors field.

Although the amount of research on the use of AI/ML specifically in PLD-deposited REBCO field is yet limited, several studies have already demonstrated some promising results. For instance, Cai et al. showed a practical integration of ML into PLD workflows by applying support vector regression (SVR), optimized with particle swarm optimization (PSO), to predict the superconducting transition temperature and relative resistance ratio of YBa_2_Cu_3_O_7_ films based on deposition parameters.^209^ Compared to traditional regression, SVR provided more accurate predictions and identified substrate temperature and target density as the most influential factors. The same research group also applied this PSO-optimized SVR to predict the superconducting transition temperature of NdBa_2_Cu_3_O_7-_δ (NBCO) thin films fabricated by PLD.^210^ Using experimental data from 17 samples varying in temperature, pressure, and laser energy, the SVR model demonstrated superior accuracy compared to traditional multivariable nonlinear regression (MNR), achieving a mean absolute error of 0.22 K and an R^2^ (the goodness of fit parameter) of 0.94 (MNR—0.68 K and 0.71, respectively). The model also predicted an optimal deposition condition yielding a maximum Tc of 92.0 K, showcasing its potential for guiding experimental optimization in superconductor film fabrication.

More recently, another research team also demonstrated the integration of Gaussian process regression (GPR), a ML model, into the materials development workflow for HTS.^211^ The authors modeled the superconducting transition temperature of YBCO based on lattice parameters affected by various dopants. The GPR model achieved high prediction accuracy (correlation coefficient of 99.78%) and low error rates, significantly outperforming prior support vector machine (SVM) models. By enabling efficient and low-cost Tc estimation, this AI-based approach could inform experimental decisions, such as dopant selection and synthesis parameters, thus highlighting its utility in streamlining superconductor optimization processes and paving the way for autonomous materials design.

Zhang and Xu, in turn, developed a multiple-linear-regression framework that linked seven PLD variables—substrate temperature, oxygen pressure, laser energy density, pulse count, repetition rate, target-surface state, and target density—to the superconducting transition temperature Tc and the relative resistivity ratio of YBCO films.^212^ Applied to a 17-sample literature dataset, the model reproduced experimental Tc and resistivity ratio with >99.99% correlation and near-zero RMSE, surpassing earlier support-vector-regression and multiple-regression approaches while remaining computationally inexpensive. Leave-one-out cross validation yielded average errors of roughly 2 K for Tc, corroborating its robustness. Consequently, the study provided an efficient predictive tool and quantitative insight for tailoring PLD growth of REBCO coatings toward higher Tc and controlled normal-state transport, thereby facilitating rapid process optimization without exhaustive experimentation.

A practical implementation of AI-driven process control in PLD workflows was demonstrated in the roll-to-roll production of second-generation HTS tapes, where a real-time machine vision system was integrated to monitor surface grayness levels as a proxy for deposition temperature.^213^ By correlating the grayness scale with actual substrate temperature and the critical current of resulting tapes, the system enabled early detection of process anomalies and improved coating quality. This approach allowed dynamic, non-contact temperature tracking and provided actionable data for adjusting deposition parameters, offering a concrete step toward autonomous process control in superconducting film manufacturing.

Although there is still a limitation of AI/ML use cases specifically in PLD-deposited REBCO area, generally ML has already been employed in various facets of superconductivity research, with several key directions emerging in recent years. In this review, we will also highlight four major avenues of ML application in this field at the moment. The first area involves the discovery and design of novel superconducting materials. For instance, clustering algorithms have been utilized to identify structural and chemical similarities between known superconductors and unexplored compounds, thereby generating promising candidate materials for experimental validation. The second direction focuses on the prediction of superconducting properties—most notably, the critical temperature—based on a range of known physical and chemical parameters. These predictive models can significantly narrow the search space for high-performance superconductors. The third avenue involves the application of ML and AI to accelerate and optimize experimental procedures, including the synthesis (AI/ML-PLD integrations), testing, and characterization of superconducting materials. These approaches have demonstrated considerable success in reducing the time and resources required for experimental workflows. Moreover, the fourth avenue that will be briefly covered is the use of AI/ML in HTS cables optimization, which does not necessarily improve the PLD deposition workflow, but facilitates the more effective large-scale applications of REBCO tapes.

In this section, we will examine representative studies that exemplify these three research directions. A comprehensive survey of all ML and AI methodologies applied to superconductivity is beyond the scope of this review, and the reader is referred to several detailed reviews on the subject.^204^^,^^205^^,^^206^^,^^207^^,^^208^^,^^214^^,^^215^ Instead, our goal here is to emphasize the pivotal role of ML and AI in advancing superconductor research by both enhancing discovery processes and expediting experimental progress.

### Prediction of the superconducting properties

Numerous studies have been conducted to predict superconducting properties, such as the critical temperature (Tc), using a variety of material parameters, including many recent ones like research done by Gashmard et al., where authors used the CatBoost algorithm, enhanced by two custom-developed packages—Jabir and Soraya—for feature generation and selection, respectively, to predict the Tc.^216^ The model, trained on a meticulously curated dataset of 13,022 compounds, achieved high prediction accuracy (R^2^ = 0.952, RMSE = 6.45 K), outperforming existing models and demonstrating the critical influence of thermal conductivity among 322 features on Tc prediction.

One other notable investigation was carried out by Stanev et al.,^217^ who developed a ML framework comprising multiple models to identify potential superconducting candidates from a dataset of approximately 110,000 compositions sourced from the norganic crystal structure database (ICSD). Using compositional features derived from Magpie descriptors, their classification model achieved an accuracy of 92% in distinguishing materials with Tc values exceeding 10 K. Additionally, a random forest regression model yielded a coefficient of determination (R^2^) of 0.88, indicating strong predictive performance. The framework identified 35 promising compounds—some structurally and compositionally akin to cuprates—for experimental validation, showcasing the ability of ML to reveal latent patterns in complex materials datasets.

In a different approach, Konno et al.^218^ developed a deep learning model based solely on chemical composition, encoded directly from the periodic table, to predict Tc for superconductors in the SuperCon database. The neural network (NN) achieved an R^2^ of 0.92, further demonstrating the potential of deep learning for property prediction from elemental features alone. For cuprates specifically, it was noted that they were more prone to over- and underestimation issues due to their performance’s high sensitivity to compositional changes. Impressively, the model successfully predicted recently discovered superconductors such as CaBi_2_ and Hf_0_._5_Nb_0_._2_V_2_Zr_0_._3_, which were not present in the training database, reinforcing the robustness of the approach.

Similarly, another study employing random forest regression attained an R^2^ of 0.92 for SupCon data using a feature set that included elemental ratios, electron affinity, atomic number, melting temperature, and other physical parameters.^219^ Roter et al.^220^ also explored both supervised and unsupervised ML techniques on SuperCon data for Tc prediction and classification of materials as superconducting or non-superconducting. Their k-nearest neighbors (KNN) classification model achieved an impressive accuracy of 96.5%, while the Bagged Tree regression model delivered an R^2^ of 0.93 and a root-mean-square error (RMSE) of approximately 8.91 K. Interestingly, their findings suggested that traditional physical descriptors such as valence electron count, electronegativity, and covalent radius had limited influence on the models’ performance, implying that compositional information alone may be sufficient for accurate prediction.

Other studies have taken a more structurally driven approach. For instance, simple SVR models using only lattice parameters have been shown to effectively predict Tc in Fe-based superconductors and doped MgB_2_ systems.^221^^,^^222^ Although narrower in application, these models underscored the viability of using basic crystallographic features in superconductivity research.

An additional noteworthy contribution comes from Xie et al.,^223^ who employed symbolic regression techniques to derive an enhanced analytical expression for Tc prediction. Their method improved the traditional Allen-Dynes formula, which is widely used for estimating Tc in electron-phonon mediated superconductors, offering a more accurate, data-driven alternative grounded in physical theory.

### Potential superconducting materials

Several recent investigations have aimed to accelerate the discovery and characterization of superconducting materials by integrating advanced ML techniques with large-scale materials databases. These efforts have demonstrated the potential of data-driven methods to both predict and validate new superconducting candidates.

One notable contribution is the development of CRYSPNet, a ML framework tailored to predict the crystal structures of inorganic compounds, with particular relevance to superconducting materials.^224^ CRYSPNet utilizes NNs to forecast key crystallographic features—such as Bravais lattice type, space group, and lattice parameters—solely from chemical composition. Trained on a dataset comprising over 100,000 entries from the ICSD, the model exhibited predictive accuracy surpassing that of conventional computational approaches. This capability is particularly valuable in the search for novel superconductors, as it enables the identification of compounds likely to exhibit superconducting behavior based on their predicted structural features. Moreover, the model’s internal representations (NN weights) for Bravais lattice predictions were used as input for density-based spatial clustering of applications with noise (DBSCAN). This clustering analysis effectively grouped known cuprates and related compounds, allowing for the identification of structurally analogous, yet previously unexplored, candidates for experimental validation.

**Figure 17:**
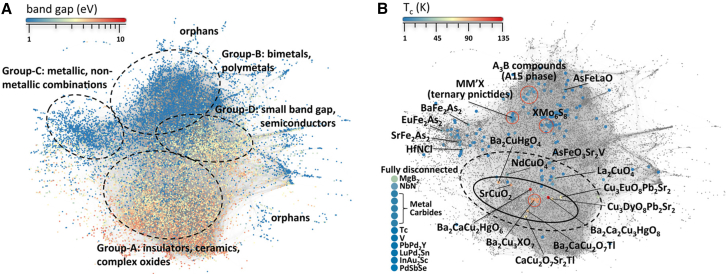
Mapping of material properties (A) band gaps and (B) superconducting critical temperatures.

In another innovative approach, Isayev et al.^225^ introduced a fingerprinting methodology based on features derived from electronic band structures. This technique enabled the generation of “cartograms”—visual mappings of materials’ electronic and structural properties—which facilitated rapid identification of superconductors (Figure 17). By projecting complex data such as bandgap and critical temperature onto interpretable maps, their method enabled the efficient screening of large materials spaces for potential high-Tc superconductors. The approach highlights the versatility of ML in capturing non-trivial relationships between electronic structure and macroscopic material properties, offering a powerful tool for materials discovery beyond traditional descriptors.

Another innovative study employed convolutional neural networks (CNNs) to learn and predict material properties based on the structured organization of elements within the periodic table.^226^ By using the periodic table as a spatially meaningful input, the CNNs were able to capture complex chemical relationships and effectively predict key material characteristics, such as lattice parameters and enthalpy of formation, for full-Heusler compounds. To enhance the model’s generalization capability, the researchers implemented a transfer learning strategy: the CNNs were initially trained on a large dataset from the open quantum materials database (OQMD) and subsequently fine-tuned on a smaller, high-quality dataset from the ICSD. This two-step training process enabled the model to identify potentially stable and unexplored compounds—including those containing tungsten—that are seldom reported yet hold promise as superconducting materials.

In a complementary direction, Meredig et al.^227^ addressed the challenges of assessing ML models in the context of materials discovery, particularly the limitations of conventional cross-validation (CV) techniques. Traditional CV methods often produce overly optimistic performance estimates because they fail to account for the extrapolative nature of materials discovery tasks, where predictions are frequently required for materials that are chemically and structurally dissimilar to those in the training set. To overcome this, the authors introduced the leave-one-cluster-out cross-validation (LOCO-CV) method, which partitions the data into chemically distinct clusters and ensures that entire clusters are excluded during training. This approach provides a more rigorous and realistic evaluation of a model’s extrapolation capabilities. LOCO-CV is especially valuable for identifying novel superconductor candidates, as it allows researchers to assess whether an ML model can successfully generalize to previously unseen classes of materials—an essential requirement for true materials innovation.

### Acceleration and advancement of synthesis, experimental testing, and characterization of superconductors

Another significant avenue for the application of ML and AI in superconductivity research lies in the synthesis processes and analysis of complex experimental data to uncover hidden patterns and emergent phenomena that are often elusive to traditional analysis methods. These techniques have demonstrated the capability to improve the depositions and reveal subtle electronic orders and physical quantities critical to understanding high-temperature superconductivity. Generally, many studies have been recently done on integration between AI/ML tools and specifically PLD, which can be potentially applied for superconductor materials deposition as well.

**Figure 18:**
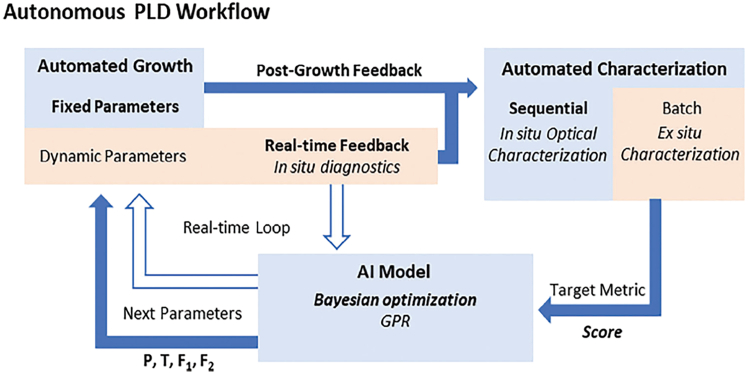
Workflows for autonomous PLD synthesis Reproduced from with permission from^229^ John Wiley and Sons.

Harris et al. (2024) demonstrated the use of (2 + 1)D CNNs to extract deep spatiotemporal features from intensified-CCD (ICCD) image sequences of plasma plumes generated PLD.^228^ These features enabled real-time monitoring of the plume and anomaly detection due to the high correlation between the ICCD images and synthesis parameters such as chamber pressure and laser energy. Moreover, this approach allowed to accurately predict the growth kinetics of WSe_2_ thin films (mean r^2^ = 0.847) derived from *in situ* laser reflectivity data. The combined model using both image and process parameters outperformed individual models, showcasing the potential of integrating *in situ* optical diagnostics with ML for feedback control and pre-screening in autonomous thin film synthesis workflows. Building on the insights of the previous related work, the group next developed the entire automated PLD workflow that integrated real-time optical reflectometry, *in situ* Raman spectroscopy, multi-target co-ablation, and a ten-position substrate exchanger with a cloud-linked Gaussian-process Bayesian-optimization (BO) engine.^229^ This “auto-PLD” system (Figure 18) sampled only ≈0.25% of a four-dimensional parameter space (substrate temperature, background pressure, and independent laser fluences on WSe_2_ and Se targets) yet autonomously located two narrow growth windows for high-quality, monolayer-thickness WSe_2_ films, delivering millimeter-scale uniformity, sub-nanometer roughness (∼0.46 nm RMS) and grain-resolved crystallinity while achieving at least a 10-fold increase in experimental throughput compared with conventional, manually operated PLD workflows. The study, thus, demonstrated that combining high-throughput automation with active-learning algorithms can dramatically accelerate process-property mapping in PLD, providing a transferable framework for optimizing complex oxide and REBCO superconducting films where similarly vast parameter spaces hinder empirical exploration.

Later, the same research group also introduced a physics-informed, online Bayesian state-estimation framework that fused a layered growth kinetics model with *in situ* laser-reflectivity data to quantify nucleation and lateral growth rates during pulsed-laser deposition in real time.^230^ After verifying the algorithm with synthetic data, they applied it to WSe_2_ and, after that, embedded it in an autonomous PLD platform to monitor the early-stage synthesis of 1T′-MoTe_2_ across a broad pressure window. The filter reliably converged on kinetic parameters after only ∼15% monolayer coverage—well before secondary-layer nucleation—yielding rate constants consistent with full post-growth fits and remaining robust for a variety of growth rates. This capability transformed reflectivity into a quantitative feedback signal, paving the way for model-predictive or reinforcement-learning control loops that can steer complex oxide, chalcogenide, or cuprate (e.g., REBCO) films toward metastable or phase-pure states with minimal trial-and-error.

In terms of detecting hidden patterns in the superconductors’ data, multiple research initiatives were also performed. For instance, artificial neural networks (ANNs) have been employed to analyze image arrays of electronic quantum matter (EQM).^231^ In this study, ANNs were applied to copper oxide Mott insulators, leading to the discovery of previously undetected symmetry-breaking states that were both lattice-commensurate and periodic over four-unit cells. These states, hidden within the raw data, were revealed by the network’s ability to detect subtle, non-obvious spatial patterns. The identification of such electronic orders contributes substantially to our understanding of the microscopic mechanisms underlying high-temperature superconductivity, particularly in the cuprate family.

In a related effort, another research group leveraged Boltzmann machines to extract hidden physical quantities from angle-resolved photoemission spectroscopy (ARPES) data.^232^ This unsupervised ML model enabled the separate extraction of normal and anomalous self-energies, two quantities central to describing the electronic interactions in superconducting materials. These self-energies are typically obscured in raw ARPES data due to overlapping and canceling features. However, the ML analysis uncovered prominent peak structures in both types of self-energy, revealing critical information about the pairing mechanisms that lead to high critical temperatures. This work underscores the potential of ML techniques to extract deep physical insights from noisy or convoluted experimental datasets—insights that may be inaccessible via conventional analytical approaches.

Moreover, Talantsev and Mataira^233^ introduced novel visualization and analysis techniques for large datasets in applied superconductivity, with a particular focus on critical current measurements in HTS. They proposed the use of polar coordinate projections, where the radial and angular axes represented distinct physical parameters. This approach offered an intuitive and multidimensional representation of complex data, enabling researchers to identify trends and anomalies more effectively. Such visualization techniques support the optimization and engineering of HTS materials for practical applications by improving the interpretability of large experimental datasets.

Collectively, these studies demonstrate the transformative potential of ML and AI methods in advancing superconductivity research—not only by accelerating discovery but also by offering profound insights into the underlying physics of novel superconducting systems.

### HTS cables optimization

One more capable direction of the AI/ML tools integration is the optimization of high-temperature superconducting (HTS) cables—especially those employing REBCO conductors. Generally, the cables’ improvement process poses a complex challenge due to the non-linear behavior of superconducting materials, the intricacies of cryogenic environments, and the interdependence of thermal, electrical, and mechanical properties. AI/ML offer powerful frameworks for navigating this multidimensional design space. By enabling predictive modeling, sensitivity analysis, data-driven fault detection, and other advancements, AI/ML approaches are reshaping how HTS cables are designed, evaluated, and operated, making them more efficient, reliable, and cost-effective for large-scale applications.

The role of AI and ML in HTS cable optimization begins with their ability to serve as surrogates for computationally expensive simulations. Sadeghi and Song developed an intelligent surrogate model based on the fault current, fault energy, fault duration, fault type, and fault resistance data to predict the short-circuit fault performance of REBCO-based HTS cables.^234^ The model used cascade forward neural networks (CFNNs) and showed high accuracy (average 99.1%) results. That allowed engineers to conduct robust sensitivity analyses without relying on time-intensive finite element simulations. This not only improves model interpretability but also accelerates the design-validation cycle in fault-prone scenarios.

Expanding on this foundation, Bonab et al. introduced a ML framework tailored for reconstructing HTS-coated conductors’ index-values, which describe the steepness of the transition from superconducting to normal resistive state, with high *n* values being more desired for applications needing fast response and high stability.^235^ The authors applied several ML models for *n* value prediction depending on various temperature and magnetic conditions, observing also the CFNN with the highest accuracy. This paves the way for facilitating rapid modeling of new REBCO HTS conductors and advancing their application in superconducting devices.

Fatigue-induced degradation of superconducting tapes under cyclic electrical loading is a critical reliability issue. Recently, it was addressed by applying AI models—particularly decision trees—to predict critical current degradation due to repetitive overcurrent cycling.^236^ Interestingly, among various models, radial basis function neural network (RBFNN) showed the most accurate, consistent, and robust results due to handling of non-linearity, sample-specific behavior, and local variance with minimal error, all while generalizing well and being computationally efficient. Thus, this work contributed to proactive reliability management and adaptive control in HTS systems.

AI’s application extends into practical manufacturing and quality assurance as well. For example, Wang et al. examined how winding quality affects the performance of spiral REBCO-tape cables and introduced an AI-assisted inspection framework to enable real-time quality control.^237^ The authors showed that non-uniform winding produced indentations that lowered the tapes’ local critical current by > 10 A and reduced its longitudinal uniformity, thereby limiting overall cable capacity. To prevent such defects in automated production lines, the authors built a residual-network-based Richer convolutional features model that segments optical images of the cable and classifies each winding interval as correct or erroneous. A 120-image, manually labeled training set (augmented by flips and rotations) plus transfer learning from BSDS500 underpinned the model, while thresholding, hole filling, and noise removal data postprocessing converted probability maps to binary masks suitable for feedback control. Benchmarked on six unseen images shot under different lighting, the developed detector outperformed conventional edge-detection methods: on correctly wound tapes, its recall—the fraction of all ground-truth interval pixels that the network labels correctly—stayed above 90%, while shadows sometimes lowered performance on wide, misaligned gaps. This work illustrated how machine vision and learning algorithms can improve production consistency and detect anomalies that traditional inspection techniques might miss.

Finally, Zhu et al. combined a three-dimensional H-formulation finite-element model with an optimized multilayer-perceptron NN to accelerate the prediction of AC losses in soldered or mechanically bonded REBCO lap joints, a critical heat-load contributor in high-temperature-superconducting cables.^238^ Simulation data spanning 30–150 Hz, 0.3–0.9 Ic, and 0.01–0.2 μΩ joint resistance were validated experimentally and then used to train the ANN, cutting computation time from ∼35 min per case to <1 s while retaining mean-squared-error ≈2 × 10^−2^. This fast neural-network model let the authors run many simulations and learn two key things: if joint resistance varied more widely (following a Weibull spread), total losses in the cable climbed by about 50%; and by charting the highest resistance that still meeted a fixed loss limit at each frequency, it was found that joints can tolerate higher resistances as frequency rises. The work demonstrated that data-driven models can replace heavy finite-element runs in joint-level design and in system-level cooling assessments for REBCO PLD tapes.

More broadly, Yazdani-Asrami and colleagues reviewed a spectrum of AI applications across the HTS life cycle, including design optimization, condition monitoring, and operational control.^208^ Their survey emphasized the use of hybrid AI architectures that integrate deep learning with physics-informed constraints, offering a roadmap for embedding intelligence across all stages of HTS cable development.

In summary, the studies in this field illustrate a paradigm shift in HTS cable engineering—from rule-based and simulation-heavy approaches to adaptive, intelligent systems capable of learning from data and improving over time. This convergence of superconductivity and AI is not only driving performance optimization but also laying the foundation for scalable and resilient superconducting infrastructures.

## Future aspects

The future advancement of REBCO materials remains one of the most dynamic frontiers in materials science and applied technology. Building on the findings summarized in this review, several key directions and opportunities are anticipated to shape the next stage of progress.

First, continued refinement of PLD parameters will be central to further optimizing the superconducting properties and mechanical resilience of REBCO thin films. As shown in the comprehensive summary tables (Tables 2, 3, 4, 5, 6, 7, and 8), comparative benchmarking of deposition conditions and resulting properties will provide an invaluable reference for researchers working to fine-tune their fabrication protocols.

**Table 3:** Summary on YGBCO performance and PLD techniques and parameters used for deposition

Superconductor	Method	Conditions	Critical Temperature	Critical Current Density	Substrate	References
Y_0.5_Gd_0.5_Ba_2_Cu_3_O_7-δ_ (YGBCO)	PLD	KrF excimer laser (λ = 248 nm),; Laser energy fluence: 1.0 J/cm^2^; Substrate - target distance: 30 mm; Repetition frequency of laser: 100 Hz; Oxygen pressure: 200 mTorr; Thickness YGBCO was maintained at 250 nm by control of the deposition time.	4 g/cm^3^ – 91.3 K	4 g/cm^3^ – 5.4 MA/cm^2^ (77 K, self-field)	CeO_2_/MgO/Y_2_O_3_/Al_2_O_3_ buffered C276	Liu et al.^83^
Y_0.5_Gd_0.5_Ba_2_Cu_3_O_7-δ_ (YGBCO) YGBCO/Gd-CeO_2_/YGBCO	PLD	KrF excimer laser (LPX220); Laser pulse repetition rate: 40–150 Hz^239^; Oxygen partial pressure and laser energy were optimized.	NA	140 A at self-field, 77 K (optimal PLD parameters)	CeO_2_/IBAD-MgO/Y_2_O_3_/Al_2_O_3_/Hastelloy C276	Liu et al.^172^
Y_0.5_Gd_0.5_Ba_2_Cu_3_O_7-r_ (YGBCO)	Multi target reel-to-reel PLD with seed layer	From Ref.^83^ KrF excimer laser (λ = 248 nm); Laser energy fluence: 1.0 J/cm^2;^ Target-substrate distance: 30 mm; Repetition frequency: 100 Hz; Oxygen pressure: 200 mTorr; The thickness of the YGBCO films was maintained at 250 nm by control of the deposition time.	NA	77 K, self-field No seed: 3.17 MA/cm^2^; S1 (seed 2 nm): 3.33 MA/cm^2^ S2 (seed 7 nm): 3.33 MA/cm^2^; S3 (seed 15 nm): 4.17 MA/cm^2^; S4 (seed 30 nm): 3.67 MA/cm^2^	CeO_2_/IBAD-MgO/Y_2_O_3_/Al_2_O_3_/Hastelloy C276	Yao et al.^151^
Y_0.5_Gd_0.5_Ba_2_Cu_3_O_7-δ_ (YGBCO) with 5mol % BaHfO_3_ (BHO)	MPMT-PLD	Laser power 380 W	>90 K	>300 A/cm (77 K, self-field)	CeO_2_/IBAD-MgO/Y_2_O_3_/Al_2_O_3_/Hastelloy	Li et al.^179^
Y_0.6_Gd_0.6_Ba_2_Cu_3_O_7_	Reel-to-reel MPMT-PLD	KrF excimer laser (LAMBDA 300 K); Deposition temperature: 900°C; High traveling speed of the tape: ∼100–150 m/h; Oxygen partial pressure: 500 mTorr; Laser energy: 500–700 mJ; Laser pulse repetition rate: 200 Hz; For improved PLD a thermal shield under the heating cylinder to minimize the temperature variation was added.	∼87 K	77 K, self-field: 380 A/cm	CeO_2_/IBAD-MgO/Y_2_O_3_/Al_2_O_3_/Hastelloy	Zhao et al.^146^
Y_0.5_Gd_0.5_Ba_2_Cu_3_O_7−δ_ (YGBCO) doped with 5 mol % BaHfO_3_ (BHO)	PLD seed layer technique	KrF excimer laser (λ = 248 nm); Laser energy density: 1.0 J/cm^2^; Substrate-target distance: 40 mm; Oxygen pressure: 200 mTorr; Firstly, 2-nm-thick 5-mol% YGBCO+BHO seed layer was fabricated at a laser repetition rate of 10 Hz and substrate temperatures (710°C–820°C). Secondly, 200-nm-thick YGBCO+BHO upper layers were grown at a laser repetition rate of 160 Hz of one plume and 820°C.	Best performing (790°C with seed layer): 90 K	Best performing (790°C with seed layer, in self-field at 77 K): 4 MA/cm^2^	CeO_2_ (001)/IBAD- MgO/Y_2_O_3_/Al_2_O_3_/Hastelloy C276	Liu et al.^81^

**Table 4:** Summary on EuBCO performance and PLD techniques and parameters used for deposition

Superconductor	Method	Conditions	Critical Temperature	Critical Current Density	Substrate	Reference
EuBa_2_Cu_3_O_7-α_ (EuBCO) with 3.5 mol % BaHfO_3_ (BHO)	VLS- PLD	A 200 W industrial XeCl excimer laser (λ = 308 nm); Pulse repetition rate: 177 Hz; Pulse energy: 600 mJ; Deposition temperature: 1095°C–1145°C; Oxygen pressure: 600 mTorr; Oxygen flow: 10 sccm; Target - substrate distance: 100 mm	NA	EuBCO+BHO (77 K, 3 T, 1.35 μm) VLS high dep. rate: ∼48.7 A/cm- width; VLS low dep. rate: ∼53 A/cm- width; VS high dep. rate: ∼30 A/cm- width;	CeO_2_/LaMnO_3_/IBAD-MgO/Y_2_O_3_/Gd_2_Zr_2_O_7_/HatelloyTM C-276 substrates	Ibi et al.^153^
EuBa_2_Cu_3_O_y_ (EuBCO)	VLS- PLD	Deposition temperature: 1075°C (VS mode) and at 1090°C (VLS mode), which were controlled temperatures.	NA	77 K, self-field: VS mode: 820 A/cm; VLS mode: 955 A/cm 77 K, 3 T, min: VS mode: 107 A/cm; VLS mode: 141 A/cm	CeO_2_/LaMnO_3_/MgO/Y_2_O_3_/Gd–Zr–O/Hastelloy	Yokoe et al.^155^
EuBa_2_Cu_3_O_7-α_ (EuBCO) with BaHfO_3_ (BHO)	PLD multi- plume and multi-turn (MPMT) with VLS	200 W industrial XeCl excimer (λ = 308 nm); Typical pulse duration (FWHM): 22 ns; Pulse energy: 500–600 mJ; Pulse repetition rate: 177 Hz; The laser energy density directly above the sintered the BHO+EuBCO: 2–3 J/cm^2^; To realize the VLS growth mode EuBa_2_Cu_3.2_O_X_ containing 3.5–10 mol % doped BHO target was used; Deposition rate:12 nm/s; Deposition temperatures: 1035°C–1150°C; Transfer speed: 30 m/h; Substrate temperature of CeO_2_: 880°C; The oxygen pressure: 600 mTorr; Target-substrate distance: 90–100 mm.	10 mol % BHO-doped EuBCO (1150°C deposition temp., 280°C annealing temp.) ∼93.9 K	5 mol % BHO-doped EuBCO (self-field, 77 K, 250°C annealing temp.) ∼5.5 MA/cm^2^; 5 mol % BHO-doped EuBCO (3T, 77 K, 250°C annealing temp.) ∼0.62 MA/cm^2^	CeO_2_ (300–700 nm)/LaMnO_3_ (7 nm)/IBAD-MgO (5 nm)/Y_2_O_3_ (14 nm)/Gd_2_Zr_2_O_7_ (56 nm)/Hastelloy C276 (100 μm)	Ibi et al.^154^
EuBa_2_Cu_3_O_7-α_ (EuBCO) with BaHfO_3_ (BHO)	PLD multi-coating system	KrF excimer laser (λ = 248 nm); Laser power: 50 W (330 mJ, 150 Hz); Target-substrate distance: 90 mm (out-of-plumes condition); Oxygen pressure: 80 Pa; Substrate surface temperature: 860°C; Deposition speed: 3 nm/s	NA	EuBCO + BHO film with 3.6 μm thickness ∼141 A/cm-width (77 K, 3 T)	PLD-CeO_2_/Sputter-LMO/IBAD-MgO/Sputter-Y_2_O_3_/IBS-Gd_2_Zr_2_O_7_/Hastelloy	Yoshida et al.^140^
EuBa_2_Cu_3_O_y_ (EuBCO) with BaHfO_3_ (BHO)	Reel-to-reel multi plume PLD	A 200 W industrial XeCl excimer laser (λ = 308 nm); Pulse repetition rate:177 Hz; Pulse energy: <600 mJ; The plume was divided into 4 plumes; Oxygen pressure: 600 mTorr; Oxygen flow: 10 sccm	NA	5.1 MA/cm^2^ for 0.45 μm in thickness (77 K, self-field)	CeO_2_/LaMnO_3_/IBAD-MgO/Y_2_O_3_/Gd_2_Zr_2_O_7_/Hastelloy C-276	Ibi et al.^44^
EuBa_2_Cu_3_O_y_ (EuBCO) with BaHfO_3_ (BHO)	Reel-to-reel multi-plume PLD	KrF excimer laser (λ = 248 nm); Laser power: 50W (330 mJ, 150 Hz); Target-substrate distance: 90 mm (out-of-plumes condition); Oxygen pressure: 80 Pa; Substrate temperature: 870°C; Deposition speed: 3 nm/s	NA	77 K, self-field: 1.99 MA/cm^2^; 77 K, 0.3 T: 0.961 MA/cm^2^ 77 K, 3 T: 0.286 MA/cm^2^	CeO_2_/LaMnO_3_/IBAD-MgO/Gd_2_Zr_2_O_7_/Hastelloy C-276	Yoshida et al.^141^
EuBa_2_Cu_3_O_y_ (EuBCO)with BaHfO_3_ (BHO)	Multi-plumePLD	Plane plume: Target-substrate distance shortened: 70 mm; Number of plumes increased: 18 plumes; 200 W industrial XeCl excimer laser (λ = 308 nm); Pulse repetition rate: 300 Hz; Pulse energy: 600–700 mJ; The deposition temperature: 930°C; Transferring speed: 60 m/h; Oxygen pressure: 800 mTorr; Oxygen flow: 10 sccm; The multi-filamentary structure was fabricated by using the KrF excimer laser (λ = 248 nm)	NA	Distribution for 20.1 m long (mulfilament): 0–2 mA/cm^2^	CeO_2_/LaMnO_3_/IBAD-MgO/Y_2_O_3_/Gd_2_Zr_2_O_7_/Hatelloy C-276	Ibi et al.^142^
EuBa_2_Cu_3_O_y_ (EuBCO) with BaHfO_3_ (BHO)	Hot-wall PLD	High growth rate condition: Growth rate: 20–30 nm/s, comparable to standard non-doped samples. Growth rate of REBCO was controlled by PLD parameters like laser pulse energy and repetition rate, target-to-substrate configurations, atmosphere, etc.	NA	1756 and 1786 A/cm (30 K, 2 T)	CeO_2_/MgO/Y_2_O_3_/Al_2_O_3_/Hastelloy	Fujita et al.^161^
EuBa_2_Cu_3_O_y_ (EuBCO) with BaHfO_3_ (BHO)	Hot-wall PLD	Condition A: “High-Jc condition”: Typical growth rate: 5–7 nm/s; Condition B: “High-rate condition”: Typical growth rate: 20–30 nm/s, comparable to standard non-doped samples.	NA	PLD condition A: ∼3.1 MA/cm^2^ (77 K, self-field) ∼14 MA/cm^2^ (30 K, 2 T) PLD condition B: ∼1.8 MA/cm^2^ (77 K, self-field) ∼7.2–7.4 MA/cm^2^ (30 K, 2 T)	CeO_2_/MgO/Y_2_O_3_/Al_2_O_3_/Hastelloy	Iijima et al.^163^
EuBa_2_Cu_3_O_y_ (EuBCO) with BaHfO_3_ (BHO)	Hot-wall PLD	Fast: Deposition rate: 20–30 nm/s; Thickness: 2.2 μm; Tape length: 640 m; Slow: Deposition rate: 5–15 nm/s; Thickness: 1.1 μm; Tape length: 10 m;	Fast: 91.2 K Slow: 91.8 K	77 K, self-field: Fast: ∼2 MA/cm^2^ Slow: ∼2 MA/cm^2^	CeO_2_/IBAD-MgO/Y_2_O_3_/Al_2_O_3_/Hastelloy	Fujita et al.^164^
Eu_1_Ba_2_Cu_3_O_7_ with BaHfO_3_ (BHO)	PLD	Excimer lasers with the max power: 300 W; Laser energy per pulse: 500–1000 mJ; Repetition rate: 280–300 Hz; Film thickness: 0.9–2.7 μm	NA	Average Jc: 1.9 MA/cm^2^ (77 K, self-field)	CeO_2_/LaMnO_3_/MgO/Y_2_O_3_/Al_2_O_3_/Hastelloy	Jiang et al.^178^

**Table 5:** Summary on GdBCO performance and PLD techniques and parameters used for deposition

Superconductor	Method	Conditions	Critical Temperature	Critical Current Density	Substrate	Reference
GdBa_2_Cu_3_O_7-α_ GdBCO, (Y,Gd)BCO, and EuBCO+BHO	RACH-PLD	KrF excimer laser (LAMBDA 300 K); Oxygen partial pressure: 300∼500mTorr; Laser energy: 500∼700 mJ; Laser pulse repetition rate: 260∼280 Hz Deposition temperature: 700∼900°C; Tape traveling speed: 100–200 m/h	NA	GdBCO 4.5∼3 MA/cm^2^ up to 2 μm thick film (77 K, 0 T)	CeO_2_ (200 nm, PLD)/LMO (20 nm, sputtering)/MgO (5 nm, IBAD)/Y_2_O_3_ (20 nm, sputtering)/Al_2_O_3_ (80 nm, sputtering)/Hastelloy (50 μm, electropolishing)	Jiang et al.^180^
GdBa_2_Cu_3_O_7_; EuBCO + BaHfO_3_; YGdBCO	RACH-PLD	KrF excimer laser; Oxygen partial pressure: 300–500 mTorr; Laser energy: 500–700 mJ; Laser pulse repetition rate: 260–280 Hz; Layer thickness: 1.5–2.5 μm	NA	GdBa_2_Cu_3_O: 520 A/cm-width (77 K, self-field) EuBCO +BaHfO_3_ and YGdBCO: 280 A/4 mm-width (30 K, 5 T) 560 A/4 mm-width (4.2 K, 10 T)	CeO_2_/LaMnO_3_/MgO/Y_2_O_3_/Al_2_O_3_/Hastelloy	Zhao et al.^181^
GdBa_2_Cu_3_O_7−X_ (GdBCO or Gd123)	Hot-wall PLD	A 180 W KrF (λ = 248 nm) excimer laser; The laser beam was scanned in the vertical direction along lanes on the target; In the hot-wall heating system, the surface of the template was heated by radiation in the heater box. Furthermore, high-speed depositions were performed by a tandem laser system with a laser power of 360 W.	NA	3 MA/cm^2^ (360 W laser power) (77 K, self-field)	CeO_2_/Gd_2_Zr_2_O_7_ (GZO)/Hastelloy	Kakimoto et al.^162^
GdBa_2_Cu_3_O_7-x_ (GdBCO) or EuBa_2_Cu_3_O_7-x_ (EuBCO) + BaMO_3_ (M = Hf, Zr)	Hot-wall PLD	NA	NA	77 K, self-field: GdBCO (1.9 μm) 219 A; GdBCO (1.8 μm) 5.0 mol % BZO 154 A; GdBCO (1.3 μm) 3.5 mol % BZO 91 A; EuBCO (1.1 μm) 3.5 mol % BZO 88 A; Ic decreases with increasing strain, BMO-doped REBCO is more strain-sensitive	CeO_2_/MgO/Y_2_O_3_/Al_2_O_3_/Hastelloy	Fujita et al.^165^
GdBa_2_Cu_3_O_y_ (GdBCO) with BaZrO_3_ (BZO)	In-plume (IP) PLD	KrF excimer laser; Substrate temperature: 780°C–850°C Oxygen partial pressure: and 300–500 mTorr; After deposition the films were slowly cooled down to room temperature in a chamber with pure oxygen to 600 Torr without further post-annealing.	NA	Ic: 135 A/cm (3 T, 77.5 K); Ic: 700–1000 A/cm (self-field, 77.5 K)	CeO_2_/Gd_2_Zr_2_O_7_/Hastelloy C-276	Lee et al.^147^
GdBa_2_Cu_3_O_y_ (GdBCO)with BaZrO_3_ (BZO)	In- plume (IP)-PLD	KrF excimer laser; Repetition rate: 40 Hz; Laser energy density: 0.02–0.04 J/mm^2^ Substrate-target distance: 50–90 mm; Substrate temperature: ∼1073 K; Oxygen partial pressure: ∼46.7 Pa; After the deposition, the samples were oxygen annealed *in situ*, slowly cooled down to room temperature in pure oxygen	∼93.5 K	Average ∼1.5 MA/cm^2^ (77.5 K, self-field)	PLD-CeO_2_/IBAD-Gd_2_Zr_2_O_7_/Hastelloy	Chikumoto et al.^148^
GdBa_2_Cu_3_O_y_ (GdBCO)	In-plume (IP) MPMT-PLD	A 200 W industrial XeCl excimer laser (λ = 308 nm); Pulse repetition rate: 300 Hz; Pulse energy: >600 mJ; The laser repetition rate: 300 Hz was divided into 18 plumes (multi plume). Commercial sintered off-stoichiometric GdBa_1.8_Cu_3_O_7-x_ targets with a diameter of 6 inch were used.	NA	In plume, 77 K, self-field: (0.45 μm, 60 m/h) ∼5.6 MA/cm^2^; (0.9 μm, 30 m/h) ∼4.2 MA/cm^2^;	CeO_2_/LaMnO_3_/IBAD-MgO/Gd_2_Zr_2_O_7_/Hastelloy C-276 substrates	Ibi et al.^149^
GdBa_2_Cu_3_O_7_ (GdBCO)	In-plume (IP) reel-to-reel PLD	80 W KrF excimer laser with a single-turn and four-plume; Deposition area: 1 cm × 6.5 cm; Target: GdBa_2_Cu_z_O_y_ (z = 3.0, 3.2, 3.4); Substrate temperature: 800°C; Oxygen pressure: 400 mTorr; Target-substrate distance (Dts): 5.5, 7.0, 9.0 cm; Traveling speed: 10, 20 m/h	NA	77 K, self-field: 5.5 cm (Dts) ∼1.4 MA/cm^2^; 7 cm (Dts) ∼2.6 MA/cm^2^; 9 cm (Dts) ∼2.8 MA/cm^2^;	PLD-CeO_2_/IBAD-Gd_2_Zr_2_O_7_ (GZO)/Hastelloy C276™	Miura et al.^150^
GdBa_2_Cu_3_O_7-α_ (GdBCO) with BaZrO_3_ (BZO)	MPMT-PLD	MPMT-PLD with multi-layer deposition was used with 4 laser plumes; Deposition temperature: 850°C–900°C; Oxygen partial pressure: 600 mTorr; Energy of laser beam: 500 mJ; Repetition rate of laser pulse: 160 Hz,	NA	51.4 m–204 A (77 K and self-field) and ∼21.6 A (77 K and 3 T)	CeO_2_ (cap)/Gd_2_Zr_2_O_7_ (GZO) buffered Hastelloy C-276 tape	Ibi et al.^145^
GdBa_2_Cu_3_O_7_ with BaSnO_3_ (BSO) or BaZrO_3_ (BZO)	Reel-to-reel PLD	Layer growth rate of 750 nm/min was used, which is typical for the pilot-scale equipment at SuperOx.	BSO ∼91.9 K BZO ∼91 K	77 K, self-field, (A/12 mm): BSO ∼140 A BZO ∼120 A	CeO_2_/LaMnO_3_/IBAD-MgO/Y_2_O_3_/Al_2_O_3_/Hastelloy	Ovcharov et al.^168^
GdBa_2_Cu_3_O_7_ (GdBCO) with 6 mol % BaSnO_3_ (BSO) nanoparticles	PLD	Dual chamber PLD system with a 130 W LEAP excimer laser; Repetition rate:100–200 Hz; Pulse energy: 500–650 mJ; Oxygen partial pressure: 10–60 Pa Temperature: 600°C–850°C.^72^	∼92 K	77 K, self-field: 141 A (375 nm/min 125 A (560 nm/min)	CeO_2_/LaMnO_3_/IBAD-MgO/LaMnO_3_/Y_2_O_3_ or Al_2_O_3_/Hastelloy	Lao et al.^167^
GdBa_2_Cu_3_O_7-δ_ with BaHfO_3_ (BHO)	PLD	KrF excimer laser (λ = 248 nm); Laser energy: 280–330 mJ; Frequency: 120 Hz (two plumes at 60 Hz); Oxygen pressure: 53–80 Pa; Target–substrate distance: 86–94 mm; Deposition temperature: 1123 K; Tape traveling rate: 20 m/h	NA	0.3 MA/cm^2^ (77 K, 3 T)	CeO_2_/LaMnO_3_/IBAD-MgO/IBS- Gd_2_Zr_2_O_7_/Hastelloy	Maeda et al.^170^
GdBa_2_Cu_3_O_7−δ_ (GdBCO)	Dual-Chamber PLD	LEAP excimer; Deposition rate: 15–30 m/h; Laser energy: 500–650 mJ; Repetition rate: 100–200 Hz; Oxygen partial pressure of 10–60 Pa; Temperature: 600°C–850°C	NA	Ic at 77 K, self-field: 300–500 A (/12 mm wide tape)	CeO_2_/LaMnO_3_/IBAD-MgO//LaMnO_3_/Y_2_O_3_ or Al_2_O_3_/Hastelloy C276™	Lee et al.^72^
GdBa_2_Cu_3_O_7−δ_ (GdBCO) with BaSnO_3_ (BSO) or BaZrO_3_ (BZO)	PLD	Xe-Cl excimer laser (λ = 308 nm); Pulse energy: 700 mJ; Repetition rate: 100, 150 and 200 Hz, which corresponded to 375, 560, and 750 nm/min; Oxygen pressure: 70 Pa; In the deposition zone, the buffered substrate tape was heated by making mechanical contact with a hot Inconel plate kept at a controlled temperature reaching up to 1000°C. Substrate tape speed: 45–60 m/h. After deposition the films were annealed in pure oxygen (420°C, 7 h)	GdBCO+BSO (6%): 375 nm/min ∼91.9 K; 560 nm/min ∼91.9 K GdBCO+BZO (6%): 750 nm/min ∼92 K	77 K, self-field: GdBCO+BSO (6%), 375 nm/min ∼250 A/12 mm; 560 nm/min ∼203 A/12 mm GdBCO+BZO (6%): 750 nm/min ∼140 A/12 mm	CeO_2_/LaMnO_3_/IBAD-MgO/a-Y_2_O_3_/a-Al_2_O_3_/Hastelloy	Chepikov et al.^182^
GdBa_2_Cu_3_O_7−δ_ (GdBCO)	PLD	1.3 μm thick, 3.9 mm wide REBCO layer	NA	155 A (77 K in self-field)	Hastelloy with IBAD-MgO buffer layer	Seiler et al.^184^
GdBa_2_Cu_3_O_y_ (GdBCO)	PLD	NA	Not specified	5.5 μm, 110 m: 937 A/cm-width at 77 K, self-field; 637 A/cm-width at 50 K, 5T; 976 A/cm-width at 40 K, 5T 2.4 μm, 500 m: 600 A/cm-width (77 K, self-field);	CeO_2_ (400 nm)/MgO (5 nm)/Y_2_O_3_ (20 nm)/Al_2_O_3_ (150 nm)/Hastelloy (100 μm or 75 μm)	Fujita et al.^185^
GdBa_2_Cu_3_O_y_ (GdBCO) + BaHfO_3_ (BHO)	PLD alternating target	Substrate temperature: 780°C; Oxygen pressure: 53 Pa; Target-substrate distance: 70 mm; Laser frequency: 1–10 Hz; Laser energy density: 1.5 J/cm^2^; Film deposition rates of GdBCO and BHO were 0.027 nm/pulse and 0.012 nm/pulse.	GdBCO to BHO ablation samples: Pure: 90.7 K 25:1: 90.6 K 20:1: 89.7 K 39:2: 88.7 K 78:4: 89.3 K 15:1: 88.9 K 39:4: 87.8 K 78:8: 88.2 K	77 K, 0 T: Pure: 3 MA/cm^2^ 25:1: 4 MA/cm^2^ 20:1: 6 MA/cm^2^ 78:8: 0.8 MA/cm^2^ 77 K, 5 T: Pure: 0.08 MA/cm^2^ 25:1: 0.1 MA/cm^2^ 20:1: 0.3 MA/cm^2^ 65 K, 0 T: Pure: 7 MA/cm^2^ 25:1: 9 MA/cm^2^ 20:1: 10.5 MA/cm^2^ 39:4: 2 MA/cm^2^ 65 K, 5 T: Pure: 0.5 MA/cm^2^ 25:1: 1 MA/cm^2^ 20:1: 1.5 MA/cm^2^ 39:4: 0.3 MA/cm^2^	LaAlO_3_	Matsumoto et al.^160^

**Table 6:** Summary on SmBCO performance and PLD techniques and parameters used for deposition

Superconductor	Method	Conditions	Critical Temperature	Critical Current Density	Substrate	Reference
Sm_1+x_Ba_2-x_Cu_3_O_y_ (SmBCO)with BaZrO_3_ (BZO)	LTG-PLD	Seed layer of c-axis-oriented SmBCO: Substrate temperature: 830°C; Approximate thickness: 100 nm; Sm/Ba composition of x = 0.08–0.12; For the growth of the 2 vol % BZO-doped SmBCO upper layer Laser beam frequency:10 Hz; Thickness: 600 nm; Sm/Ba substitution quantity of x = 0.04; Low substrate temperature: 740°C–780°C	NA	Mixed target: 2vol %: ∼2 MA/cm^2^ (77 K and 0 T); ∼0.15 MA/cm^2^ (77 K, 3 T); Layer by layer: 2vol %: ∼2 MA/cm^2^ (77 K and 0 T); ∼0.2 MA/cm^2^ (77 K, 3 T);	MgO	Yoshida et al.^156^
Sm_1+x_Ba_2-x_Cu_3_O_y_ (SmBCO)with BaHfO_3_ (BHO)	LTG-PLD	KrF excimer laser (λ = 248 nm); Repetition rate: 10 Hz; Laser energy density: 2.0 J/cm^2^; Substrate-target distance: 52.5 mm; Oxygen partial pressure: 400 mTorr; Alternating target technique to add BHO to the SmBCO films.	NA	LAO, 3 T: ∼20 MA/cm^2^ (40 K, 90 deg. Ts = 720°C); ∼1 MA/cm^2^ (77 K, 0 deg. Ts = 750°C); IBAD, LTG: ∼40 MA/cm^2^ (4.2 K, 90 deg., 5 T); ∼30 MA/cm^2^ (4.2 K, 90 deg., 15 T);	LaAlO_3_ (LAO) and IBAD tape (CeO_2_/LaMnO_3_/IBAD–MgO/Y_2_O_3_/Gd–Zr–O/Hastelloy)	Yoshida et al.^157^
Sm_1+x_Ba_2-x_Cu_3_O_6+y_ (SmBCO)	PLD low-temperature growth (LTG)	ArF laser (λ = 193 nm); Frequency: 10 Hz; Oxygen pressure: 0.4 Torr; Seed temperature: 830°C; Substrate temperature: 700°C–800°C; After thin film deposition, oxygen gas was introduced up to 20 Torr and the film was cooled rapidly.	91.5–92.5 K	0.28 MA/cm^2^ (77K, 5T)	MgO (100)	Yoshida et al.^158^
SmBa_2_Cu_3_O_y_ (SmBCO) + BaHfO_3_ (BHO)	PLD low-temperature growth (LTG)	KrF (λ = 248 nm) excimer laser; Repetition rate: 10 Hz; Laser energy density 2.0 J/cm^2^; Substrate-target distance: 45 mm; In the LTG films, a pure SmBCO seed layer was deposited at 850°C with a thickness of about 50 nm on a LAO substrate, and then an upper layer of SmBCO with BHO was grown at T_upper_ = 750°C to a thickness of 300–450 nm on it.	LTG max: 92 K PLD conventional max: 93 K	Not specified	LaAlO_3_	Miura et al.^159^
Sm_1+x_Ba_2-x_Cu_3_O_6+σ_ (x = 0.04, 0.08 and 0.12)	PLD	ArF excimer laser (λ = 193 nm); Laser energy density: ∼1 J/cm^2^ (focused spot area: 3 × 2 mm^2^); Repetition rate: 10 Hz.	Best performing 91.5 K	NA	MgO (100)	Sudoh et al.^134^

**Table 7:** Summary on ErBCO performance and PLD techniques and parameters used for deposition

Superconductor	Method	Conditions	Critical Temperature	Critical Current Density	Substrate	Reference
ErBa_2_Cu_3_O_7-x_ (ErBCO) + BaZrO_3_ (BZO)	PLD	ArF excimer; Layers thickness: 700 nm (1.5 wt % BZO), 400 nm (3.5 wt % BZO)	88.8 K (1.5 wt % BZO), 88.0 K (3.5 wt % BZO), 90.2 K (0 wt % BZO)	77.3 K: 1.5 wt % BZO, max Jc: 1 T–0.3 MA/cm^2^; 2 T–0.07 MA/cm^2^; 3.5 wt % BZO, max Jc: 1 T–0.35 MA/cm^2^; 2 T–0.24 MA/cm^2^;	SrTiO_3_	Namba et al.^169^
ErBa_2_Cu_3_O_7-δ_ (ErBCO) + BaNb_2_O_6_ (BNO, 1.5 wt %)	PLD	ArF excimer laser (λ = 193 nm); Pulse frequency: 1 Hz; Laser energy: 400 mJ; Substrate temperature: 710°C–760°C (pure ErBCO – 730°C); Oxygen pressure: 400 mTorr; After the deposition, they were annealed at 450°C for 15 min in a flowing O_2_ gas and then were cooled down rapidly to room temperature.	87.65 K (Td = 730°C), 90.3 K (Td = 760°C)	0.5 MA/cm^2^ (Td = 730°C), 0.98 MA/cm^2^ (Td = 760°C) at 77 K, self-field	SrTiO_3_ (100)	Yuki et al.^139^
ErBa_2_Cu_3_O_7-σ_ (ErBCO) with BaNb_2_O_6_ (BNO)	PLD	Ar-F excimer laser (λ = 193 nm); Growth temperature: 710°C–760°C; Laser energy: 400 mJ; Laser frequency: 1 Hz; Oxygen pressure: 400 mTorr; Ex situ post-annealing: 450°C for 15 min in flowing oxygen after the film growth. After the O_2_ annealing, the film was cooled rapidly to room temperature.	88–90 K	0.5 MA/cm^2^ (self-field, 77 K); Enhanced Jc in magnetic fields, particularly at 710°C	SrTiO_3_ (STO)	Kai et al.^137^
ErBa_2_Cu_3_O_7−δ_ (ErBCO) + Ba(Er_0.5_Nb_0.5_)O_3_	PLD	Ar-F excimer laser (λ = 193 nm); Growth temperature: 710°C–760°C; Laser energy: 400 mJ/pulse; Laser frequency: 1 Hz; Oxygen pressure: 400 mTorr; *ex situ* post-annealing was performed at 450°C for 15 min in flowing oxygen after the film growth.	T growth 710°C: 87.5 K T growth 760°C: 90.3 K	T growth 710°C, self-field, 77 K: 0.5 MA/cm^2^ T growth 760°C, self-field, 77 K: 0.98 MA/cm^2^ T growth 710°C, 5 T, 77 K: ∼0.1 MA/cm^2^ T growth 760°C, 5 T, 77 K: ∼0.05 MA/cm^2^	SrTiO_3_	Kai et al.^138^
(Eu,Er)Ba_2_Cu_3_O_7_ ((Eu, Er)BCO) + BaHfO_3_ (BHO)	PLD reel-to-reel	PLD conditions not specified; O^2+^ irradiation (1×10^13^–5×10^13^ ions/cm^2^); Post annealing at 250°C, 5 h	1×10^13^ ions/cm^2^ (the best perf.): 91.7 K	1×10^13^ ions/cm^2^ (the best perf.): 77 K, self-field: ∼5 MA/cm^2^ 77 K, 3 T: ∼0.8 MA/cm^2^ 65 K, self-field: ∼10 MA/cm^2^ 65 K, 3 T: ∼1.8 MA/cm^2^	CeO_2_/LaMnO_3_/IBAD-MgO/Y_2_O_3_/Gd_2_Zr_2_O_7_/Hastelloy	Suzuki et al.^152^

**Table 8:** Summary on other REBCOs performance and PLD techniques and parameters used for deposition

Superconductor	Method	Conditions	Critical Temperature	Critical Current Density	Substrate	Reference
Ho_0.75_RE_0.25_Ba_2_Cu_3_O_7-σ_ (HoREBCO, RE = Dy, Er, Yb)	PLD	Oxygen atmosphere; Substrate temperature: 760°C–820°C; Laser energy density: 210 mJ; At last, the samples were annealed in flowing O_2_ gas at 500°C for 10 h.	NA	77 K, self-field: ∼73 A (HoBCO 770°C); ∼87 A (HoDyBCO, 780°C); ∼84 A (HoErBCO, 790°C); ∼75 A (HoYbBCO, 800°C)	CeO_2_/IBAD-MgO/Y_2_O_3_/Al_2_O_3_/C276 hastelloy	Zheng et al.^171^
(Y_1-x_Ho_x_)Ba_2_Cu_3_O_z_ ((Y,Ho)BCO)	PLD	ND:YAG laser (λ = 266 nm); Laser pulse: 10 Hz; Pulse duration: 18 ns; Target-substrate distance: 30–40 mm; Substrate temperature: 800°C–870°C; Oxygen pressure: 40–50 Pa; Pump energy: 35–45 J; Laser spot area: 0.021 cm^2^; Target rotation speed: 20 rpm; Target-to-lens distance: 50 cm; After deposition, post-annealing at 450°C for 30 min in a flowing oxygen gas.	X = 0: 90 K X = 0.3: 87 K X = 0.5: 89 K X = 0.7: 89 K X = 1: 89 K	77.3 K, self-field: X = 0: 1.2 MA/cm^2^ X = 0.3: 1.45 MA/cm^2^ X = 0.5: 0.14 MA/cm^2^ X = 0.7: 1.26 MA/cm^2^ X = 1: 0.83 MA/cm^2^	SrTiO_3_ (100)	Silva et al.^117^
TmBa_2_Cu_3_O_7−x_	PLD	KrF excimer (λ = 248 nm); Substrate temperature: 745°C–805°C; Oxygen partial pressure: 100–600 mTorr; Target-substrate distance: 65 mm; 6000 shots of laser at the rate of 5 Hz; Fluence: 3.2 J/cm^2^ in an area of 5 × 1 mm^2^; After the deposition, the films were cooled to 500°C with cooling rate of 20°C/min, and then the chamber was filled with 550 Torr of pure oxygen gas and held for 30 min for *in situ* oxygen annealing, followed by subsequent cooling to 300°C with cooling rate of 10°C/min, and finally cooled to room temperature.	86 K (STO)	77 K, self-field: 4.5 MA/cm^2^ (STO substrate); 1 MA/cm^2^ (buffered metal substrate)	STO single crystal NiW/Y_2_O_3_/YSZ/CeO_2_ buffered metal	Ko et al.^135^
NdBa_2_Cu_3_O_7−δ_ (NdBCO)	PLD	XeCl laser (λ = 308 nm); Repetition rate: 10 Hz; Laser energy density: 1.5 J/cm^2^ Substrate-target distance: 6.5 cm; Substrate temperature: 740°C–820°C; 1% O_2_/Ar gas at a deposition pressure: 800 mTorr; *In situ* annealing 500°C O_2_: 500 mTorr; *ex situ* annealing 450°C for 1h in flowing O_2_.	96 K expected	0.13 μm thick 77 K, self-field: Ts (760°C) 3.4 MA/cm^2^; Ts (740°C) 2.4 MA/cm^2^ Ts (820°C) 0.9 MA/cm^2^ 0.13 μm thick 77 K, 1 T, Ts 760°C: 0.8 MA/cm^2^	RABiTS with a configuration of CeO_2_ (30 nm)/YSZ (200 nm)/Y_2_O_3_ (50 nm)/Ni–3 at.% W (50 μm)	Wee et al.^136^
REBCO	Pilot reel-to-reel PLD	Excimer laser power: 300 W; Maximum repetition rate: 300 Hz.	NA	77 K, self-field: 5 MA/cm^2^ (<0.8 μm) 3.6 MA/cm^2^ (<1.4 μm)	CeO_2_/YSZ/IBAD-MgO/Hastelloy C276™ tapes (50 μm)	Li et al.^175^
REBCO	Pilot reel-to-reel PLD	KrF excimer laser (λ = 248 nm); Incident angle between the laser beam and the target surface: 45°; Laser energy: 200 mJ; Repetition rate: 100 Hz; Substrate temperature: 800°C; Oxygen pressure: 200 mTorr.	NA	77 K, self-field: 3.0 MA/cm^2^ (1 μm); 2.5 MA/cm^2^ (2 μm)	CeO_2_/IBAD-MgO/Y_2_O_3_/Al_2_O_3_/Hastelloy C276™	Liu et al.^176^
REBCO	Pilot reel-to-reel PLD	KrF excimer laser (λ = 248 nm); Incident angle between the laser beam and the target surface: 45°; Laser energy: 200 mJ; Repetition rate: 100 Hz; Substrate temperature: 800°C; Oxygen pressure: 200 mTorr	NA	77 K, self-field: YBCO: 5.0 MA/cm^2^ (0.2 μm); 2.6 MA/cm^2^ (1.4 μm); YGBCO: 3.64 MA/cm^2^ (0.7 μm); 2.78 MA/cm^2^ (2.8 μm)	CeO_2_/IBAD-MgO/Y_2_O_3_/Al_2_O_3_/Hastelloy C276™	Liu et al.^177^

**Figure 19:**
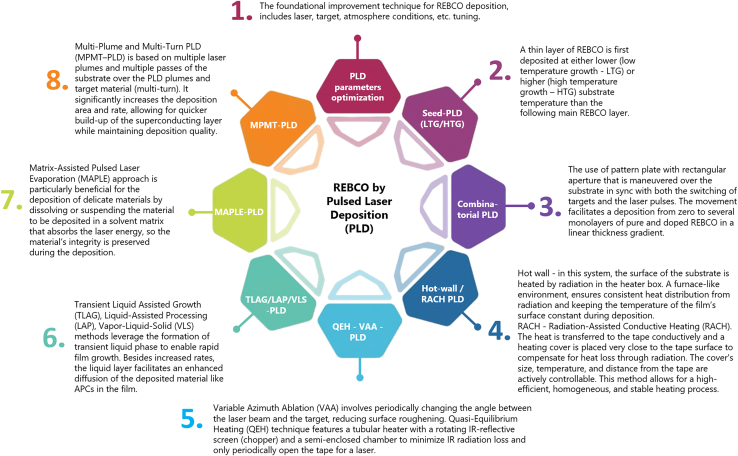
PLD technique and its modifications used for REBCO deposition

Innovation in PLD system design will be equally important. Future work is expected to focus on advanced configurations and modification. Figure 19 presents an infographic synthesizing the modified PLD techniques and their specific technical advantages, serving as a visual guide for navigating this evolving landscape.

**Figure 20:**
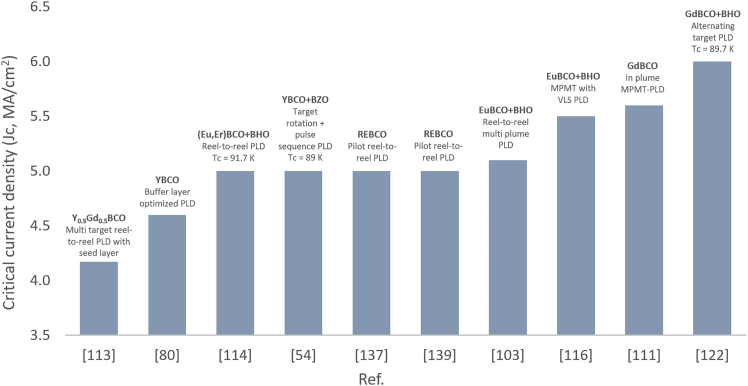
Highest-performing REBCOs based on evaluated data in this review (Self-Field, 77 K)

On the industrial scale, the commercialization of PLD-grown REBCO tapes is poised for significant growth. Figure 20 summarizes the top-performing REBCO superconductors, highlighting those deposited with reel-to-reel MPMT-PLD and other modified PLD methods that achieve the highest critical current densities at 77 K and self-field. As manufacturers push toward greater throughput and cost efficiency, close collaboration between academic and industrial partners will be crucial for translating these successes into widespread, high-impact applications.

Finally, artificial intelligence and ML will increasingly underpin both experimental and industrial efforts, enabling predictive design, rapid optimization of deposition protocols, and accelerated discovery of new material compositions and architectures.

In summary, the future trajectory of REBCO superconductor development via PLD will depend on the convergence of advanced deposition technologies, process optimization, digital innovation, and robust comparative data (as provided in the tables and figures of this review). By leveraging these resources and insights, the field is well positioned to achieve the next generation of high-performance, scalable superconducting materials.

## Acknowledgments

This research was supported by the targeted program no. BR21882402 (2023–2025) from the Ministry of Science and Higher Education of the Republic of Kazakhstan and Faraday Factory Japan LLC.

## Author contributions

Conceptualization, project administration, resources, A.M. and A.N.; investigation, methodology, visualization, A.J.; funding acquisition, A.M., A.N., Z.B., V.P., and S.L.; supervision, Z.B., S.L., and V.P.; validation, S.L. and V.P.; writing – original draft, A.J. and A.M.; writing – review and editing, all.

## Declaration of interests

The authors declare no competing interests.

## Declaration of generative AI and AI-assisted technologies in the writing process

During the preparation of this work the authors used ChatGPT in order to improve the quality of the text. After using this tool/service, the authors reviewed and edited the content as needed and take full responsibility for the content of the publication.
